# Structure of the hexameric fungal plasma membrane proton pump in its autoinhibited state

**DOI:** 10.1126/sciadv.abj5255

**Published:** 2021-11-10

**Authors:** Sabine Heit, Maxwell M. G. Geurts, Bonnie J. Murphy, Robin A. Corey, Deryck J. Mills, Werner Kühlbrandt, Maike Bublitz

**Affiliations:** 1 Department of Biochemistry, University of Oxford, South Parks Road, Oxford OX1 3QU, UK.; 2 Max Planck Institute of Biophysics, Max-von-Laue-Str.3, 60438 Frankfurt am Main, Germany.

## Abstract

The fungal plasma membrane H^+^-ATPase Pma1 is a vital enzyme, generating a proton-motive force that drives the import of essential nutrients. Autoinhibited Pma1 hexamers in the plasma membrane of starving fungi are activated by glucose signaling and subsequent phosphorylation of the autoinhibitory domain. As related P-type adenosine triphosphatases (ATPases) are not known to oligomerize, the physiological relevance of Pma1 hexamers remained unknown. We have determined the structure of hexameric Pma1 from *Neurospora crassa* by electron cryo-microscopy at 3.3-Å resolution, elucidating the molecular basis for hexamer formation and autoinhibition and providing a basis for structure-based drug development. Coarse-grained molecular dynamics simulations in a lipid bilayer suggest lipid-mediated contacts between monomers and a substantial protein-induced membrane deformation that could act as a proton-attracting funnel.

## Teaser

The structure of the hexameric fungal proton pump Pma1 reveals its mechanism of autoinhibition and interactions with lipids.

## INTRODUCTION

The plasma membrane of fungal and plant cells contains a proton-pumping P-type adenosine triphosphatase (ATPase) that maintains the intracellular pH and generates a proton-motive force, driving the import of nutrients by secondary transporters (*1*). The membrane potential generated by the fungal proton pump Pma1 can reach several hundreds of millivolts (*2*), which requires tight coupling of ATP hydrolysis to strictly unidirectional proton transport.

Considerable effort has gone into characterizing Pma1 [reviewed in (*1*)], not least because the enzyme has been identified as a potentially valuable drug target against severe invasive mycoses with high mortality rates, particularly in immunocompromised patients (*3*, *4*). While there is a large body of biochemical data available, an 8-Å map from two-dimensional (2D) electron crystallography has been the highest-resolution structural information available for Pma1 in the past two decades (*5*), severely hampering any structure-based drug development.

Unlike any other known P-type ATPase, Pma1 is a hexamer that localizes to specific ordered microdomains in the fungal plasma membrane (*6*). It is an abundant membrane protein, and the hexamers can form tightly packed, paracrystalline arrays in starving or stressed cells, as revealed by early electron microscopy (EM) studies (*7*, *8*). In addition to the typical P-type ATPase core architecture comprising four domains called N (nucleotide-binding), P (phosphorylation), A (actuator), and M (membrane), Pma1 carries sequence extensions at both its N- and C termini, which have been implicated in regulation and autoinhibition (fig. S1A) (*9*, *10*). The function of the N-terminal extension is still elusive, but the C-terminal autoinhibitory R domain has been postulated to reduce Pma1 activity in starving cells via a clamp-like interaction that immobilizes the cytosolic domains (*7*). Upon glucose sensing, Pma1 is rapidly activated by multiple phosphorylation events in the R domain.

The physiological role of Pma1 hexamers is not yet understood, but in vivo studies in yeast have demonstrated that hexamer formation depends on association with lipids already in the endoplasmic reticulum, which is a prerequisite for surface transport via the secretory pathway (*13*–*15*). In addition to a crucial role of very-long-chain sphingolipids in Pma1 biosynthesis and membrane targeting (*13*), it has also been shown that Pma1 activity in vitro depends strongly on the presence of anionic lipids (*14*, *15*). However, structural evidence to understand these findings has been lacking.

**Fig. 1.**
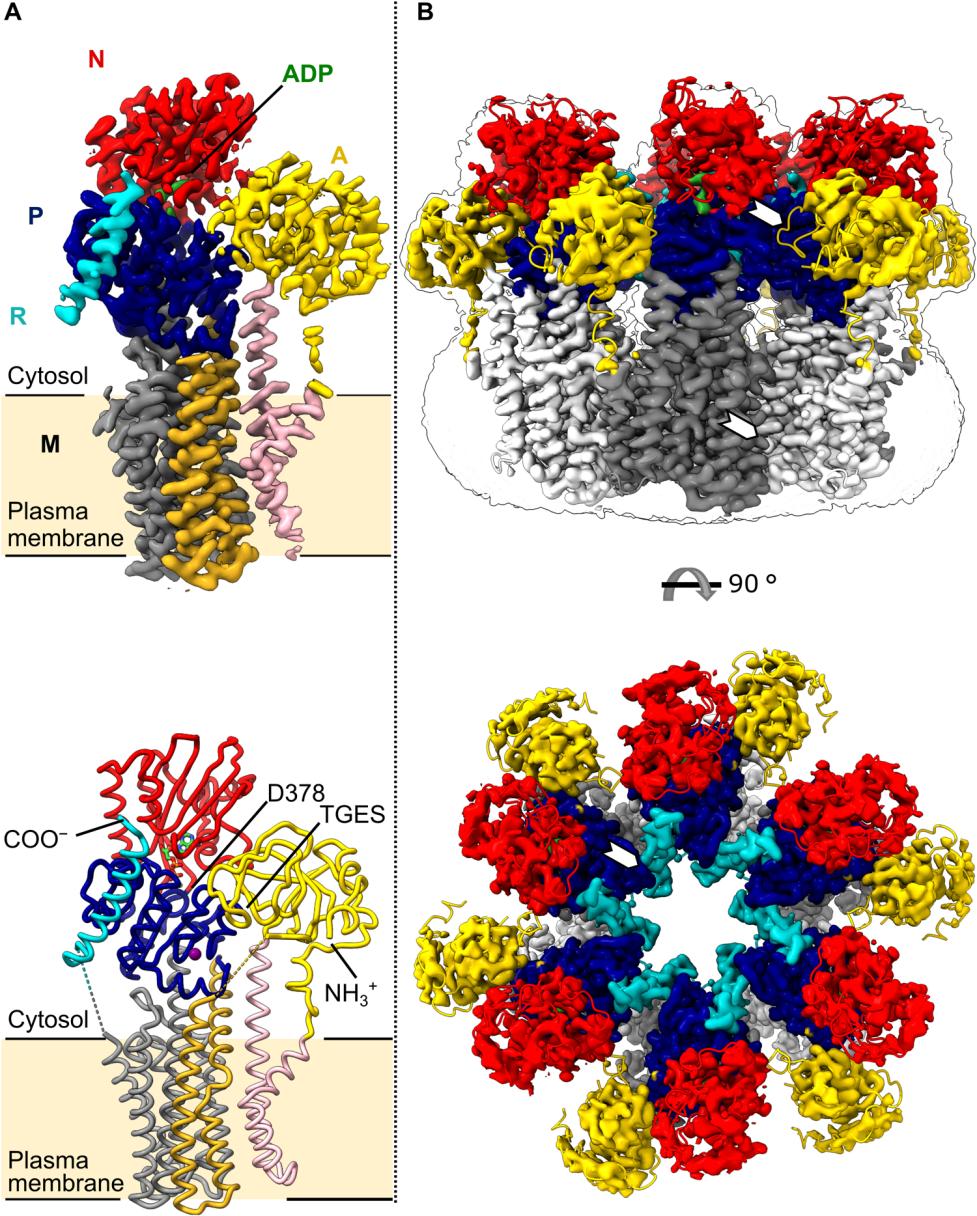
The cryo-EM structure of monomeric and hexameric Pma1. (**A**) Cryo-EM map (top) and structural model (bottom) of the Pma1 monomer subunit. Nucleotide-binding (N) domain in red, actuator (A) domain in yellow, phosphorylation (P) domain in blue, regulatory (R) domain in cyan, and adenosine diphosphate (ADP) in green. The 10 helices of the M domain are colored in pink (M1-M2), gold (M3-M4), and gray (M5-M10). In the bottom panel, ADP is shown as sticks and a potassium ion as a purple sphere; unmodeled loops are indicated by dashed lines. (**B**) Overlay of cryo-EM map and structural model of the Pma1 hexamer in side view (above; with map outline delineating the detergent micelle) and top view (below). The M domains of individual monomers are shown in alternating shades of gray, and cytosolic domains and ADP are colored as in (A). White arrows indicate contact sites between monomers.

We collected single-particle cryo-EM data of the hexameric form of Pma1 from *Neurospora crassa* plasma membrane in its autoinhibited state and obtained a hexamer map at 3.28-Å resolution. An improved map at 3.21-Å resolution for the Pma1 monomer was derived from the same data by symmetry expansion of the hexameric particles followed by focused 3D classification and refinement using a monomer mask (table S1 and fig. S2). An atomic model was built into the monomer map (Fig. 1A) and then extended into a hexameric model by symmetry-assisted placement of five additional copies into the hexamer map (Fig. 1B). The structure reveals the basis for proton transport, autoinhibition, and hexamer formation. Coarse-grained molecular dynamics (MD) simulations in a native-like membrane indicate an accumulation of specific anionic lipids at the monomer interface. A concomitant protein-induced deformation of the lipid bilayer in this region appears to form a cation-attracting funnel and reduce the membrane thickness in the vicinity of the proton-translocating residues. The Pma1 structure is a major advance toward the development of specific inhibitors, and our findings further highlight the important role of lipids in Pma1 function.

## RESULTS

### The structure of Pma1

The Pma1 monomer has a typical P-type ATPase fold, with four conserved domains: the M domain comprising the 10 membrane-spanning α helices M1 to M10, and the 3 cytosolic domains A, N, and P (Fig. 1A and fig. S1A). The additional C-terminal autoinhibitory R domain forms an α helix that folds against the P domain. Our molecular model comprises >90% of all residues, lacking only the N-terminal extension (residues 1 to 65), and two loops (A-M3, residues 269 to 283, and M10-R, residues 881 to 891), for which no continuous density was visible in the map, probably because of flexibility.

**Fig. 2.**
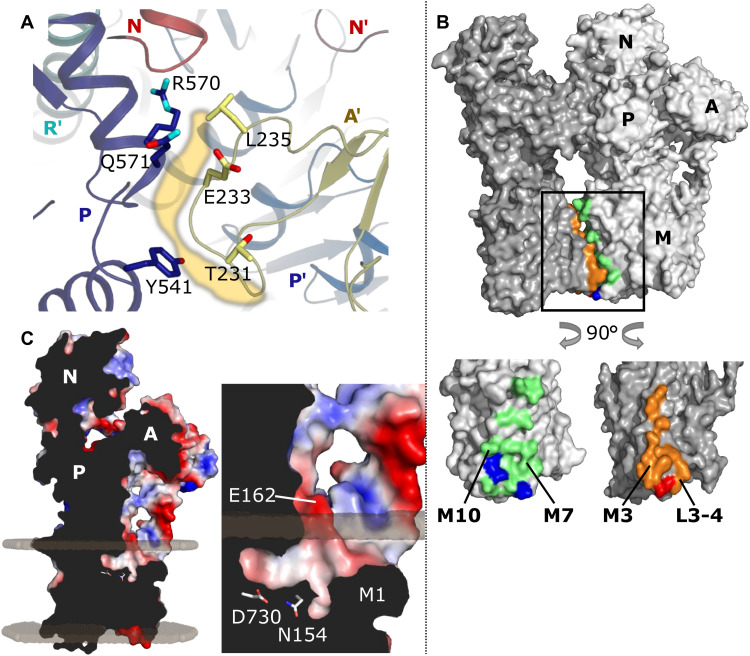
Hexamer contacts and proton entry funnel. (**A**) Interaction interface between the P domain (blue) and the TGES loop of the adjacent monomer’s A domain (A′; yellow). Contact area is shown as yellow shadow; involved side chains are shown as sticks. (**B**) Interaction between M domains. Surface view: Hydrophobic contacts are shown in orange (M3, L3-L4) and light green (M7, M10), and polar contacts are shown in red and blue. (**C**) A cross-section of a Pma1 monomer viewed parallel to the membrane plane, colored by electrostatic surface potential (red, negative; blue, positive). A mainly negatively charged proton entry funnel above M1 leads to the conserved proton acceptor residue pair Asp^730^/Asn^154^ (shown as sticks). The funnel extends below the predicted lipid bilayer boundary (*45*) shown as shaded spheres.

The Pma1 hexamer (Fig. 1B) forms a ring with sixfold symmetry, with a maximum outer diameter of 167 Å and an inner cavity measuring 24 to 55 Å in diameter in the cytosolic and transmembrane regions, respectively. Monomer contacts are mediated by both the cytosolic domains and the transmembrane domains. Specifically, there are two connections between the cytosolic domains: a loose contact between the conserved Thr-Gly-Glu-Ser (TGES) loop in the A domain (residues 231 to 235) and two α-helix termini in the P domain (Tyr^541^, Arg^570^, and Gln^571^) (Figs. 1B and 2A). The second, more extensive contact between the P domains of adjacent monomers is mediated entirely via the autoinhibitory R domain (Fig. 1B, table S2, and see further discussion below). The interactions in the transmembrane region involve residues in M3 of one monomer, and M7 and M10 of the adjacent monomer, augmented by two polar contacts from Ser^316^ and Asn^317^ in L3-L4 to Gln^780^ (M7) and Arg^859^ (M10) (Figs. 1B and 2B and table S3). Most hydrophobic residues involved in the hexamer contact are functionally conserved.

P-type ATPases go through a conformational cycle described by the *E*1/*E*2 (also known as Post-Albers) scheme (fig. S1B), with *E*1 denoting states with a high affinity to the cytosolic substrate ion (H^+^ in the case of Pma1). The domains of Pma1 are arranged in an *E*1 conformation, with the N domain in proximity to the P domain, as required for MgATP-mediated phosphorylation of Asp^378^. The observed state resembles the β,γ-methyleneadenosine 5′-triphosphate (AMPPCP)–bound *E*1 structure of the plant proton pump AHA2 (*16*) and the pre-ion binding, open-to-inside state of SERCA (Mg*E*1) (3.0 and 3.5 Å root mean square deviation over all Cα atoms, respectively; fig. S3) (*17*). The conserved nucleotide binding pocket is occupied by a MgADP molecule (fig. S4A). We added 600 μM AlF*_x_* to the sample before grid preparation, with the aim to stabilize the enzyme in its transition state of phosphorylation, *E*1~P. However, we did not find a clear density for AlF*_x_* in its expected position between the adenosine diphosphate (ADP) β-phosphate and the Asp^378^ carboxyl group. Weaker map features in this region match possible positions of a second Mg^2+^ ion or might indicate a low occupancy of AlF*_x_*. This finding reflects the reduced affinity of autoinhibited, basal state Pma1 to AlF*_x_* compared to the active form (*18*). We have hence captured the basal state before ATP hydrolysis, occupied by excess MgADP.

**Fig. 3.**
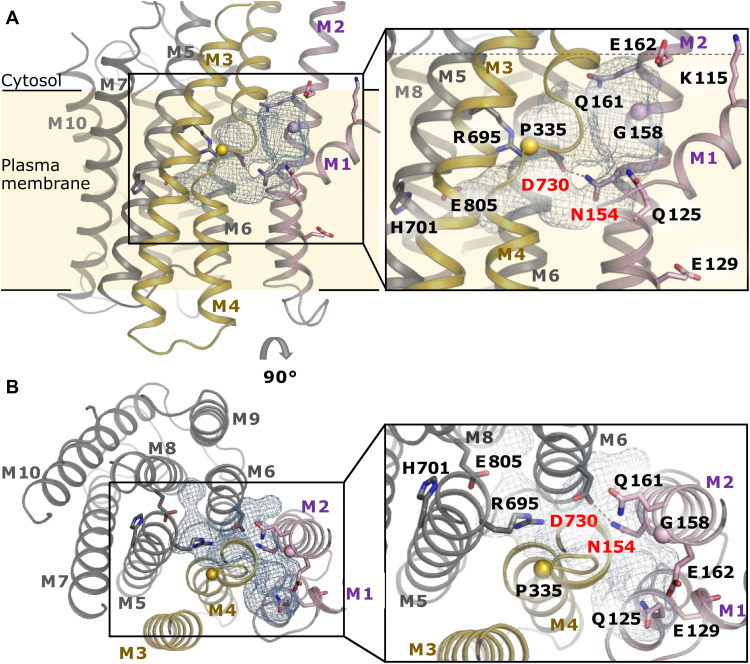
Intramembranous cavity and proton-binding site of Pma1. (**A**) Aqueous cavity between M1, M2, M6, and an unwound region of M4 (the conserved Pro^335^ is shown as a sphere). Important residues for proton transport and the snorkeling K115 (top right corner) are shown as sticks (sphere for glycine). M1-M2 are colored pink, M3-M4 are colored gold, and M5-M10 are colored gray. In the enlarged view, the putative hydrogen bond (2.9 Å) between the proton acceptor/donor Asp^730^ and Asn^154^ (labeled in red) is shown as dashed line. (**B**) Arg^695^ is facing the cavity and could form a salt bridge with Asp^730^ (labeled in red) after proton release. The conserved residues His^701^ and Glu^805^, which are both necessary for proton pumping, interact with each other in the *E*1 state.

As expected for a prephosphorylation *E*1 state, access from the cytosol to the proton-binding site is open in our structure. The M1 helix with its characteristic 90° kink (induced by Pro^123^) is embedded deeply inside the membrane, forming a wide funnel-shaped access toward the H^+^ binding site in M6, similar to the Ca^2+^ entry funnel in SERCA (Fig. 2C, and see more detailed discussion below) (*17*). The mouth of this access pathway is negatively charged because of the conserved Glu^162^ in M2 (Fig. 2C), and it extends into a large aqueous cavity between M1, M2, M6, and an unwound region in M4 around the conserved Pro^335^ (Fig. 3). The cavity is almost twice as large (~746 Å^3^) as in the plant proton pump (~380 Å^3^) (*16*) and can accommodate up to 18 water molecules. While the horizontal, N-terminal part of M1 is amphipathic in SERCA, it is strongly hydrophobic in Pma1, except for Lys^115^, which points upward toward the aqueous cytosolic phase, as a typical “snorkeling” residue (Fig. 3A, inset). The C-terminal part of M1 contains two hydrophilic residues: Gln^125^ at the upper end, pointing into the interior of the protein, and Glu^129^ at the lower end, contacting the membrane lipid environment. In line with its accessibility from the hydrophobic membrane center, Glu^129^ has been identified as the site of covalent dicyclohexylcarbodiimide (DCCD) binding and inhibition of Pma1 (*19*). The structure reveals that DCCD might lock M1 in this open conformation, hence preventing proton occlusion and transport. While the physiological role of Glu^129^ is currently unknown, it is conserved throughout plasma membrane H^+^- and Ca^2+^-pumping P-type ATPases.

The proton-acceptor/donor in Pma1 is the conserved aspartate residue Asp^730^ in M6 (Fig. 3). This residue corresponds to Asp^684^ in AHA2 and the Ca^2+^-coordinating residue Asp^800^ in SERCA and has been shown to be essential for proton transport and *E*1→*E*2 transitions in Pma1 (*20*, *21*). Asp^730^ is situated adjacent to the internal cavity and opposite the equally highly conserved Asn^154^ in M2, at a distance suitable for capturing a proton within a hydrogen bond (2.9 Å between the aspartate carboxyl and the asparagine amide). Proton release to the extracellular side has been suggested to be driven by a conformational change that breaks this hydrogen bond and instead allows the formation of a salt bridge between the deprotonated aspartate and an adjacent arginine residue (Arg^655^ in the plant H^+^-ATPase AHA2) while concomitantly opening up the exit pathway between M1, M4, and M6 (*16*, *22*). The arginine has also been suggested to act as a positively charged “gatekeeper” preventing proton reentry into the cavity. In Pma1, the equivalent of Arg^655^ in AHA2 is His^701^ in M7, which would not be able to fulfill this role—a fact that has raised doubts about the similarity of the mechanisms of the two proton pumps. In our structure, the side chain of His^701^ points away from the proton-binding site, interacting with Glu^805^ in M8 (Fig. 3). The precise role of this residue pair is currently unclear, but both residues are necessary for proton pumping in Pma1 (*23*). The key functional role postulated for Arg^655^ in AHA2 is likely taken over by Arg^695^ in Pma1, which is strictly conserved in fungi and positioned 2.5 helix turns up from His^701^, facing the ion-binding site (Fig. 3, A and B).

**Fig. 4.**
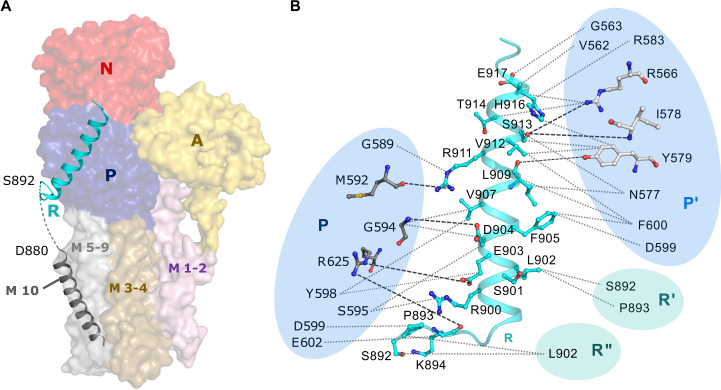
The autoinhibitory R domain and its interaction network. (**A**) The R domain (cyan cartoon) is situated adjacent to the P domain (blue surface). It is connected to M10 (gray cartoon) via a short region of disorder (dashed line). (**B**) Schematic view of intra- and intermolecular interactions mediated by the R domain (cyan). Left: Intramolecular contact between the R and P domains. Right: Intermolecular contact between the R domain and the adjacent monomer’s P domain (P′), and to two adjacent monomers’ R domains (R′ and R″). P/P′ domain residues involved in polar contacts are shown as dark or light gray sticks, respectively. Dotted lines indicate hydrophobic interactions.

The C-terminal autoinhibitory R domain extends from the cytosolic end of M10 into a brief region of disorder and then forms a mainly α-helical structure that lies adjacent to the P domain (Fig. 4A), with which it forms several polar interactions as well as a surprisingly large number of hydrophobic contacts, also involving residues in the P domain of the adjacent monomer (Fig. 4B). The distance across the unmodeled gap between the C terminus of M10 and the N termini of two adjacent R helices is very similar, preventing an unambiguous assignment of R domains to their respective monomers. The current assignment is based on a short extension at the N terminus of the R helix that points toward one of the two M10 termini (fig. S4B).

### Local lipid accumulation and protein-induced membrane deformation

**Fig. 5.**
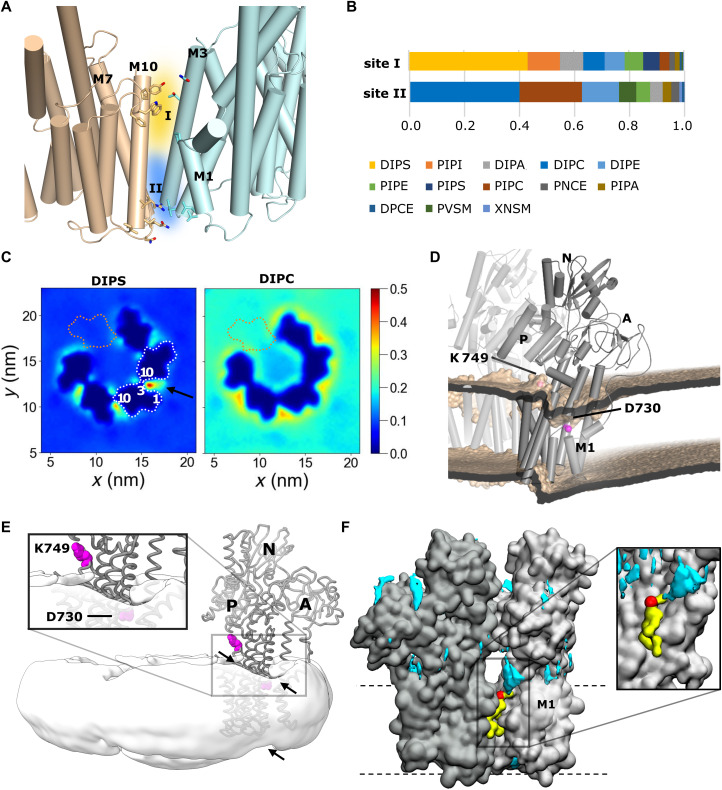
Lipid interactions of Pma1. (**A**) Putative lipid binding sites (I and II) at the monomer interface, suggested by MD simulations. (**B**) Fractional interaction times of lipids in sites I and II, defined as the number of simulation frames in which a lipid is within 0.6 nm of a given site. (**C**) Simulated lipid densities for DIPS and DIPC. Arrow in DIPS points at the pool clustering outside the monomer interfaces. Values correspond to average number of molecules per cubic nanometer and do not account for the respective membrane composition fraction. White dotted lines and numbers indicate monomer outlines and transmembrane helix positions, respectively. Orange dotted lines indicate the omitted sixth monomer. (**D**) Side view of the coarse-grained bilayer deformation at the monomer interface. The bilayer (wheat) is shown as the average phosphate position across five simulation repeats. An atomistic Pma1 model was aligned for analysis. (**E**) Cryo-EM map of hexameric Pma1, filtered to 12 Å, showing only the micelle after masking the protein map in Coot (*14*). The Cα trace of one monomer is shown for reference, with the side chains of D730 and K749 highlighted in magenta. The micelle is locally distorted near the monomer interface (arrows). (**F**) Locally increased density of Na^+^ ions (cyan) around the monomer interface. A representative DIPS molecule in site I is shown in yellow, with the phosphate group highlighted in red.

To probe Pma1-lipid interactions in a native-like bilayer, we ran coarse-grained MD simulations of Pma1 in a complex bilayer of different lipid types occurring in *N. crassa* (fig. S5A and table S6) (*13*, *24*–*26*). To prevent any positional bias of lipids placed inside the ring, one monomer was omitted from the hexamer to allow a free lipid exchange during the simulations. Fractional interaction times of each lipid at the Pma1 surface suggest two putative binding sites at the monomer interface: site I in the inner membrane leaflet (involving M1, M3, and M10′) and site II in the outer membrane leaflet (between M4 and M10′) (Fig. 5A), with a preferred binding of phosphatidylserine (PS) and phosphatidylcholine (PC), respectively (Fig. 5B and fig. S6). Our cryo-EM maps do not clearly resolve any tightly bound lipids, probably due to a dynamic and inhomogeneous nature of lipid binding. Lipid-mediated hexamer contacts are in line with the observations that hexamer formation in vivo depends on an intact lipid biosynthesis pathway (*11*–*13*). In addition, the hexamer is sensitive to treatment with detergents such as octyl glucose neopentyl glycol (OGNG) in vitro (fig. S7). In our coarse-grained simulations, both sites preferably interact with the double-unsaturated 16:2/18:2 species, as compared to the monounsaturated 16:0/18:2 (Fig. 5B and figs. S6, S8, and S9). A local lipid density analysis shows that the accumulation of 16:2/18:2 PS (DIPS) at site I is accompanied by an extended clustering of this lipid in the vicinity (Fig. 5C). 16:2/18:2 PC (DIPC) is the only other lipid that also accumulates at the interface (at site II), albeit to a lesser extent than DIPS, and has an otherwise even distribution around the protein (Fig. 5C and fig. S9). Accumulation could be driven either by localized clustering of lipids with unsaturated tails, which would explain the presence of both DIPS and DIPC, or instead by clustering of the acidic PS head groups, which has been observed previously (*27*). A preferential occupation of site I with DIPS is in line with the fact that ATPase activity of detergent-purified Pma1 in vitro depends on the addition of acidic phospholipids (*14*). A very recent study also found an approximate threefold enrichment of PS in styrene-maleic acid (SMA)–solubilized Pma1 compared to the average plasma membrane composition (*26*).

The simulations further suggest that the Pma1 protein has a considerable impact on the local membrane structure: Near the M1-M4 bundle, the inner membrane leaflet is locally thinned, a “dip” that is most pronounced near the unwound region of M4 and along the outside-facing surfaces of M6 (the helix containing the proton-accepting Asp^730^) and M9 (Fig. 5D and fig. S10). Opposite this dip, on the inside of the ring, the cytosolic membrane surface bulges upward toward the P domain, probably attracted by the partial positive charge at the N-terminal end of M3 and by Lys^749^ in the L6-L7 loop. Both the “dip” and the “bulge” are also evident from the cryo-EM map of the micelle surrounding the Pma1 hexamer particles [likely consisting of a mixture of β-dodecyl-maltopyranoside (DDM) with residual membrane lipids] (Fig. 5E). Such a “chute”-like arrangement of negatively charged lipid headgroups near the mouth (also negatively charged; see Fig. 2C) of the ion entry funnel could facilitate the attraction of protons from the cytosol into the binding site. In support of this hypothesis, the simulations show an increased local density of Na^+^ ions from the solvent in this region (Fig. 5F).

The extracytosolic leaflet plug inside the hexamer is shifted upward in the simulations—an effect of unknown cause that has also been observed by atomic force microscopy inside the rotor rings of ATP synthase (*28*). This effect is, however, not evident from the micelle shape, in contrast to the cytosolic leaflet deformations. Together, the simulations suggest an approximate 10-Å decrease in membrane thickness (from ~37 to ~27 Å) in the region where the protein is inserted and a local thickening to ~54 Å in the membrane region below the P domain (Fig. 5D and fig. S10).

### Model for proton pumping and autoinhibition

To develop a model for the proton pumping cycle of Pma1 (fig. S11), we generated a series of homology models based on SERCA in the transition of phosphorylation (*E*1~P), open-to-outside (*E*2P), transition of dephosphorylation (*E*2~P), and dephosphorylated (*E*2) states. The transition state of phosphorylation, *E*1~P, is linked to ion occlusion within the binding site (*30*, *31*). In this state, Asp^730^ moves away from Asn^154^, implying a break of the suggested hydrogen bond (Fig. 3B). Nevertheless, the predicted p*K*_a_ (where *K*_a_ is the acid dissociation constant) value of Asp^730^ in this state (7.2) (*32*, *33*) suggests that it is protonated, likely because it is buried inside the hydrophobic core of the protein. Two carbonyl oxygens on the backbones of Ile^332^ and Ala^729^ may act as stabilizing H-bond acceptors. Arg^695^, the conserved residue also suggested to be involved in proton repulsion in *E*2, takes a position that shields Asp^730^ from the cytosolic side as the entry channel closes and possibly prevents the acquired proton from escaping back to the cytosol.

In the open-to-outside *E*2P homology model, Asp^730^ lies at the inner end of the exit funnel, which is lined by predominantly hydrophobic side chains, except for Asp^143^ approximately halfway down the funnel. Both His^701^ and Arg^695^ are in the vicinity of Asp^730^, and the Arg^695^ side chain can freely rotate into bonding distance (fig. S12). It could hence prevent reprotonation of Asp^730^ before the exit pathway is fully closed, working as an in-built counterion to neutralize Asp^730^ during the *E*2→*E*1 transition, as suggested for AHA2 (*16*, *22*). Functional studies suggest that Pma1 has a very low affinity for inorganic phosphate or phosphate mimics such as MgF*_x_*^−^ (*18*), indicating a rapid release and thus also a short lifetime of the extracellular proton exit gate. There is a conspicuous cluster of negatively charged residues Glu^139^, Asp^140^ (L1-L2), Asp^143^ (M2), Glu^324^ (M4), and Glu^720^ (M6) at the extracellular end of the funnel (fig. S12), which would facilitate proton extrusion.

Autoinhibition of Pma1 by the R domain has been postulated to function independent of the oligomeric state via a *cis*-acting immobilization of the cytosolic domains. This is supported by the fact that an excess of free R peptide counteracts autoinhibition (*10*). In our structure, the R helix interacts exclusively with the P domain, which is expected to move relatively little during proton pumping. All our (monomeric) modeled conformations can accommodate the R helix in this position without any clashes, which is in line with the observation that binding of free R peptide does not interfere with activity. However, the distance between the C terminus of M10 and the N terminus of the R helix increases from 30 Å in the *E*1 structure to 41 Å in the *E*2 homology model (fig. S13A), supporting the “clamping” model of autoinhibition, as the 16-residue linker between M10 and the R helix might restrain the *E*1→*E*2 transition.

In *Saccharomyces cerevisiae*, release of the autoinhibition upon glucose sensing has been linked to phosphorylation of Ser^899^ (Ser^901^ in *N. crassa*) by Ptk2 (*34*) and tandem phosphorylation of Ser^911^/Thr^912^ (Ser^913^/Thr^914^ in *N. crassa*) by an unknown kinase (*35*). Ser^899^-P leads to an increase in *K*_m_ (Michaelis constant), and Ser^911^-P/Thr^912^-P to an increase in *V*_max_ (*9*, *24*–*36*). Notably, neither of these residues is located at the binding interface of the *cis*-acting R domain (Fig. 4). A release of the R domain from the P domain in a monomeric context would therefore need to involve phosphorylation-induced structural alterations within the R helix itself, leading to disruption of R-P interactions. While such a mechanism is not impossible, it seems unlikely, which would point toward a role for the hexamer as the physiologically relevant unit of Pma1.

### The role of the hexamer

**Fig. 6.**
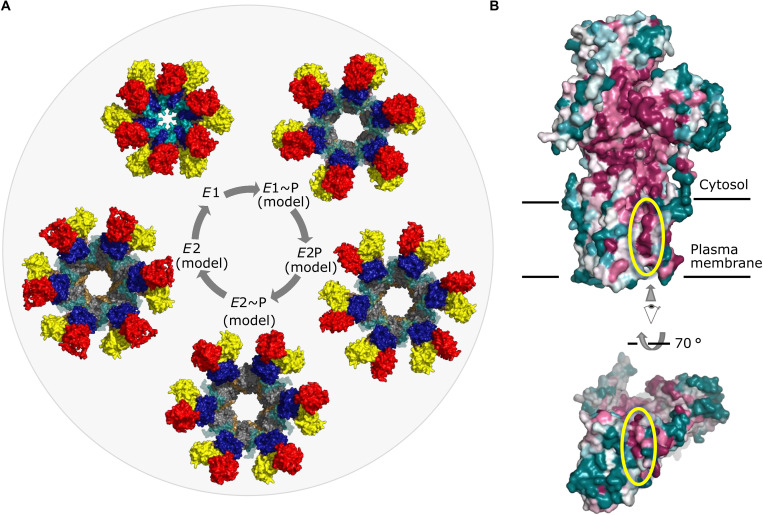
Hexameric models and surface conservation of Pma1. (**A**) Model of the catalytic cycle of hexameric Pma1. Homology models throughout the catalytic cycle were arranged into hexamers via alignment with M6-M10 of the *E*1 hexamer. The R domain was placed into the homology models in its relative position to the P domain as observed in *E*1. N domain, red; A domain, yellow; P domain, blue; R domain, cyan (transparent in homology models); M1-M2, pink; M3-M4, gold; and M5-M10, gray. The homology models were generated with SWISS-MODEL (*64*) based on a structure-based alignment and SERCA crystal structures with Protein Data Bank entries 1T5T (*E*1~P), 3B9B (*E*2P), 3N5K (*E*2~P), and 3NAL (*E*2). (**B**) Conservation of Pma1 against proton pumps from human- and plant-pathogenic fungi, calculated with ConSurf (*42*) and color-coded in a gradient from purple (conserved) to blue-green (variable). The yellow ellipse highlights a conserved ridge along a putative drug-binding groove in the M domain, which extends into the extracytosolic side of Pma1.

Originally, the hexamer has been suggested to serve as a space-saving form for efficient trafficking and for storage of large amounts of autoinhibited protein in the plasma membrane of starving cells (*10*, *37*). Upon glucose sensing, Pma1 is rapidly activated by phosphorylation of the R domain, which would release it from the P domain. There is no evidence to suggest that the hexamer needs to come apart for Pma1 to become fully active. While monomeric Pma1 is capable of pumping protons (*38*), Pma1 activity in vitro is mostly reported in a hexameric context, and the physiological importance of multimeric plasma membrane H^+^-ATPases is becoming more and more evident (*39*, *40*). Studies with purified plasma membranes have indicated the presence of either two cooperative ATP-binding sites or an even larger number of sites with a lower degree of cooperativity (*41*). In addition, the stoichiometry of DCCD inhibition suggests that binding of one molecule of DCCD is sufficient to block ATP hydrolysis in ~2.5 molecules of Pma1 (*19*), suggesting that the physiological unit of Pma1 is multimeric. Hexameric assemblies of the homology models show that there is sufficient space to accommodate the expected cytosolic domain rearrangements throughout the catalytic cycle, without any clashes (Fig. 6A). The models further suggest either that the hexameric ring would need to expand slightly to accommodate the opening of the extracellular pathway or that Pma1 does not open up as widely as SERCA, which served as a structural template. If this is the case, it may reflect the smaller size of the transported ion and the need to ensure quick closure upon proton release to prevent backflow at high membrane potentials.

We investigated all possible combinations of neighboring conformations, assuming an untethered R domain (see below). None of the 20 combinations clash, indicating that there is no cooperative domain movement as none of the catalytic states of one monomer affects that of its neighbors.

### Autoinhibition and activation in the hexamer

Comparing the autoinhibited hexamer structure with the homology-modeled active state in the process of phosphoryl transfer, the domain packing of the autoinhibited state is tighter. Accordingly, the accessible surface area in the autoinhibited hexamer is at a minimum (213,100 Å^2^), and all homology-modeled hexamers have substantially increased outer diameters compared to the 167 Å of the autoinhibited form (Fig. 6A and fig. S13B), suggesting that the early electron micrographs of dense Pma1 arrays [with estimated diameter of 150 Å (*8*)] represent the autoinhibited form.

The R domains are closely sandwiched between adjacent P domains, forming an extensive interaction network (Figs. 1B and 4). R domains of adjacent monomers interact directly: Ser^892^ and Pro^893^ interact with Leu^902^ of the adjacent R helix, thereby linking all R domains within the hexamer. During pumping, the P domains would need to increase their mutual distance, breaking the R domain–mediated cross-link. Hexamer contacts could hence augment the intramolecular clamping effect of the R domain. Furthermore, in four of the possible neighboring combinations within the hexamer (*E*2:*E*1, *E*2:*E*1~P, *E*2P:*E*1, and *E*2~P:*E*1), the R helix cannot be accommodated in the position observed in the *E*1 state. Together, this suggests that either the monomers within the hexamer must move in a coordinated manner or, more likely, the R domain is displaced from the P domain upon Pma1 activation.

Phosphorylation of Ser^913^/Thr^914^ would disturb the contact between the R domain of one monomer and the adjacent P domain. In contrast, Ser^901^ points into the center of the hexameric ring, and hence, its phosphorylation by Ptk2 is unlikely to affect the intermolecular contact directly. A single molecule of Ptk2 can fit into the center of the Pma1 hexamer and could lead initially to the phosphorylation of Ser^901^, followed by ATP binding and phosphoryl transfer in just one monomer. Domain rearrangements of this first monomer would then lead to an enhanced accessibility of the two neighboring monomers’ R domains, perpetuating the activation through the hexamer. Notably, the side chain of Ser^901^ is situated only 5.4 Å away from Arg^900^ of one adjacent monomer, allowing an R-R contact upon phosphorylation that could eventually sequester together all six untethered R domains. This could aid the activation process and stabilize the activated state. A similar interaction between inhibitory domains has been suggested in a recent cross-linking study on the plant proton pump AHA2 (*40*).

### Pma1 as a drug target

A main incentive of determining a fungal H^+^-ATPase structure is to provide the basis for developing small-molecule inhibitors as novel antifungals. To explore the amenability of Pma1 to specific small-molecule drugs, we aligned the *N. crassa* Pma1 primary sequence to that of the most important human- and plant-pathogenic fungi (table S6) and plotted the conservation score on the protein surface using the ConSurf Server (Fig. 6B) (*42*). Focusing on the transmembrane and extracellular regions, which would be least conserved relative to human P-type ATPases and most accessible by drugs, we identified a putative drug-binding pocket in a deep groove between M1, M3, and M4, along a ridge of highly conserved residues, and extending to the extracellular surface (Fig. 6B). To probe this groove’s suitability for drug binding, we docked a set of nine recently reported novel Pma1 inhibitors [tetrahydrocarbazoles (THCAs)] (*43*) into the Pma1 structure, using the entire transmembrane and extracellular region of one monomer as search space (fig. S14). Out of the total 162 calculated docking poses, 21 poses both had a calculated binding affinity of <−8.5 kcal/mol and had not been docked into the hexamer interface. Encouragingly, 18 of these 21 docking poses were located in the conserved groove described above (fig. S14).

## DISCUSSION

The Pma1 structure reveals that core elements of all known proton-pumping proteins are conserved within the Pma1 monomer: (i) a central proton acceptor/donor (Asp^730^), (ii) a positively charged residue to control p*K*_a_ changes of the proton acceptor/donor (Arg^695^), and (iii) bound water molecules to facilitate rapid proton transport by Grotthus shuttling (*44*).

The properties of the Pma1 hexamer reveal further important insights: The dependence of Pma1 activity on anionic lipids can be rationalized by a preferential interaction site for these lipids at the monomer interface. The function of the anionic lipids is likely not limited to the mediation of hexamer contacts but could also be involved in the attraction of protons into the acceptor site. This is in line with our finding of a region of local membrane deformation and a concomitant clustering of cations. Local attractions between the lipid bilayer surface and the protein may also facilitate or accelerate conformational changes in the protein. Mutual adaptations between protein and membrane, as well as membrane deformations assisting cytosolic ion entry have already been suggested for SERCA (*17*, *29*) and might be a common feature of P-type ATPase function.

We find no structural basis for cooperativity between the monomers in the hexamer, but the structure implies that autoinhibition is enhanced by—if not dependent on—a hexamer context. Activation by phosphorylation must occur sequentially because of steric hindrance and is probably accelerated by mutual sequestering of the released R domains. Further investigations on the structure-function relationship of glucose-activated Pma1, as well as on mutants unable to hexamerize, will clarify these issues. Furthermore, the putative inhibitor-binding groove suggested by our docking studies will need to be confirmed experimentally. Notably, most of the conserved residues around this groove are also conserved in the related plant proton pump (table S5), making this site a potentially more promising target for therapeutic applications than for plant protection.

## MATERIALS AND METHODS

### Plasma membrane preparation

Purification of Pma1 was carried out from a native source, using a cell wall–less *N. crassa* strain [Fungal Genetics Stock Center (FGSC), strain #4761]. Cells were grown for 24 hours at 30°C and 185 rpm (Brunswick Innova 44) in Vogel’s medium supplemented with 2% (w/v) mannitol, 0.75% (w/v) yeast extract (Difco), and 0.75% (w/v) nutrient broth (Difco) using 2.5-liter Ultra Yield flasks with AirOtop lids. Plasma membrane preparation was carried out as described previously (*46*) with some adjustments to the original protocol. All centrifugation steps were conducted at 4°C; cells and membranes were kept on ice unless stated otherwise. The cells were harvested by centrifugation (15 min at 700*g*) and washed four times with ice-cold buffer A [50 mM tris (pH 7.5), 10 mM MgSO_4_, and 250 mM mannitol] (15 min, 700*g*) before they were agglutinated with concanavalin A (0.5 mg/ml) in buffer A for 10 min at room temperature (RT) and another 10 min on ice. Agglutinated cells were pelleted for 6 min at 200*g* and washed once with ice-cold buffer A. Lysis was obtained by homogenizing the cells in ice-cold lysis buffer [10 mM tris (pH 7.5), 1 mM MgCl_2_, 1 mM CaCl_2_, chymostatin (1.5 μg/ml), and deoxyribonuclease (DNase) I (1 μg/ml)] containing 1.2% sodium deoxycholate (DOC) using a 50-cm^3^ glass homogenizer. After centrifugation (30 min, 14,000*g*), the membrane pellet was first washed with buffer B [10 mM tris (pH 7.5) and chymostatin (1.5 μg/ml)] containing 0.6% DOC and then with DOC-free buffer B. Dissociation of concanavalin A from the plasma membrane was achieved by incubation with 0.5 M α-methylmannoside in buffer B for 5 min at 30°C. The dissociated membranes were diluted with ice-cold buffer B, pelleted (30 min, 14,000*g*), and washed once with ice-cold buffer B. After resuspension in ice-cold buffer B, membranes were flash-frozen in liquid nitrogen and stored at −80°C until protein purification.

### Detergent solubilization and protein purification

Membranes were thawed in a RT water bath, spun down (30 min, 17,700*g*, 4°C), and washed twice [20 mM Hepes (pH 7.2), 0.5 M NaCl, and 1 mM phenylmethylsulfonyl fluoride]. Solubilization was carried out for 1 hour at 12° to 19°C with 2.25 mg/ml of the detergent DDM in Pma1 solubilization buffer [10 mM tris (pH 7.5), 150 mM NaCl, 1 mM Na_2_-ATP, 5 mM MgSO_4_, 0.1 mM Na_3_VO_4_, and chymostatin (2 μg/ml)] using a total membrane protein concentration of 2 mg/ml determined by bicinchoninic acid assay. All further purification and centrifugation steps were performed at 4°C. Unsolubilized membranes were removed by centrifugation (50 min, 17,700*g*), and the supernatant was filtered through a 0.45-μm nylon membrane.

The protein solution was concentrated by pressure dialysis with a stirred cell (Amicon) and a 300-kDa molecular weight cutoff (MWCO) filter membrane, diluted to a NaCl concentration of ~40 to 45 mM [50 mM MES/tris (pH 7), 5 mM MgSO_4_, glycerol (200 g/liter), 1 mM dithiothreitol (DTT), and 2 mM EDTA], and loaded onto a Q HP anion exchange (AEX) column (GE Healthcare) equilibrated with dilution buffer containing DDM (0.1 mg/ml). The AEX column was washed with 20 column volumes (CV) of wash buffer [50 mM MES/tris (pH 7), 20 mM KCl, 5 mM MgSO_4_, 1 mM DTT, 2 mM EDTA, DDM (0.15 mg/ml), and chymostatin (2 μg/ml)] and eluted with a linear gradient to 500 mM KCl over 20 CV. Peak elution fractions were pooled, diluted 1:10 [50 mM MES/tris (pH 6), 5 mM MgSO_4_, 2 mM EDTA, 1 mM DTT, chymostatin (2 μg/ml), and DDM (0.15 mg/ml)], and concentrated to a volume of 1 to 2 ml with Amicon Ultra centrifugal filters (100-kDa MWCO).

The concentrated sample was loaded onto a glycerol density gradient from 20 to 40% (w/v) (5% steps) and ultracentrifuged for 16 hours at 34,400*g* (Optima L-100 XP, Ti70 rotor, acceleration 2, no brake). The gradient was harvested in 1-ml fractions from bottom to top using a peristaltic pump with high-performance liquid chromatography tubing (inside diameter of 0.15 mm). The purest fractions were pooled, diluted 1:10 in glycerol-free buffer, and concentrated to a volume of 0.5 to 1 ml. The remaining Pma1-containing fractions were also pooled, diluted 1:5, and concentrated to a volume of 0.5 to 1 ml. The concentrated samples were loaded onto two separate glycerol density gradients from 20 to 45% (w/v) (5% steps), ultracentrifuged, and harvested as described above. The purest fractions of both gradients were pooled, concentrated, and loaded onto a Superose 6 Increase 10/300 (GE Healthcare) size exclusion chromatography (SEC) column equilibrated with SEC buffer [50 mM MES/tris (pH 6.5), glycerol (200 g/liter), 50 mM KCl, 5 mM MgSO_4_, 2 mM EDTA, 1 mM DTT, chymostatin (2 μg/ml), and DDM (0.15 mg/ml)]. Peak fractions were evaluated by silver stain SDS–polyacrylamide gel electrophoresis and negative-stain EM.

### Negative-stain EM

Samples were diluted to 0.015 mg/ml [determined with NanoDrop One and Abs 0.1% = 1.044 calculated by ProtParam (*47*)] with glycerol-free SEC buffer. Carbon-coated 400-mesh copper grids were glow-discharged (15 mA, 45 s), incubated with 3 μl of sample for 30 s, and stained twice for 15 s with 3 μl of 2% uranyl formate. Blotting after each incubation step was performed at a 45° angle with Whatman filter paper (#1). The grids were imaged with 120-kV Tecnai Spirit (Thermo Fisher Scientific) at ×52,000 magnification.

### Cryo-EM

Suitable SEC elution fractions (see fig. S5) were pooled and concentrated to 40 μl at 3 mg/ml with Amicon Ultra centrifugal filters (100-kDa MWCO). The buffer was exchanged to cryo-EM buffer [30 mM MES (pH 6.5), 1 mM MgSO_4_, 1 mM DTT, chymostatin (2 μg/ml), and DDM (0.15 mg/ml)] with a 0.5-ml Zeba Spin column (7 kDa) following the manufacturer’s protocol. The eluted sample was concentrated to 3.7 mg/ml and incubated for 2 hours on ice with 1 mM ADP and 0.6 mM AlF_4_ at a final protein concentration of 2 mg/ml (~20 μM). Cryo-EM grids were prepared by applying 3 μl of sample to twice glow-discharged (15 mA, 45 s) C-flat R2/2 400-mesh copper grids, which were blotted for 11 s (10°C, 70% humidity, S&S 595 filter paper) and plunge-frozen in liquid ethane using Vitrobot Mark IV (Thermo Fisher Scientific). Cryo-EM grids were stored in liquid nitrogen until data were collected with 300-kV Titan Krios G3i (Thermo Fisher Scientific) equipped with a K3 direct electron detector (Gatan). Micrographs (2780) were recorded automatically in EPU (Thermo Fisher Scientific) in counting mode at 105,000× magnification with a calibrated pixel size of 0.837 Å. The defocus range was set from −1.3 to −2.5 μm, and each micrograph was dose-fractionated to 50 frames with a total exposure time of 3 s, resulting in a total dose of ~42 *e*^−^/Å^2^ (see table S1).

### Cryo-EM image analysis

CryoSPARC (*48*) was used for particle picking and selection, while all further refinement steps were performed in Relion (*49*–*51*). After patch-based motion correction and contrast transfer function (CTF) estimation in cryoSPARC, particles were picked with the blob picker (180 Å diameter) and manually curated, resulting in 483,742 particles. Four iterative rounds of 2D classification led to 95,525 particles, which were used for an ab initio reconstruction with three classes, resulting in two protein classes with 81,009 particles and one junk class. A homogeneous refinement gave an initial map of hexameric Pma1 at 3.6-Å resolution (using C1 symmetry). The raw micrographs were then imported to Relion 3.0 and motion-corrected with MotionCor2 (*52*). CTF estimation was performed using Gctf (*53*) on non–dose-weighted aligned micrographs, and the cryoSPARC particles were reextracted using the coordinates from the homogeneous refinement. The following processing steps were performed in Relion 3.1 beta. An initial 3D refinement applying C6 symmetry resulted in a 3.79-Å map. CTF refinement and anisotropic magnification estimation (*54*) improved the resolution to 3.53 Å, Bayesian polishing (*55*) to 3.42 Å (map H1), and another CTF refinement to 3.28 Å (map H2). The map was of high quality for the M and P domains and part of the R domain, but the A and N domains were less well resolved. Then, the hexamer particles from the map H2 were subjected to a 3D classification focused on the most rigid part of the hexamer (M and P domains). The remaining 59,511 particles (73.3%) were 3D refined (with a mask around the whole hexamer) to 3.28-Å resolution (map H3). The map was further postprocessed with DeepEMhancer (*56*) (map H3-DE) and density-modified (model-free) using phenix.resolve_cryo_em (*57*) (H3-DM) to aid interpretation and visualization.

To further improve the resolution of the cytosolic A and N domains, the particles of map H2 were symmetry-expanded and a focused 3D classification was performed (*50*) using a monomer mask. The best monomer class appeared in 59.9% (293,999) of the expanded hexamer particles, which were then used for 3D refinement applying C1 symmetry and only local angular searches, to prevent larger particle rotations (*50*). The refinement was started with a mask encompassing the whole hexamer to prevent particle misalignment. From a later iteration, the refinement was continued using a mask that again covered the same monomer on which the 3D classification had been focused. Focused refinement improved the map resolution to 3.21 Å (map M) and improved the signal for the cytosolic A and N domains, allowing model building. Map M was further postprocessed with DeepEMhancer (map M-DE) and density-modified (model-free) with phenix.resolve_cryo_em (*57*) (map M-DM), which further aided model building, evaluation, and map visualization. A summary of the refinement process is depicted in fig. S2A.

### Model building and refinement

The original and postprocessed versions of monomer map M were used for de novo model building in Coot (*58*), assisted by homology models (see below) and the Namdinator server (*59*). Amino acid residue assignment was based on clearly defined densities of bulky residues (Phe, Trp, Tyr, and Arg), the MgADP ligand, and K^+^ ion that were added to the model based on the crystal structures of the plant proton pump (*16*) and SERCA (*17*, *30*). The final model was refined using phenix.real_space_refine (*60*) and the density-modified monomer map M-DM. To generate the hexameric model from the refined monomer, C6 symmetry was applied to expand the monomer model into the density-modified hexamer map H3 using UCSF Chimera (*61*). The geometry of both models was validated using MolProbity (*62*) implemented in the Phenix Comprehensive validation tool (*63*) (see table S1). Final models of the monomer and hexamer were submitted to the Protein Data Bank, PDB ID 7NXF and 7NY1, respectively.

### Homology modeling and internal cavities

Homology models were generated with SWISS-MODEL (*64*) based on a structure-based alignment (*65*) and the SERCA crystal structures 1T5T (*E*1~P) (*30*), 3B9B (*E*2P) (*66*), 3N5K (*E*2~P) (*67*), and 3NAL (*E*2) (*68*). The SWISS-MODEL homology models were aligned to monomeric and hexameric Pma1 via M6-M10. Internal cavities of monomeric Pma1 and homology models were detected with HOLLOW (*69*).

### Inhibitor docking

The docking of a set of THCA Pma1 inhibitors (*43*) into the structure of the Pma1 monomer was done with AutoDock Vina (*70*). Nine THCA compounds were taken from a previous study (*43*), for which they had been built and energy-minimized in MAESTRO (Maestro, Schrödinger LLC, New York, NY, 2017) and prepared for docking in AutoDockTools, making all relevant bonds rotatable. Ligands were added in both their R and S configurations to a box encompassing the entire transmembrane domain of Pma1. Nine binding modes per configuration were analyzed, resulting in a total of 162 binding events of which 60 displayed affinities stronger than −8.5 kcal/mol. Thirty-nine binding events occurred at one of the monomer interfaces and were therefore excluded from further analysis. Three binding events took place within the proton entry funnel, and 18 within the exit funnel. The strongest binding, exceeding −9 kcal/mol, was observed for 10 events within the exit funnel. Residues involved in the putative binding of the respective THCA compounds (6S, 7S/R, and 8R) were identified using LigPlot+ (see fig. S14) (*71*).

### Coarse-grained MD simulations

The pentamer structure coordinates were converted to coarse-grained Martini representation using the martinize script (*72*). Their tertiary structures were constrained using the ElNeDyn elastic network between all chains (*73*) with a force constant of 500 kJ/mol per nm^2^ and a cutoff of 0.9 nm. The CG protein coordinates were then positioned in the center of a simulation box of size 23 nm by 23 nm by 23 nm, with its principal transmembrane axis aligned parallel to the *z* axis and embedded in a complex asymmetric membrane bilayer composed of 14 lipid species using the insane script (*74*). The membrane bilayer was built according to table S6. To investigate the role of lipid saturation, we also set up simulations of pentameric Pma1 in a 50% C16:0/18:2 PC (PIPC):50% DIPC symmetric membrane. NaCl (0.15 M) was added to the solvated system. The Martini coarse-grained force field version 2.2 (*72*) was used for protein and version 2.0 for lipids. All the simulations were performed using GROMACS 2019.1 (*75*). The systems were energy-minimized using the steepest descents method, equilibrated for 50 ns using 20-fs time steps in the isothermal-isobaric (NPT) ensemble at 323 K using the V-rescale thermostat (*76*) and at 1 bar using a semi-isotropic Berendsen barostat (*77*). Five production simulations were run to 10 μs using the Parrinello-Rahman barostat (*78*). Data were analyzed using VMD (*79*), GROMACS tools (*77*), and in-house scripts. A weighted atomic density for Na^+^ at each grid point was calculated using the VMD VolMap tool over 50 μs of simulation with default settings. Plots were made using MDAnalysis (*80*) and Microsoft Excel.

### Figure preparation

Figures were prepared using VMD (*79*), ChimeraX (*81*), and PyMOL (*82*). The OPM database (*45*) was used to estimate the membrane position for Fig. 2C. EM maps shown in figures are M-DE (Fig. 1A and fig. S2D), M-DM (fig. S4), H3 (fig. S2B), and H3-DE (Fig. 1B and fig. S2C).

## Notes

View/request a protocol for this paper from *Bio-protocol*.
