# Supplementary material for: Structure of the hexameric fungal plasma membrane proton pump in its autoinhibited state
Source: Sci Adv. 2021 Nov 10;7(46):eabj5255. doi: 10.1126/sciadv.abj5255 (PMC8580308; doi:10.1126/sciadv.abj5255)
Supplement: Supplementary file 1 — Figs. S1 to S14 Tables S1 to S6 [file sciadv.abj5255_sm.pdf]

Science Advances
AAAS

Supplementary Materials for

Structure of the hexameric fungal plasma membrane proton pump in its autoinhibited state

Sabine Heit, Maxwell M. G. Geurts, Bonnie J. Murphy, Robin A. Corey, Deryck J. Mills, Werner Kühlbrandt, Maike Bublitz*

*Corresponding author. Email: maike.bublitz@bioch.ox.ac.uk

Published 10 November 2021, *Sci. Adv.* **7**, eabj5255 (2021)
DOI: 10.1126/sciadv.abj5255

This PDF file includes:

Figs. S1 to S14
Tables S1 to S6

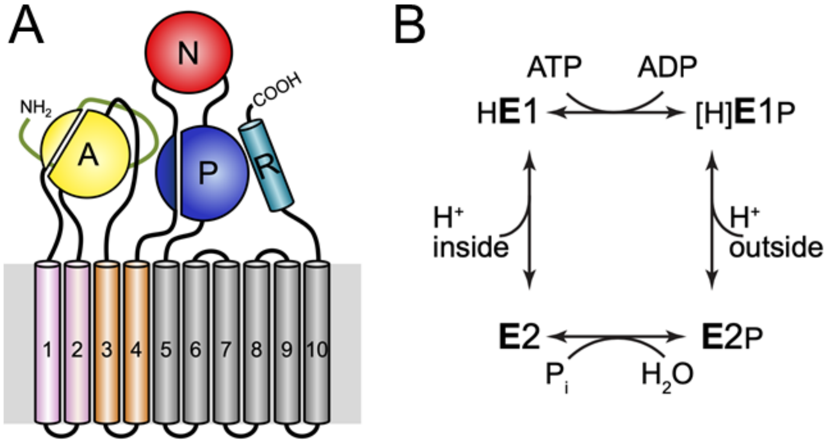

Fig. S1: Topology diagram and *E*1/*E*2 scheme of Pma1. (**A**) Overall Pma1 topology. Nucleotide-binding (N) domain red, actuator (A) domain yellow, phosphorylation (P) domain blue, regulatory (R) domain cyan, N-terminal extension (green), M1-2 pink, M3-4 gold, and M6-10 grey. (**B**) Canonical *E*1-*E*2 catalytic cycle for proton pumping by Pma1 with transient phosphorylation.

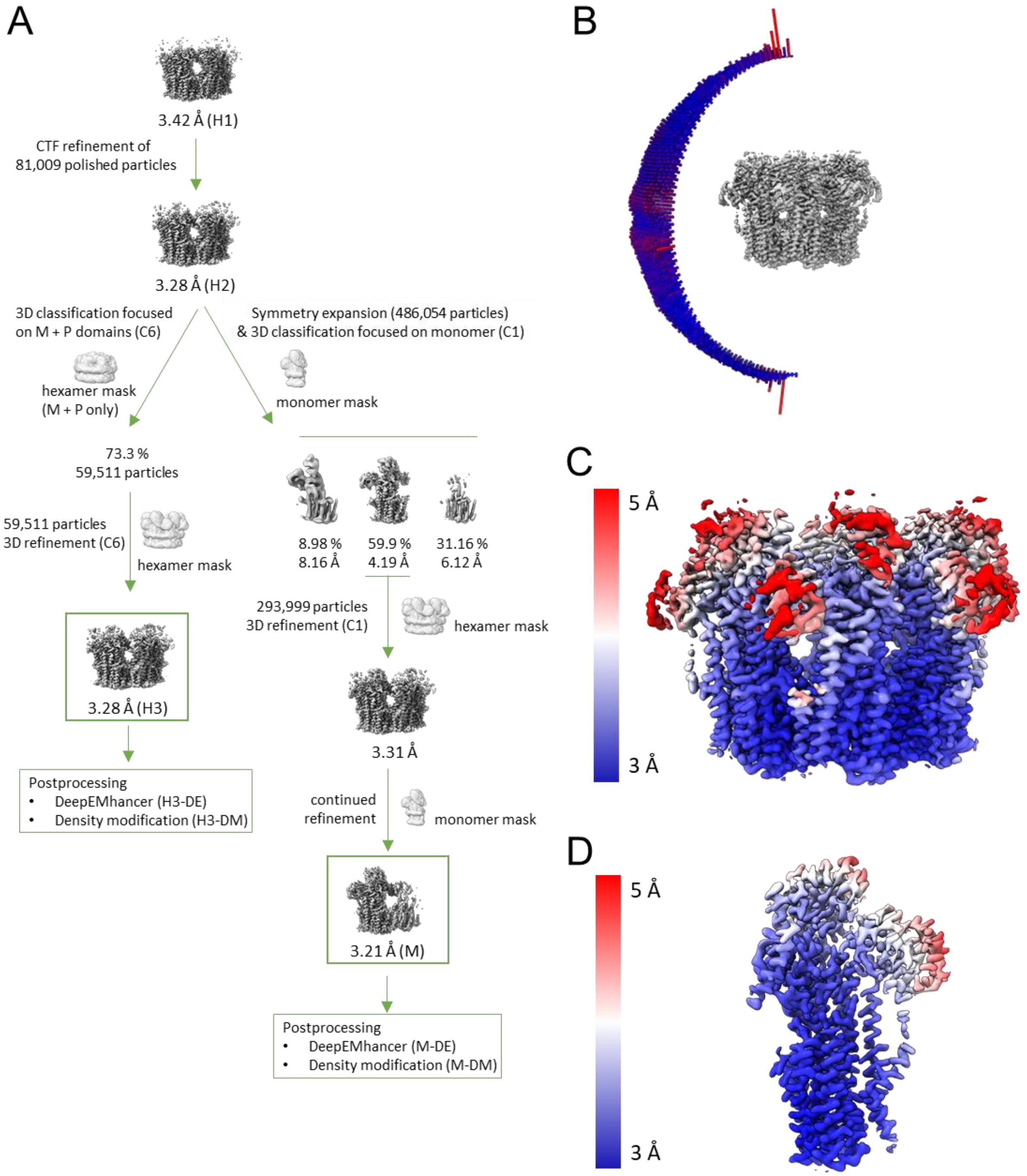

Fig. S2: Overview of the refinement of the Pma1 hexamer and monomer cryo-EM maps.
(**A**) Schematic representation of the refinement workflow resulting in the final hexamer map H3 and the final focused monomer map M. Masks for 3D classification and refinement are shown in white. (**B**) Angular distribution plot of all particles that contributed to the final hexamer map H3. The height of the bars is proportional to the number of particles in those views. (**C**) Local resolution of the final hexamer map H3 and (**D**) for the final monomer map M. Resolution estimates from Relion 3Drefine (*51*), maps shown are H3-DE and M-DE.

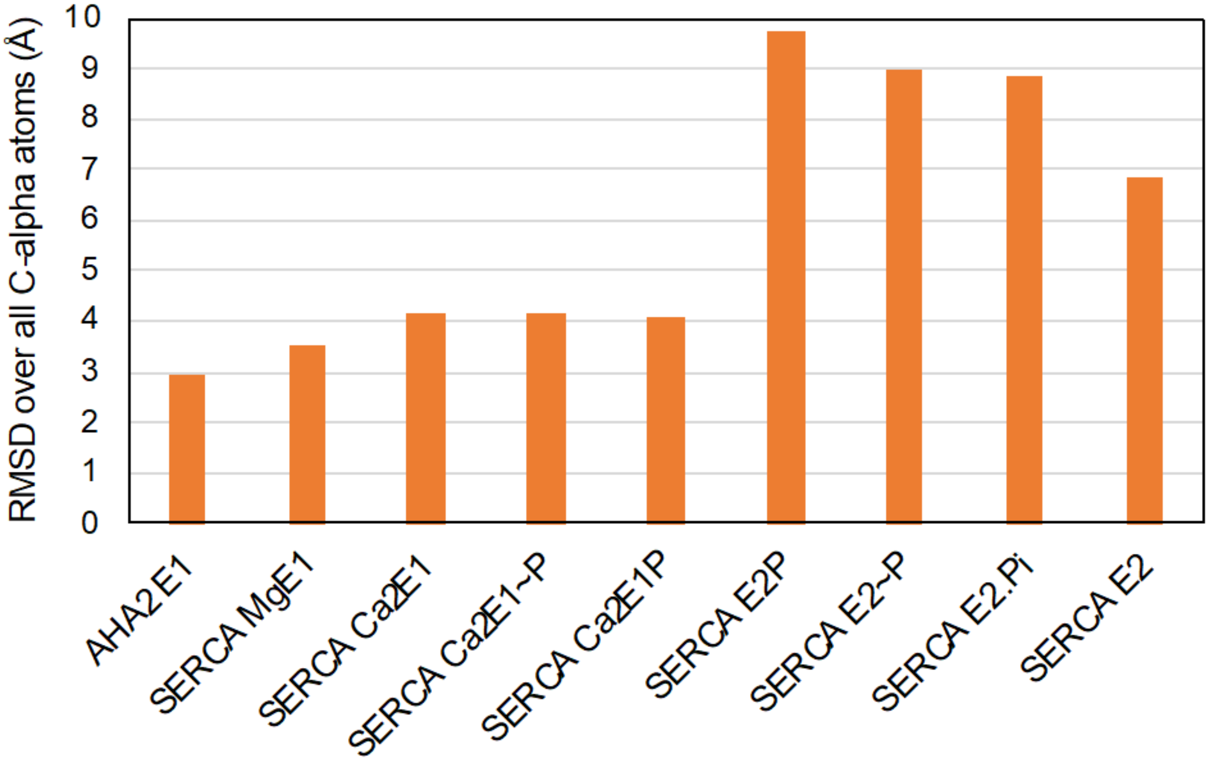

Fig. S3: Structural state comparison of Pma1 with related P-type ATPases. RMSD (root mean square deviation) calculated over all C-alpha atoms of Pma1 (*E*1) compared to the crystal structures of AHA2 (*E*1) and SERCA in different states along the catalytic cycle using PyMOL(*82*). PDB entries: 5KSD (AHA2 E1), 4HW1 (SERCA Mg*E*1), 3N8G (SERCA Ca2*E*1), 1T5T (SERCA Ca2*E*1~P), 3BA6 (SERCA Ca2*E*1P), 3B9B (SERCA *E*2P), 3N5K (SERCA *E*2~P), 1WPJ (SERCA *E*2.P_i), 3NAL (SERCA *E*2).

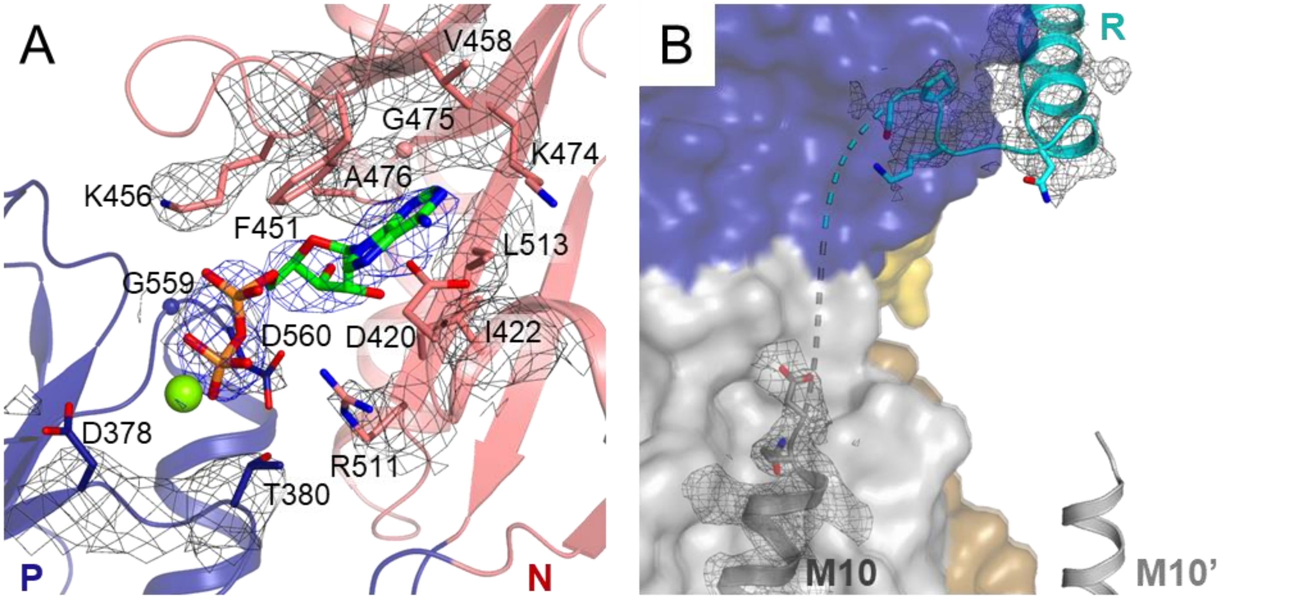

Fig. S4: Nucleotide-binding site and assignment of the R domain to its respective monomer.
(**A**) ADP is bound at the nucleotide-binding site between the P (blue) and N (light red) domains. ADP, residues involved in its coordination and Asp378 are shown as sticks (spheres for glycine) with C-atoms coloured in green (ADP) or according to their domain, a Mg^{2+} ion is shown as a light green sphere. The cryo-EM map (M-DM) is shown as blue or black mesh for MgADP and the protein, respectively, with a higher contour level for MgADP. Polar contacts are indicated by yellow dashes. (**B**) The distance from the helical part of the R domain to M10 and M10’ (shown as cartoon) is very similar, the assignment is based on a short extension at the N terminus of the R-helix that points towards M10. The cryo-EM map (M-DM) is shown as grey mesh, residues of the non-helical part of R and M10 are shown in stick representation.

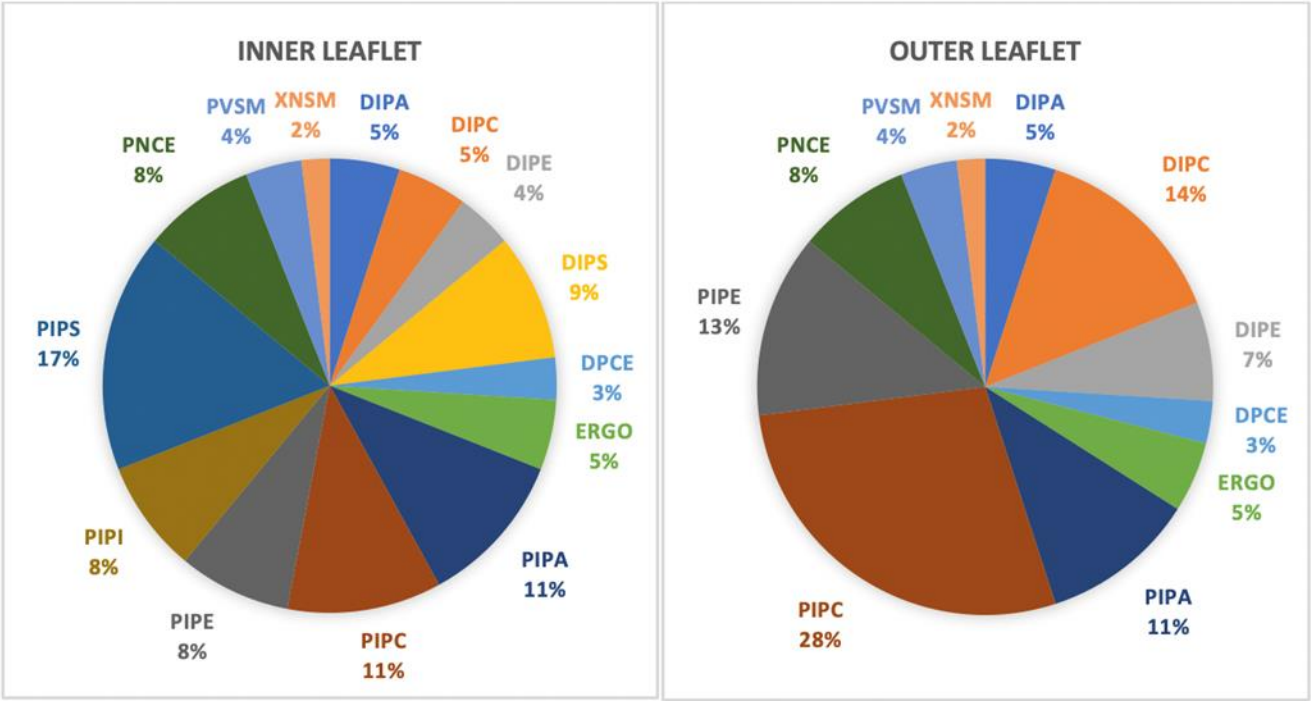

Fig. S5. Membrane lipid composition used in the coarse grained MD simulations of Pma1. For details on the lipids, refer to **Table S6.**

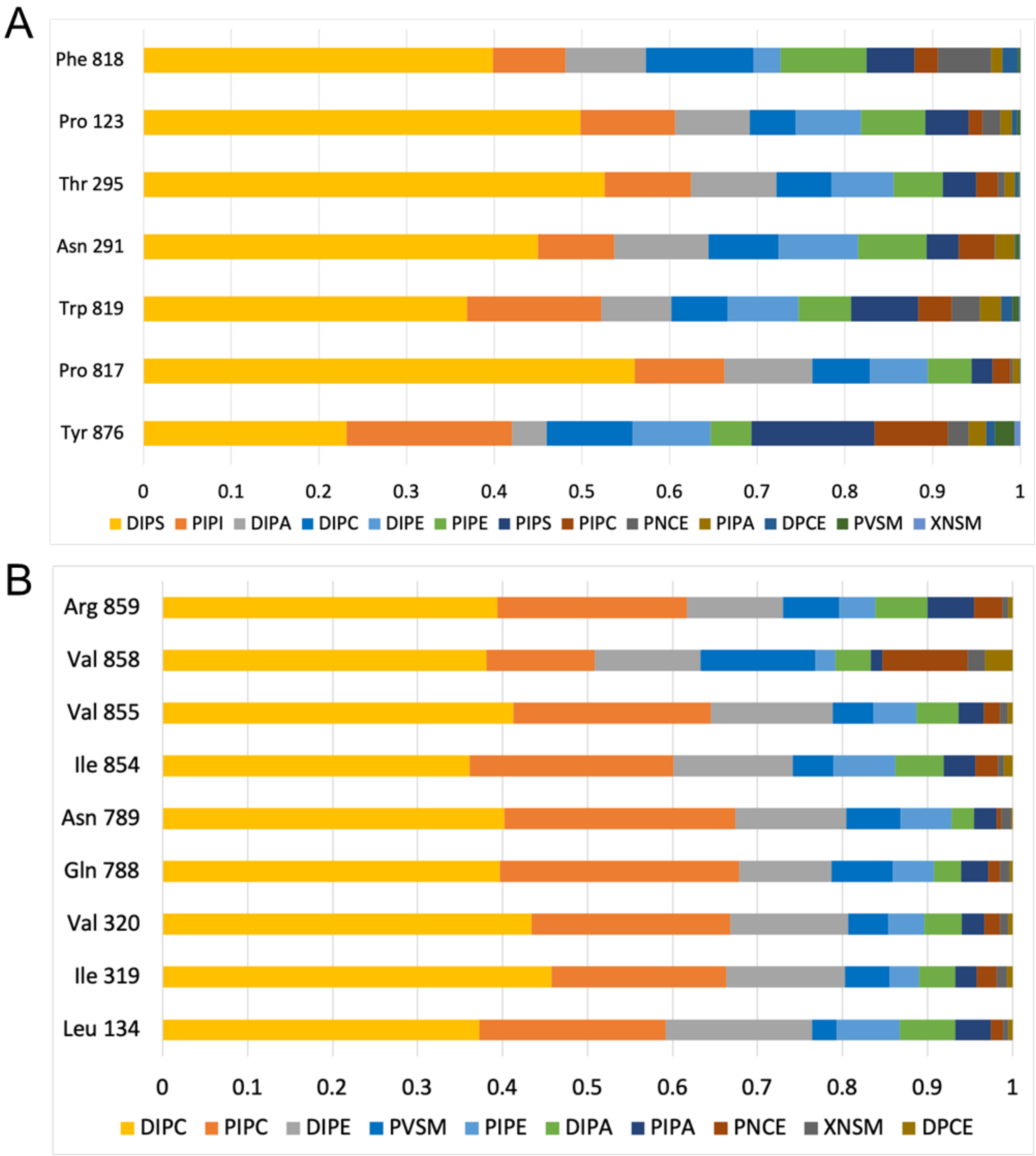

Fig. S6. Fractional interaction times of all lipids with Pma1 residues surrounding (**A**) site I and (**B**) site II, defined as the number of frames in which a lipid is within 0.6nm of a given residue. For details on the lipids, refer to **Table S6.**

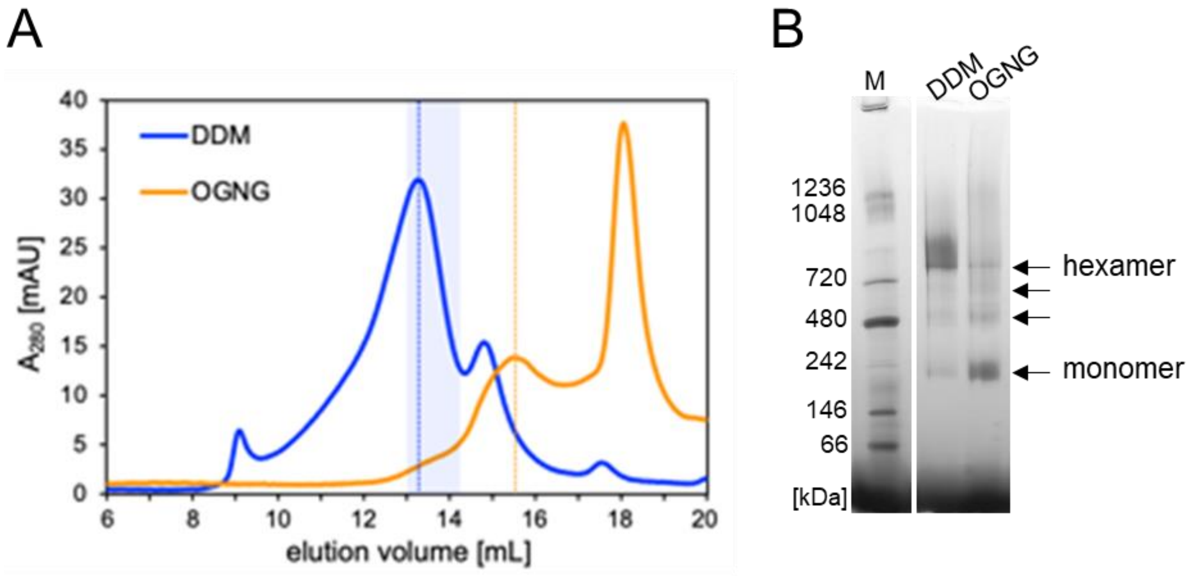

Fig. S7: Monomerisation of Pma1 with OGNG. (**A**) Size-exclusion chromatography of Pma1 in n-dodecyl-β-D-maltopyranoside (DDM) (blue; highlighted fractions used for cryo-EM) or octyl glucose neopentyl glycol (OGNG) (orange). SEC column: Superose 6 Increase 10/300 (GE Healthcare). (**B**) Native PAGE of Pma1 in DDM or OGNG (samples in 0.2 M ammonium acetate buffer for native MS). Unlabelled arrows indicate uncharacterised Pma1 oligomers (potentially dimers and tetramers). Protein ladder (M): NativeMark™ (Invitrogen), gel: 3-12% NativePAGE™ (Invitrogen).

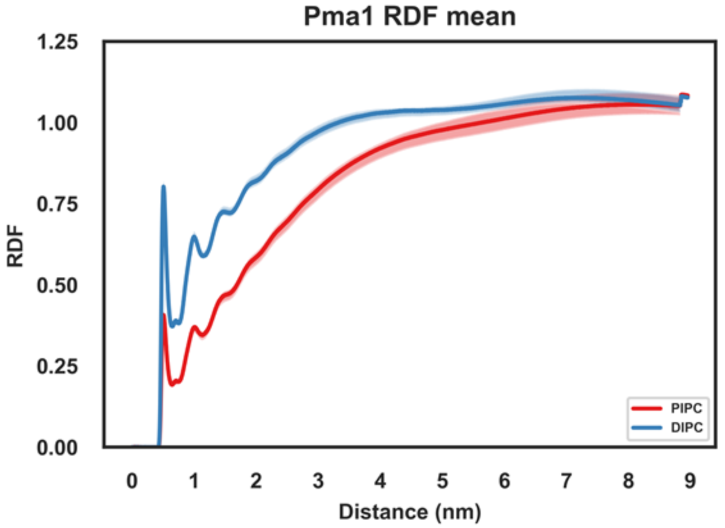

Fig. S8. **Radial distribution functions of PIPC and DIPC in the coarse grained simulation of a PIPC/DIPC-only membrane.** The distribution confirms a preferential binding of Pma1 to double-unsaturated lipids. For details on the lipids, refer to **Table S6.**

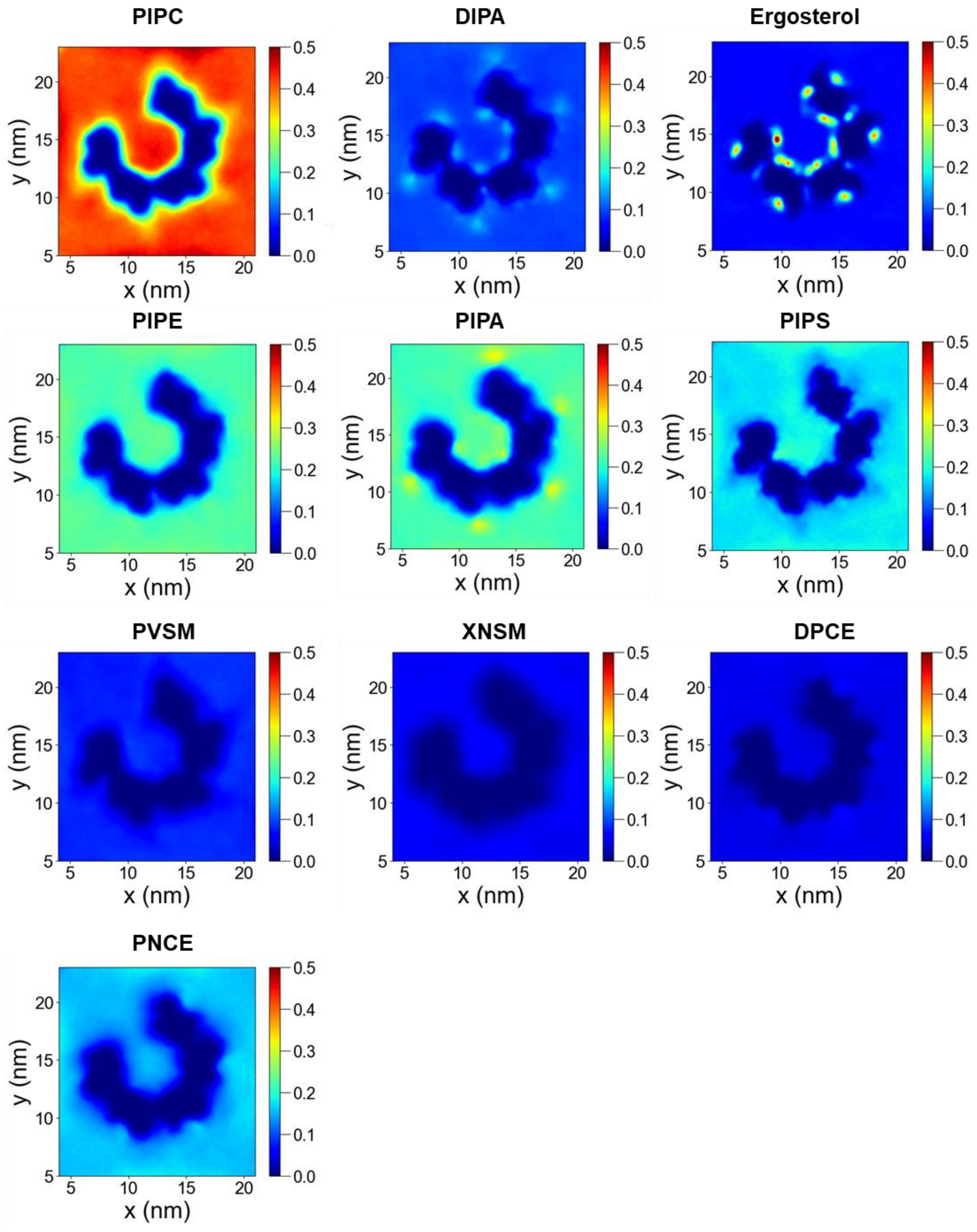

Fig. S9. Lipid density maps for PIPC, DIPA, Ergosterol, PIPE, PIPA, PIPS, PVSM, XNSM, DPCE, and PNCE. Values correspond to average numbers of molecules per nm3 and do not account for the respective membrane composition fraction. For details on the lipids, refer to **Table S6**.

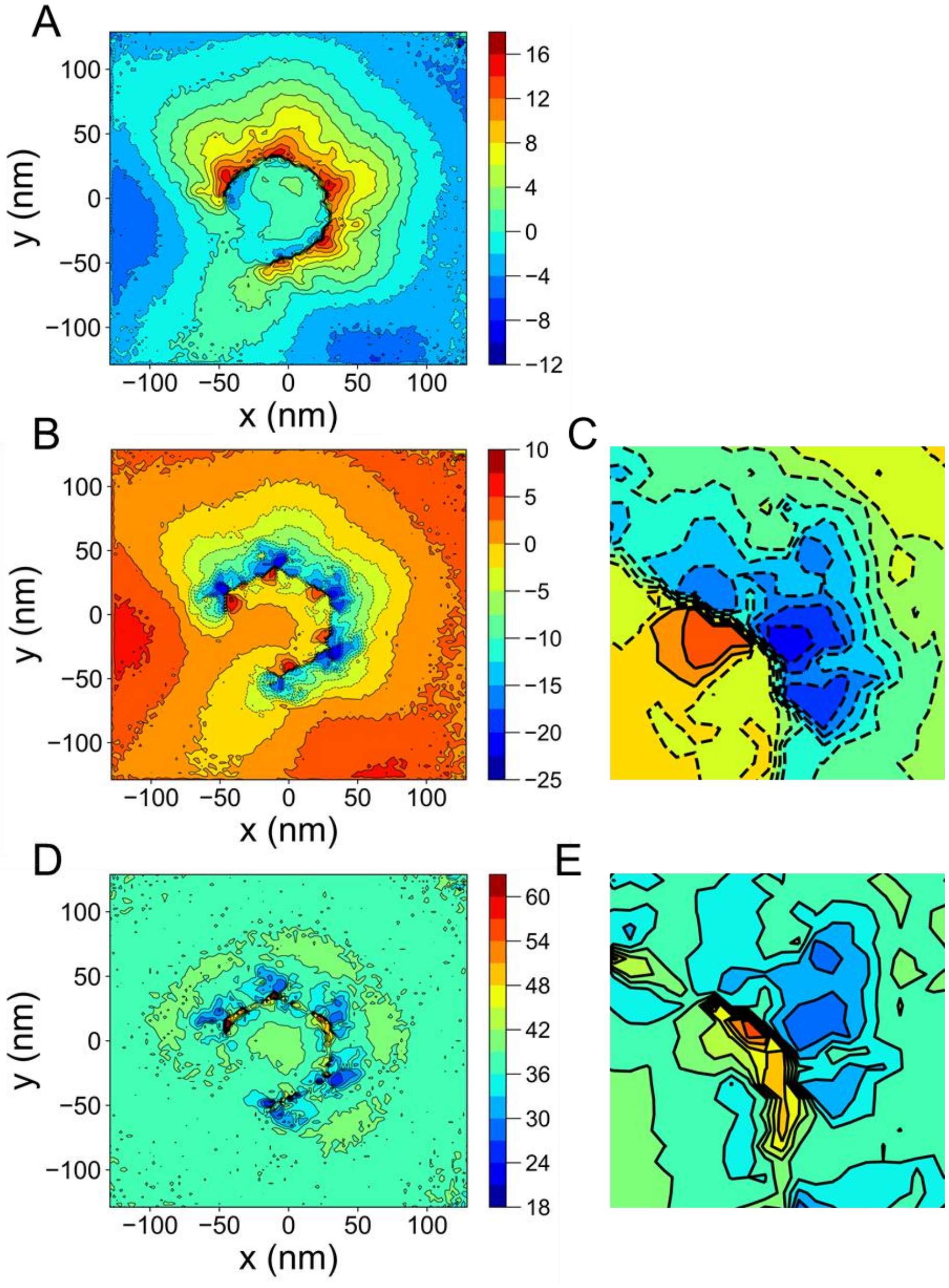

Fig. S10. Quantitative bilayer deformation analysis. Average z-height position of lipid headgroup-phosphates in (**A**) the outer leaflet, and (**B**) the inner leaflet. Values represent the z-height difference in Ångström relative to the value at coordinate -50, 110 (assumed to represent a membrane region unperturbed by protein or boundary effects). (**C**) Zoom of (B) on the region of one Pma1 monomer. (**D)** Average leaflet thickness between phosphates (inner leaflet minus outer leaflet) at each x, y coordinate. Scale is in Ångström. (**E**) Zoom of (D) on the region of one monomer.

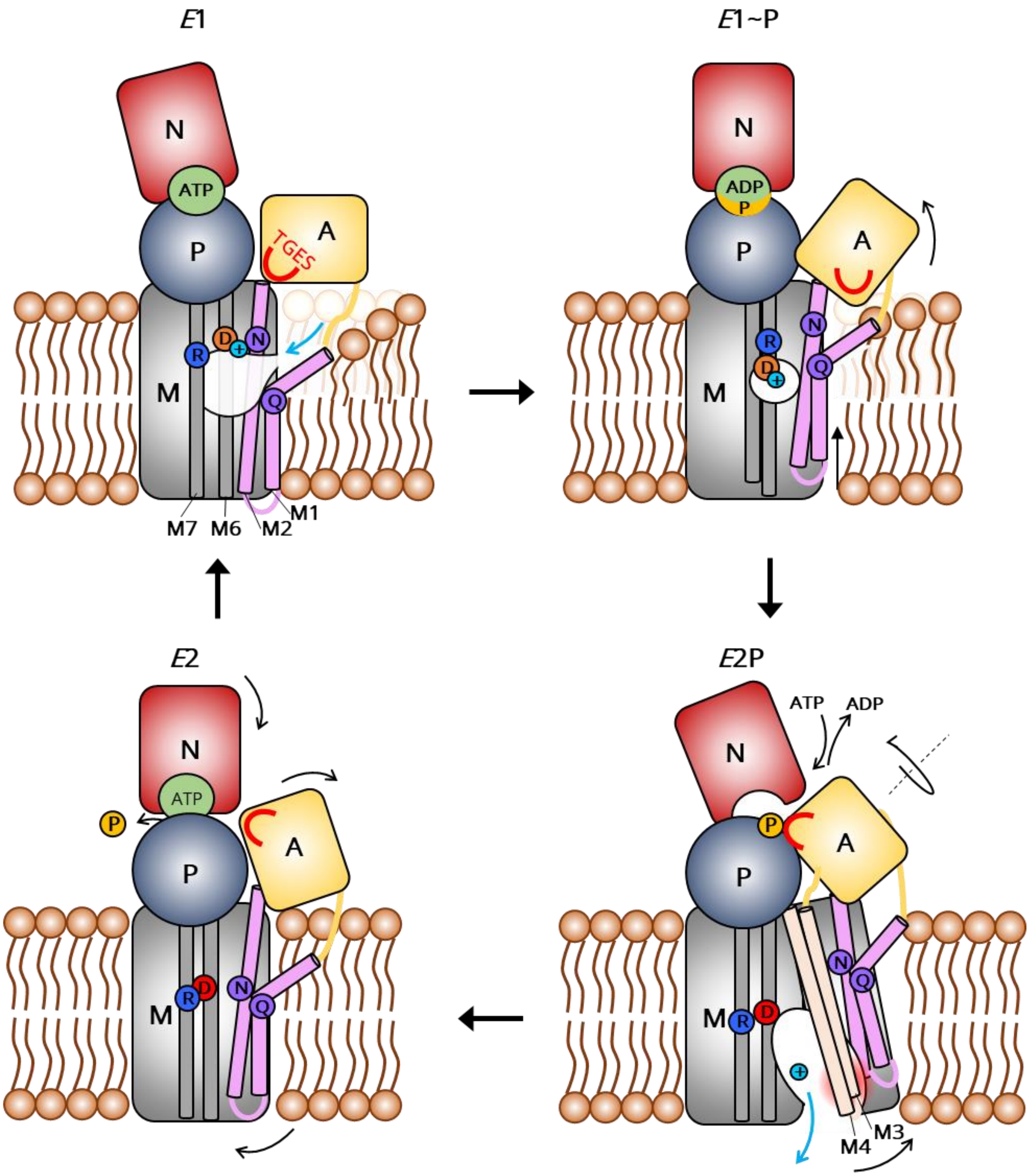

Fig. S11. Proposed model of the proton transport mechanism in Pma1, based on homology models with SERCA. In the *E*1 state, a proton enters from the cytosol and binds between D730 and N154, facilitated by a local membrane depression. The proton gets occluded by a concerted upward movement of M1-2. The side chain of R695 in M7 appears to shield the protonated D730 from the former ion entry region. A large conformational change is expected to follows phosphoryl transfer to the P domain, leading to the transient opening of the extracellular proton exit pathway in the *E*2P state. In this state, N154 in M2 could interact with Q125 at the M1 kink to stabilise the bundle, and R695 can form a salt bridge with D730, favouring its deprotonation. A cluster of negatively charged residues (red shaded area) facilitates proton exit. In the subsequent dephosphorylation reaction, the exit pathway closes, leading to the *E*2 state, from which the pump cycles back to open up to the cytosol once again. Residues shown as coloured circles are: D (orange): protonated D730; D (red): deprotonated D730, N (purple): Asn154; Q (purple): Q125; R (blue): Arg695.

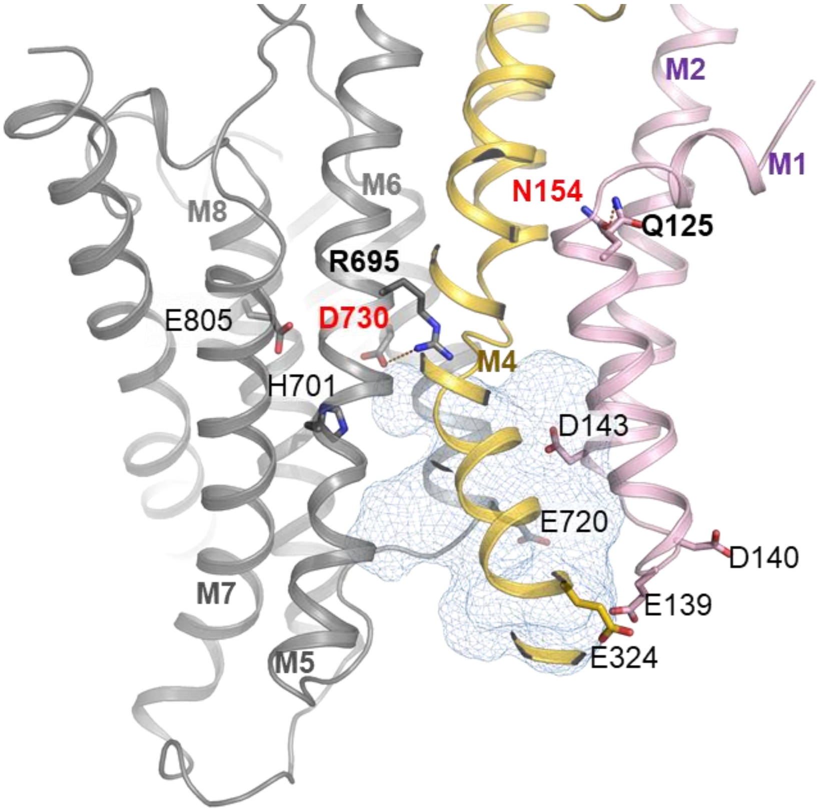

Fig. S12: Proton exit funnel in the open-to outside *E*2P homology model. Aqueous cavity representing the proton exit funnel between M1, M4 and M6. Important residues for proton transport are shown as sticks. M1-2 are coloured pink, M3-4 gold and M5-10 grey. The proton acceptor/donor Asp730 (labelled in red) at the inner end of the funnel lies in bonding distance to Arg695 (C-alpha distance: 7.3 Å; indicated bond: 4.9 Å), presuming a small side chain rotation of the latter. The *E*1-interaction partner of Asp730, Asn154 (labelled in red), has moved away and forms a putative hydrogen bond with Gln125 (2.8 Å). Putative bonds indicated as orange dashes. There is a clustering of negatively charged residues (Glu139, Asp140, Asp143, Glu324 and Glu720) at the extracellular end of the exit funnel.

A

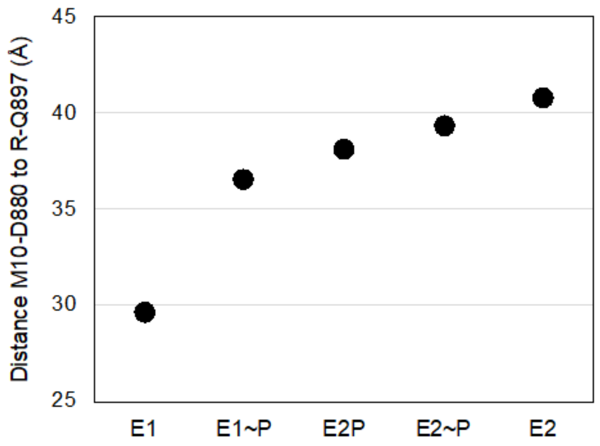

B

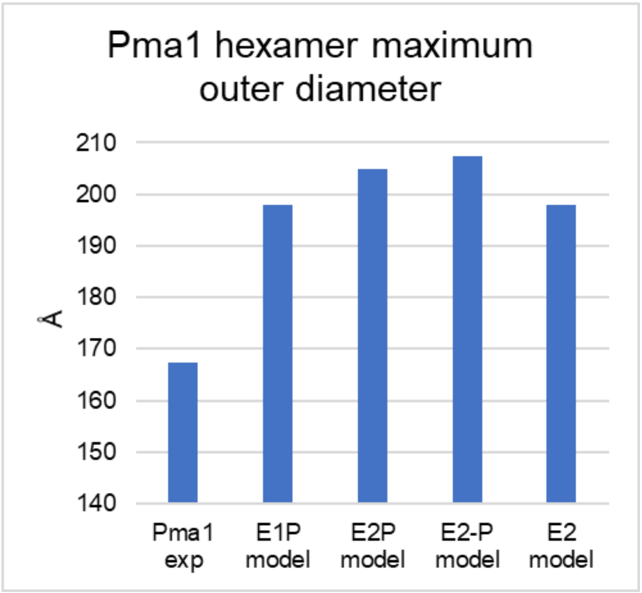

Fig. S13: Distance between M10 and the R helix, and hexamer homology model diameters
(**A**) Distance between the C-alpha atoms of the last residue of M10 (Asp880) and the first residue of the R-helix (Gln897) in the autoinhibited *E*1 structure and homology models throughout the catalytic *E*1/*E*2 cycle. The R-helix was placed into the homology models in its relative position to the P domain as observed in *E*1. The homology models were generated with SWISS-MODEL (64) based on a structural alignment and SERCA crystal structures with the PDB entries 1T5T (*E*1~P), 3B9B (*E*2P), 3N5K (*E*2~P), and 3NAL (*E*2). (**B**) Maximal outer diameter of the autoinhibited Pma1 *E*1 structure and homology models in states *E*1P, *E*2P, *E*2~P, and *E*2.

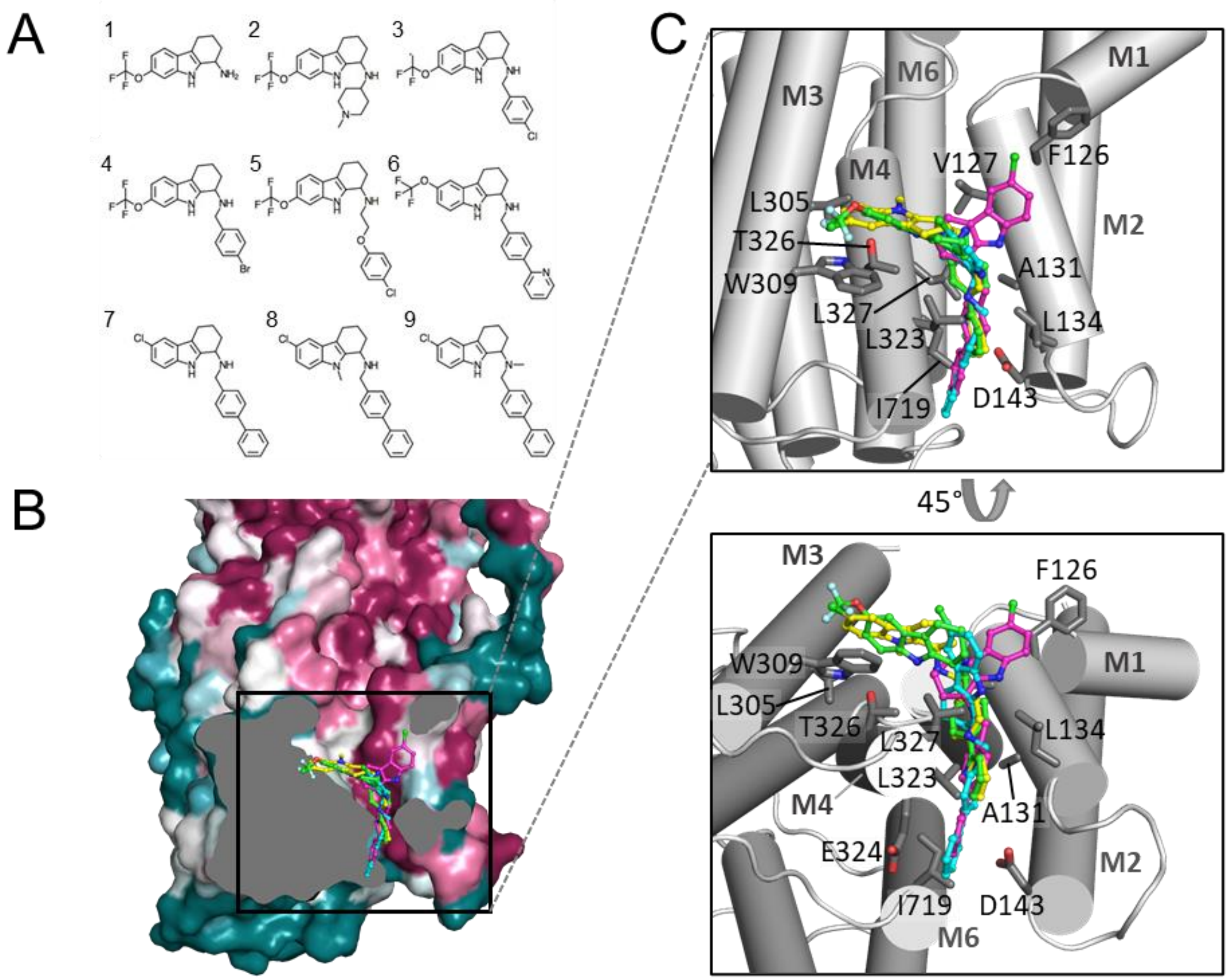

Fig. S14: Compound docking into Pma1. (**A**) Chemical structure of the docked tetrahydrocarbazole compounds. (**B**) Tetrahydrocarbazole compounds docked into Pma1 in the autoinhibited *E*1 state giving estimated affinities stronger than -9 kcal/mol (only one representative mode with the highest affinity score shown per compound): 6/S (green), 7/R (blue), 7/S (pink), 8/R (yellow). The protein surface is coloured according to conservation with proton pumps from human- and plant-pathogenic fungi from purple (conserved) to bluegreen (variable) (calculated with ConSurf (*42*)). (**C**) Enlarged view of the putative inhibitor binding site with the protein shown as grey cartoon. Residues involved in binding of most compounds are shown as sticks.

Table S1. Cryo-EM data collection, refinement and validation statistics.

**Data collection**		
Instrument	FEI Titan Krios / Gatan K3	
Magnification	105’000	
Voltage (kV)	300	
Electron dose (e^-/Å^2)	42	
Defocus range (µm)	-1.3 to -2.5	
Calibrated pixel size (Å)	0.837	
**Map values**	**hexamer**	**monomer**
Map ID	H3	M
EMDB ID	EMD-12644	EMD-12638
No. of particles	59’511	293’999
Map symmetry	C6	C1
Map resolution (Å)<br>FSC threshold 0.143	3.28	3.21
**Model**		
PDB ID	7NY1	7NXF
Protein residues	4’974	829
Ligands (no.)	K (6), Mg (6), ADP (6)	K (1), Mg (1), ADP (1)
**Model validation**		
Map CC (ligands)	0.76 (0.70)	0.80 (0.79)
MolProbity score	2.08	1.96
Clash score	12.01	8.99
Bond length rmsd (Å)	0.007	0.006
Bond angle rmsd (°)	1.142	0.816
B factor (Å^2) (min/max/mean)<br>Protein<br>ligands	 19.28/155.87/82.25<br>61.94/109.49/107.30	
Rotamer outliers (%)	0	
Cβ outliers (%)	0	
Ramachandran Plot<br>Favoured/allowed/outliers (%)	92.1 / 7.9 / 0	

Table S2: Residues involved in intra- and intermolecular contacts mediated via the R domain.

R – P		R – P’		R – R’/ R” – R	
Pro893	G589	Phe905	Val562	Leu902	Ser892
Lys 894	M592	Leu909	Gly563		Pro893
Arg900	G594	Val912	Arg566		
Glu903	Ser595	Ser913	Asn577		
Asp904	Tyr598	Thr914	Ile578		
Val907	Asp599	His916	Tyr579		
Arg911	Glu602	Glu917	Arg583		
	Arg625		Asp500		
			Phe600		

Table S3: **Residues involved in the intermolecular contact within the M domain.**

M3 / M4	M7 / L7-8	M10
Thr295	Ile772	Ile862
Ile299	Thr775	Phe863
Ile302	Thr776	Cys869
Leu306	/	Ile870
Trp309	Gly784	Tyr876
Val310	Gly785	Ile877
Phe313	Ile786	
Tyr314	Gln788	
/		
Pro318		
Ile319		

Table S4: Alignment of *Neurospora crassa* Pma1 with the plasma membrane proton pumps of human-pathogenic (above black line) and plant-pathogenic (below black line) fungi.

Sequences are sorted in decending order according to their identity with Pma1. Accession codes: *N. crassa* (sp|P07038), *Sporothrix schenckii* (tr|A0A0F2M765), *Histoplasma capsulatum* (sp|Q07421), *Coccidioides immites* (tr|A0A0E1RVX1), *Blastomyces dermatitidis* (tr|T5BWH0), *Acremonium chrysogenum* (tr|A0A4Y6GPG7), *Trichophyton rubrum* (tr|A0A022W272), *Candida glabrata* (tr|Q6FXU5), *Candida auris* (tr|A0A2H0ZKV9), *Candida albicans* (sp|P28877), *Pneumocystis jirovecii* (tr|A0A0W4ZIS2), *Aspergillus fumigatus* (tr|Q96TH7), *Talaromyces marneffei* (tr|A0A093Y2U3), *Syncephalastrum racemosum* (ORZ00574.1), *Rhizopus stolonifera* (RCI02493.1), *Lichtheimia corymbifera* (CDH55078.1), *Cryptococcus gattii* (tr|A0A0D0U934), *Cryptococcus neoformans* (tr|O74242), *Claviceps purpurea* (CCE32805.1), *Colletotrichum gloeosphorioides* (KAF3800644.1), *Magnaporthe oryzae* (ELQ65709.1), *Fusarium oxysporum* (RKL39108.1), *Botrytis cinerea* (EMR90163.1), *Fusarium graminearum* (PCD22889.1), *Aspergillus niger* (GAQ33769.1), *Blumeria graminis* (AAK94188.1), *Sclerotinia slerotiorum* (tr|A7F838), *Mycosphaerella graminicola* (XP_003852209.1), *Cochliobolus heterostrophus* (tr|M2TMB0), *Rhizoctonia solani* (tr|A0A0B7FM75), *Ustilago maydis* (), *Puccinia graminis* (tr|A0A5B0P5Y0)

Neurospora crassa	--------------------MADHSASGAPALSTN--IESGKFDEKAAEAA-------A-	30
Sporothrix schenckii	---------------------MAESTSAAPALHTD--IPGGNFDEKAGAGE-------AA	30
Histoplasma capsulatum	-----------MAH-SA--------------ASGA--ASAAHFEKKTP----------EV	22
Coccidioides immites	-----------MAEPTG-STRPQGEGVPPNHLGTT--VPSGGFEGHDEKAH-----APAS	41
Blastomyces dermatitidis	-----------MSD-PT-ASGPQGHGIPPNHLGTN--FPSGDFSEKAT----------EP	35
Acremonium chrysogenum	--------------------MADNKAAGAPALDTN--IESGGFDEKRAQQD-------AP	31
Trichophyton rubrum	-------------MADH-AAQGQGQPVPPNHLGTA--VPSGQFGGGHKDEVQQHQHQQQT	44
Candida glabrata	--MSDVES	06
Candida auris	--MSATEP	06
Candida albicans	--MSATEP	06
Pneumocystis jirovecii	---------------------MDEKTEYVVRKDTVYYNYVSSFEEL---------DKPLK	30
Aspergillus fumigatus	--	00
Talaromyces marneffei	-----------MAE---------------RRI--S---YAADVENGQTN-----------	18
Syncephalastrum racemosum	--	00
Rhizopus stolonifera	--	00
Lichtheimia corymbifera	------------------------------MSAKN------------------------D	06
Cryptococcus gattii	MSD---------HEKVGHTEEVPTKESS---LENK------------------------V	24
Cryptococcus neoformans	MSD---------HEKVGHTEEIPTKESS---LENK------------------------V	24
Claviceps purpurea	---------------------MADNKVGAPALDTN--IESGQFDEKSRQA----------	27
Colletotrichum gloeosphorioides	---------------------MAESNAAAPAINTP--IEGHRFDEKAPVEA-------PA	30
Magnaporthe oryzae	------------------MADSTQPTGAGPAINTP--IESGKFDEKEGLAH-------NP	33
Fusarium oxysporum	--------------------MAEEKAAGAPALDTN--IETGGFDEKRGQA----------	28
Botrytis cinerea	MSHLPGQHDIHLHGTTGAESSNHSSDTPPNHLRTN--IEPGQFDEKNGGVQ-------HT	51
Fusarium graminearum	--------------------MAEEKAVGAPALDTN--IETGGFDEKRGQA----------	28
Aspergillus niger	-----------------------MSFTYPK--DMD--IEP-EYEEK-------------I	19
Blumeria graminis	--MAQNG-----------	05
Sclerotinia slerotiorum	----------------------------------M---AHGDVENGASA-----------	12
Mycosphaerella graminicola	-----------MAA---------------PRV--SF--SDKDLENGEGG-----------	19
Cochliobolus heterostrophus	--	00
Rhizoctonia solani	MSE-----------------PIVEKEAGPPAAAPT------------------------T	19
Ustilago maydis	--	00
Puccinia graminis	MSD-----------------PDPDPQLQPEKQSTH------------------------D	19

Neurospora crassa	-----YQPKPKV-EDDEDEDID---AL---IEDLE-----SHDGH--------------D	59
Sporothrix schenckii	PAAAAPPVKPKVEEEEEDEDID---AL---IEDLE-----SHDGH--------------G	65
Histoplasma capsulatum	AHE--EKKPPLPEEEDEDEDMD---AL---IEELE-----SQDGH--------------I	55
Coccidioides immites	EAH--FEKKPIPDDDEEEEDMD---AL---IEELE-----SQDGH--------------I	74
Blastomyces dermatitidis	VPN--EKKPPVPEDDDEDEDMD---AL---IEELE-----SQDGH--------------I	68
Acremonium chrysogenum	PADTAPPKAAPVEEEEEDEDMD---AL---IEDLE-----SHDGH--------------D	66
Trichophyton rubrum	SAAFDEKKGAMPEEDEEDEDMD---QL---IADLE-----SQDGH--------------I	79
Candida glabrata	NNEKPPQDVYEDEEMSEDDDID---AL---IEELQ-----SHHGM--------------G	41
Candida auris	TNEKIDKAV---ESDDEDEDID---QL---IIDLQ-----SNHNL--------------D	38
Candida albicans	TNEKVDKIV----SDDEDEDID---QL---VADLQ-----SNPGA--------------G	37
Pneumocystis jirovecii	DLEKGTEGVCSLEEDEEDEDIN---AL---IDELD-----SQDGD--------------Q	65
Aspergillus fumigatus	---KPWYA	05
Talaromyces marneffei	-TRT------STDINNDGPALDEYTALNRYISTARDRRRGSTSSAGGLNEGEKPKKRWWN	71
Syncephalastrum racemosum	--MPGRDPGKPNSAQWVD	16
Rhizopus stolonifera	-----------------------------------MSD-QEQSAKITGDLHQITVEDLYD	24
Lichtheimia corymbifera	PGDKVLEGT------------I------EMKAEVTNEK-PLRNTRDLPDIKNLTVEELYD	47
Cryptococcus gattii	QGEEVPATG-----TDEPKKKR------EYKEMEHKTE-GDLHAKVDMNTIQFTAADLYD	72
Cryptococcus neoformans	QGEEVPAAA---AADEEPRKKR------EYKEMEHKTE-GDLHAKVDMNTIQFTAADLYD	74
Claviceps purpurea	PAENAPKKVPAVDDDDEDEDID---AL---IEDLE-----SQDGH--------------D	62
Colletotrichum gloeosphorioides	AS-----KAKVEEDEEEDEDID---AL---IEDLE-----SQDGH--------------I	60
Magnaporthe oryzae	AAK-----PKVADEEEEDEDID---AL---IEDLE-----SVDGH--------------G	63
Fusarium oxysporum	PPPTHAPKAPVAEDEEPDEDMD---AL---IEDLE-----SEDGH--------------A	63
Botrytis cinerea	SADA----IKHEEEEEEDEDMD---AL---IDELE-----SQDGH--------------A	82
Fusarium graminearum	P-ATHNPKAPVAEDEEPDEDMD---AL---IEDLE-----SEDGH--------------E	62
Aspergillus niger	AREAKAPTDIHNDEGEEEEDID---TL---IVELE-----SVDGG--------------A	54
Blumeria graminis	-----------ATVFEEDEKLGEYGNLVRYISNFKDGRRMSTSGAS-I-LDLPQKKKWYQ	52
Sclerotinia slerotiorum	-----------ARPEYEDEKLGEYGQLVRYISKYKGREEGEKAAAE-EEENAPKKKGLFS	60
Mycosphaerella graminicola	-PERS---RKWSQAPGNIEDLDEYTALQKYISTYRDPKLAQQDEVANA-HAESQKKKPWQ	74
Cochliobolus heterostrophus	--------------------MDEYTALQKYILFYRDAKVNPHA------TPTVQVKKWWQ	34
Rhizoctonia solani	PGAGATPT--------EEKKKR------EYKDFGHDEE-KATHAKVDMAQIELKAEDLYD	64
Ustilago maydis	----MSDVE---HQQEKKVTKH------RDIDEEHANS-GPKHALVDMSTIELKAEDLYD	46
Puccinia graminis	P-TSITPTE---KASDASKKPG------RFDKTFESEE-KPVHALVDMSLIQLKAEDLYD	68

Table S4: continued

Neurospora crassa	AEEE--EEEATPGGGRVVPEDM--LQTDTRVGLTSEEVVQRRRKYGLNQMKEEKENHFLK	115
Sporothrix schenckii	ADEDDEEEESNVHGGRVIPEDM--LQTDTRIGLTDHEVVARRRKYGLNQMKEEKENLILK	123
Histoplasma capsulatum	DIED--DEDGEPGGARPVPDEL--LTTDTRHGLTDAEVVARRKKYGLNQMKEEKENLVLK	111
Coccidioides immites	YEED--EDTGEPGGARPVPEEL--LQTDTRMGLTDQEVTTRRKKFGLNQMKEEKENMILK	130
Blastomyces dermatitidis	DVED--EEDGEPGGARPVPEEL--LNTDTRQGLTDAEVLVRRKKYGLNQMKEEKENLVLK	124
Acremonium chrysogenum	AFDEE--EETQIGGGRVVPEDM--LQTDPRVGLTDAEVVNRRRKYGLNQMKEEKENLVLK	122
Trichophyton rubrum	DEIDE-EDEDQPGGERPVPDEL--LQTDTRTGLTDAEVTTRRKKYGLNQMKEEKENLILK	136
Candida glabrata	DDDDSEDEGHHTGSARVVPEEY--LQTDPSYGLTSDEVAHRRKKYGLNQMADERESMIVK	099
Candida auris	DDEESDDE-ANAGSFKAVPDEL--LQTQPEVGLTDDEVTKRRKKYGLNQMAEEQENLVLK	095
Candida albicans	DEE--EEE-ENDSSFKAVPEEL--LQTDPRVGLTDDEVTKRRKRYGLNQMAEEQENLVLK	092
Pneumocystis jirovecii	EDNM---EDTCPQSHRAVPEEL--LATDTRIGLTSQEVINRRKKYGHNKMKEEKENMVVK	120
Aspergillus fumigatus	FWRKD-AET---GGAFVCPDEW--LETDLRTGLASSEIETRRKKGGWNELTTEKTNFFVQ	059
Talaromyces marneffei	FGGSSTTTS---NEPFVAPDDW--VDTDIRAGLKGSDIEIRRRKTGYNELVTEKTNLFVQ	126
Syncephalastrum racemosum	-------------ELPPELEPY--LQTPPVTGLTDEQASERLERFGRNELKQKKRNKILH	061
Rhizopus stolonifera	KDKYD--------LSTMEQQDVMQLLQTQPEGLTSQEVNNRIEKFGRNKLETKEVNPILQ	076
Lichtheimia corymbifera	KDKYD--------LSTMEPGDVFVLLQTSSEGLTSNEAAARVEKFGYNKIEHKEQNPFLQ	099
Cryptococcus gattii	KDKVD--------IEHVVMEEVYQLLQCTDAGLTEAEATDRIGIFGPNKLEEKSENVILQ	124
Cryptococcus neoformans	KDKVD--------IEHVVMEEVYQLLQCTDAGLTEAEATDRIGIFGPNKLEEKSENVLLQ	126
Claviceps purpurea	AFEEE-EEEGAPGGGRVVPEEM--LQTDSRVGLTESEVTARRRKYGLNQMKEEKENLVLK	119
Colletotrichum gloeosphorioides	DEE---EEDTTPGTGRVVPEDQ--LQTDTRMGLTEAEVLNRRKKWGRNEMAEAKENLVLK	115
Magnaporthe oryzae	DLEEE-EEEAGPGAARVIPEDL--LQTSTRTGLTEQEVQARRRKYGLNQMKEEKENLIMK	120
Fusarium oxysporum	FD--E-EEETQPGGGRVVPEDQ--LQTDSRVGLTEAEVINRRRKWGLNQMKEERENMILK	118
Botrytis cinerea	EDEEAE-EENGPAQERPVPEDL--LQTDTRIGLTDSEVQARRKKWGLNQMKEEKENLFLK	139
Fusarium graminearum	ID--D-DEEATPGGGRVVPEDQ--LQTDSRVGLTEAEVIARRKKWGLNAMKEEQENMILK	117
Aspergillus niger	ELNT--QESHQTGRLRPIADNL--LQTDPMTGLDETEAILRRKRFGSNEMKEEKENLALK	110
Blumeria graminis	FGKTEQV----ADGFYETPVEW--LQTDWKNGLTTTEVEARRKKVGFNELTTEKENMFLT	106
Sclerotinia slerotiorum	KKKI-GS----DGSGFETPDDW--LNTGMRQGLSAHEVEARRKKTGWNELTTENESLFVK	113
Mycosphaerella graminicola	FWKKA-PKAEDDGDAMVVPEDW--LNADIRQGITNADVESRRKKFGWNEISTDKENLFIK	131
Cochliobolus heterostrophus	FWKSGSTTATPVADAGLVPDEL--LNTELRTGLSSHEVEERRKRYGFNEITSEKTNLLKQ	092
Rhizoctonia solani	KDKVD--------LETVVLDDVFTLLQCSEEGLTEVESKRRLELFGPNKLESKEQNPFLQ	116
Ustilago maydis	KAKVD--------IEAIELEDVWTLLQCNEGGLSEEECSRRRAIFGPNKIETEEPNPILQ	098
Puccinia graminis	KDKVD--------LEQVELDDVWALLQCTEEGLTEAEAQRRLEIFGPNKLETKEINPFLQ	120

Neurospora crassa	FLGFFVGPIQFVMEGAAVLAAGL-------EDWVDFGVICGLLLLNAVVGFVQEFQAGSI	168
Sporothrix schenckii	FLGYFVGPIQFVMEAAAVLAAGL-------EDWVDFGVIIALLLLNAAVGFVQEFQAGSI	176
Histoplasma capsulatum	FLSYFVGPIQFVMEAAAILAAGL-------EDWVDFGVICALLLLNACVGFVQEFQAGSI	164
Coccidioides immites	FLSYFVGPIQFVMEAAAVLAAGL-------EDWVDFGVICGLLLLNACVGFIQEFQAGSI	183
Blastomyces dermatitidis	FLSYFVGPIQFVMEAAAILAAGL-------EDWVDFGVICGLLLLNACVGFIQEFQAGSI	177
Acremonium chrysogenum	FLGFFVGPIQFVMEAAAVLAAGL-------EDWVDFGVICGLLLLNACVGFFQEYQAGSI	175
Trichophyton rubrum	FFSYFVGPIQFVMEAAAILAAGL-------RDWVDFGVICALLLLNACVGFIQEFQAGSI	189
Candida glabrata	FVMFFVGPIQFVMEAAAILAAGL-------SDWVDFGVICGLLMLNACVGFIQEFQAGSI	152
Candida auris	FIMFFVGPIQFVMEAAAILAAGL-------EDWVDFGVICGLLLLNASVGFIQEYQAGSI	148
Candida albicans	FVMFFVGPIQFVMEAAAVLAAGL-------EDWVDFGVICALLLLNAFVGFIQEYQAGSI	145
Pneumocystis jirovecii	FLMYFVGPVQFVMEAAAILAAGL-------QDWVDFGVICALLLLNAIVGFVQEFQAGSI	173
Aspergillus fumigatus	FIGYFRGPILYVMELAVLLAAGL-------RDWIDLGVICGILLLNAVVGWYQEKQAADV	112
Talaromyces marneffei	FIGYFRGPILYVMELAVLLAAGL-------RDWIDLGVIIGILMLNAIVGWYQEKQAADV	179
Syncephalastrum racemosum	FLSFFTGAIAYLMEISVILTAVT-------ADWIDFAIILGLLIVNALIGFIEESRADSA	114
Rhizopus stolonifera	FLGFMWNPLSWVMEAAAIVAIALSNGENRPPDYPDFIGIVLLLIANAVIGFMEERQAGNA	136
Lichtheimia corymbifera	FLGFMWNPLSWVMEAAAIVAIALSNGEGRPPDWPDFVGIVLLLLANSLIGFLEERQAGNA	159
Cryptococcus gattii	FLSFMWNPLSWVMEGAALVAIALSNGGGTPPDWQDFVGIVLLLFVNSTIGFVEERNAGNA	184
Cryptococcus neoformans	FLSFMWNPLSWVMEGAALVAIALSNGGGTPPDWQDFVGIILLLFVNSTIGFVEERNAGNA	186
Claviceps purpurea	FLSYFIGPIQFVMEAAAVLAAGL-------QDWVDFGVICGLLLLNAAVGFIQEFQAGSI	172
Colletotrichum gloeosphorioides	FFMFFVGPIQFVMEAAAVLAAGL-------EDWIDFGVICGLLLLNAVVGFVQEFQAGSI	168
Magnaporthe oryzae	FLGYFIGPVQFVMEAAVILAAGL-------QHWVDFAVICALLLLNACVGFIQEFQAGSI	173
Fusarium oxysporum	FLMFFVGPIQFVMEAAAVLAAGL-------EDWIDFGVICALLLLNACVGFIQEFQAGSI	171
Botrytis cinerea	FLGYFIGPIQFVMEAAAVLAAGL-------QDWVDFGVICALLLLNATVGFVQEYQAGSI	192
Fusarium graminearum	FLMFFVGPIQFVMEAAAVLAAGL-------EDWIDFGVICALLLLNACVGFIQEYQAGSI	170
Aspergillus niger	FVSFFVGPVQFVMEAAAVLAAYL-------RDWVDLGVICGLLLLNASVGFVQDFQAGSI	163
Blumeria graminis	FVSYFRGPILYVMELAVLLAAGL-------RDWIDFGVIIGILMLNAIVGWYQEKQAADV	159
Sclerotinia slerotiorum	FIGFFRGPVLYVMEIAVLLAAGL-------RDWIDFGVIIGILMLNAVVGWYQEKQAADV	166
Mycosphaerella graminicola	FLTFFTGPILYVMELAVLLAAGL-------RSWIDFGVIIAILLLNAAVGWYQEKQAADV	184
Cochliobolus heterostrophus	FIGYFTGPILYVMELAALLAAGL-------QDWVDFGVICGILLLNAIVGWYQEKQAADV	145
Rhizoctonia solani	FLGFMWNPLSWVMEGAALVAIALSNGGGRAPDWPDFVGIVLLLLINSAIGFYEERGAGNA	176
Ustilago maydis	FLSFMWNPLSWVMEGAAIVAIALSNGEGQGPDWPDFVGIVLLLLINSTIGFIEERNAGNA	158
Puccinia graminis	FLGFMWNPLSWVMEAAAIVAIALSNGEGEPPDWQDFVGIVLLLLINSAIGFYEERSAGNA	180

Table S4: continued

Neurospora crassa	VDELKKTLALKAVVLRDGTLKEIEAPEVVPGDILQVEEGTIIPADGRIVTD---------	219
Sporothrix schenckii	VDELKKTLALKAVVLRDGTLKEIEAPEVVPGDILQVEEGTIIPADGRIVTE---------	227
Histoplasma capsulatum	VDELKKTLALKAVVLRNGRLTEVEAPEVVPGDILQVEEGTIIPADGRIVTE---------	215
Coccidioides immites	VDELKKTLALKAVVLRNGRLSEIEAPEVVPGDILQVEEGTIIPADGRIVTE---------	234
Blastomyces dermatitidis	VDELKKTLALKAVVLRNGRLAEIEAPEVVPGDILQVEEGTIIPADGRIVTE---------	228
Acremonium chrysogenum	VDELKKTLALKAVVLRDGQLREVEAPDVVPGDILQVEEGTIIPADGRIVTE---------	226
Trichophyton rubrum	VDELKKTLALKAVVLRNGRLVEVEAPEVVPGDILQVEEGTIIPADGRVVTE---------	240
Candida glabrata	VDELKKTLANVAVVIRDGQLVEVPANEVVPGDILQLEDGTIIPADGRLVTE---------	203
Candida auris	VDELKKTLANTANVVRNGQLVEVQANEIVPGDILQLEDGTVIPADGRIVSE---------	199
Candida albicans	VDELKKTLANSALVVRNGQLVEIPANEVVPGDILQLEDGTVIPTDGRIVSE---------	196
Pneumocystis jirovecii	VDELKKTLALKATVLRDGRLVDIEASEVVPGDILQLEEGSIVPADGRIVTE---------	224
Aspergillus fumigatus	VASLKGDIAMKAVVIRDGQEQEILARELVTGDIIVVEEGTVIPADIRLICDYDKPEMFET	172
Talaromyces marneffei	VASLKGDIAMRAWVIRDGSEQEILARELVVGDIVILEEGQVVPADVRLICDYEQPGDFDK	239
Syncephalastrum racemosum	VAALQSSLALRTRCKRAGQLREMPSGDIAVGDILVLRLGDIVPADARLLDMD--------	166
Rhizopus stolonifera	VKALMDSLAPECKVRRDGEWKTLEASELVPGDIISIKLGDVIPADGRLLKA---------	187
Lichtheimia corymbifera	VKALMEALAPECKVKRDGEWQTMEAANLVPGDIISIKLGDVVPADGRLITA---------	210
Cryptococcus gattii	VKALMDSLAPKARVKRDGQWKEIESAELVPGDLIAFKHGDVCPSDCRLVEA---------	235
Cryptococcus neoformans	VKALMDSLAPKARVKRDGQWKEIESSELVPGDLIAFKHGDVCPSDCRLVEA---------	237
Claviceps purpurea	VEELKKTLALKAVVLRDGTLKEIEAPEVVPGDILQVEEGTIIPADGRIVTE---------	223
Colletotrichum gloeosphorioides	VAELKKTLALKAVVLRDGTLKEVEAPEVVPGDILQVEEGTIIPADGKIVTE---------	219
Magnaporthe oryzae	VDELKKTLALKAVVLRDGQLKEVEAPEVVPGDILQVEEGTIIPADGRIVTD---------	224
Fusarium oxysporum	VEELKKTLALKAVVLRDGTLKEVEAPEVVPGDILQVEEGTIIPADGRFVTE---------	222
Botrytis cinerea	VDELKKTLALKAVVLRDGRLYEIEAPEVVPGDILQIEEGTIIPADGRIVTD---------	243
Fusarium graminearum	VEELKKTLALKAVVLRDGTLKEIEAPEVVPGDILQVEEGTIIPADGRFVTE---------	221
Aspergillus niger	VKELKKSLALRAVVLRDGKMADVDAAELVPGDIVKVDEGTIIPADGRVMTN---------	214
Blumeria graminis	VASLKGDIALRTTVIRDGQQYEIKARELVPGDIVIVEDGNVVPADCRIISAYDNPNGWAE	219
Sclerotinia slerotiorum	VASLKGDIALKATVVRDGAEVEILARELVPGDIIVIEDGHVVPADARIICAYDDPNGYET	226
Mycosphaerella graminicola	VASLKGDIAMKATVVRDGQEQDIKARELVPGDIVVIEEGQSVPADSRLICDYEHPEDFEK	244
Cochliobolus heterostrophus	VASLKGDIAMKATVVRDNQQQTILARELVPGDIVVIEEGQTVPGDARLICSYDHPEDFEL	205
Rhizoctonia solani	VKALMDSLAPKAKVRRDGKWSEIESSDLVPGDMVAFKIGDVVPADCRLTEA---------	227
Ustilago maydis	VKALMDSLAPKARCKRDGKWIEIESSDLVPGDVIAFKIGDIVPGDCRLFDA---------	209
Puccinia graminis	VAALMDSLAPKAKVRRDGSWKEIESSQLVPGDIVAFKIGDVVPADNRLYDA---------	231

Neurospora crassa	------------------------------------DAFLQVDQSALTGESLAVDKHKGD	243
Sporothrix schenckii	------------------------------------DAFLQVDQSAITGESLAVDKHKSD	251
Histoplasma capsulatum	------------------------------------EAFLQVDQSAITGESLAVDKHKGD	239
Coccidioides immites	------------------------------------GAFLQVDQSAITGESLAVDKHKGD	258
Blastomyces dermatitidis	------------------------------------EAFLQVDQSAITGESLAVDKHKGD	252
Acremonium chrysogenum	------------------------------------DAFLQVDQSALTGESLAVDKHKGD	250
Trichophyton rubrum	------------------------------------DAFLQVDQSAITGESLAVDKHKGD	264
Candida glabrata	------------------------------------NCFLQVDQSAITGESLAVDKGYGD	227
Candida auris	------------------------------------NALLQVDQSAITGESLAVDKRHGD	223
Candida albicans	------------------------------------DCLLQVDQSAITGESLAVDKRSGD	220
Pneumocystis jirovecii	------------------------------------EAYLQVDQSAITGESLAVEKRKGD	248
Aspergillus fumigatus	YKEYLATANDDTLK---EKDD---DDEDGGIEARVGVSLIAVDQSAITGESLAVDKYMAD	226
Talaromyces marneffei	YKELLATMAEDTLK---EKGE---DDDE--EEHHTGHSIVAVDQSAITGESLAVDKYMGD	291
Syncephalastrum racemosum	------------------------------VLGGKPQADFLVDQSSLTGESLLVKKRAGD	196
Rhizopus stolonifera	------------------------------------HGDVSIDQAALTGESLPVGKEEGD	211
Lichtheimia corymbifera	------------------------------------HGAVSIDQAALTGESLPVGKEVGD	234
Cryptococcus gattii	------------------------------------I-DVSMDQAALTGESLPVGKHEGD	258
Cryptococcus neoformans	------------------------------------I-DVSMDQAALTGESLPVGKHEGD	260
Claviceps purpurea	------------------------------------DAFLQVDQSAITGESLAVDKHKGD	247
Colletotrichum gloeosphorioides	------------------------------------DAFLQVDQSAITGESLAVDKHQND	243
Magnaporthe oryzae	------------------------------------DAYLQVDQSAITGESLAVDKHKGD	248
Fusarium oxysporum	------------------------------------GCFVQVDQSAITGESLAVDKHAGD	246
Botrytis cinerea	------------------------------------DAFLQVDQSAITGESLAVDKHKGD	267
Fusarium graminearum	------------------------------------GCFCQVDQSAITGESLAVDKHHGD	245
Aspergillus niger	-------------------------------------SPIQVDQSSVTGESLAVDKHKGD	237
Blumeria graminis	YQRELEAQAGESN---NEKD----DDDEIGEKHGSGYALLAIDQSAMTGESLAVDKYVAD	272
Sclerotinia slerotiorum	YQQELLNQRSHELSEKEEDD----EDDAHGGKHGSGYALLAIDQSAMTGESLAVDKYVAD	282
Mycosphaerella graminicola	YKELREQHALNPEE--DPAGSEDAEGEEGEGIQHQGHSIIAADQSSITGESLAVDKYMGD	302
Cochliobolus heterostrophus	YMKLKAEDKFHDADPEDEKDDVDEEKFDEENPITQGHPLVACDQSSITGESLAVDKYMGE	265
Rhizoctonia solani	------------------------------------I-NVSIDQAALTGESLPVNKKTGD	250
Ustilago maydis	------------------------------------I-NVSCDQASLTGESLPVNKKLGD	232
Puccinia graminis	------------------------------------I-NVSIDQAALTGESLPAGKKVGD	254

Table S4: continued

Neurospora crassa	QVFASSAVKRGEAFVVITATGDNTFVGRAAALVNAASGGSGHFTEVLNGIGTILLILVIF	303
Sporothrix schenckii	QCYASSAVKRGEAFVVVTATGDNTFVGRAAALVNAASSGTGHFTEVLNGIGTVLLVLVIF	311
Histoplasma capsulatum	TCYASSAVKRGEAFMVITATGDNTFVGRGPALVNAASAGTGHFTEVLNGIGTVLLILVIL	299
Coccidioides immites	TCYASSAVKRGEAFMVITATGDNTFVGRAAALVSAASAGTGHFTEVLNGIGTVLLVLVIV	318
Blastomyces dermatitidis	TCYASSAVKRGEAFMVITSTGDNTFVGRAAALVNAAAAGTGHFTEVLNGIGTVLLILVIL	312
Acremonium chrysogenum	NCFSSSAVKRGEAFIVVTATGDNTFVGRAAALVSQSAGGTGHFTEVLNGIGTILLVLVIV	310
Trichophyton rubrum	HCYASSSIKRGEAFMVVTSTGDNTFVGRAAALVNAASAGSGHFTEVLNGIGTVLLILVIF	324
Candida glabrata	QTFSSSTVKRGEAFMVVTATGDNTFVGRAAALVNKASGGQGHFTEVLNGIGILLLVLVIV	287
Candida auris	STYSSSTVKTGEAFMIVTATGDSTFVGRAAALVNKASSGSGHFTEVLNGIGTILLVLVIV	283
Candida albicans	SCYSSSTVKTGEAFMIVTATGDSTFVGRAAALVNKASAGTGHFTEVLNGIGTTLLVFVIV	280
Pneumocystis jirovecii	SIYSSSTVKRGETFMIVTATGDATFVGHAASLVNKASCGTGHFTDVLNRIGTILLVLVVF	308
Aspergillus fumigatus	TCYYTTGCKRGKAYAIVTATAKQSFVGKTAALVQGA-KDQGHFKAVMDNIGTTLLVLVMF	285
Talaromyces marneffei	TCYYTTGCKRGKAYAVVTATAKHSFVGKTASLVQGA-QDQGHFKAIMNSIGTSLLVLVMF	350
Syncephalastrum racemosum	LVYSSCIVKQGQQLAVVARTGPDTFIGRAASLIGAT-TDAGHFQKVINYIGNFLILLSIL	255
Rhizopus stolonifera	EVFSGSTVKQGEAEAVVIGTGTNTFFGRAAKLVGEAGDDEGHLQSILAKIGNFCLITISI	271
Lichtheimia corymbifera	EVFSGSTVKQGEAEAVVIGTGLNTFFGRAAKLVGEAGDELGHLQTILASIGNFCLCSIGI	294
Cryptococcus gattii	ECFSGSTCKQGEAEGIVIATGPNTFFGRAATLVGQDNDQVGHLQQVLARIGTFCLVSIGI	318
Cryptococcus neoformans	ECFSGSTCKQGEAEGIVIATGPNTFFGRAATLVGQDNDQVGHLQQVLARIGTFCLVSIGI	320
Claviceps purpurea	NCYASSAVKRGEAFVVVTATGDNTFVGRAAALVSQSAGGTGHFTEVLNGIGTVLLILVIV	307
Colletotrichum gloeosphorioides	SCYASSAIKRGEAFIIVTATGDNTFVGRAAALVNAANSGTGHFTEVLNGIGTILLVLVVF	303
Magnaporthe oryzae	QCYASSAVKRGEAFVVITATGDSTFVGRAAALVNAASAGTGHFTEVLNGIGTVLLILVVF	308
Fusarium oxysporum	NCYASSAVKRGEAFVIVTATGDNTFVGRAAALVSQSAGGTGHFTEVLNGIGTILLVLVVA	306
Botrytis cinerea	TCYASSGVKRGEAFMVITATGDHTFVGRAAALVNQASAGTGHFTEVLNGIGTVLLILVIF	327
Fusarium graminearum	NCYASSAVKRGEAFVIVTATGDNTFVGRAAALVSQSAGGTGHFTEVLNGIGTILLVLVVA	305
Aspergillus niger	VCYASSTVKRGYARILITATGNWTSVGRAAALVNAASTGTGHFTEVLHGISIVLLVLVVI	297
Blumeria graminis	VIYYTTGCKRGKAYAIVTHSAKMSFVGRTASLVSGA-QDQGHFKAIMNSIGTALLVLVVA	331
Sclerotinia slerotiorum	VIYYTTGCKRGKAYAVVTHGARMSFVGRTASLVTGA-QDQGHFKAIMNSIGTSLLVLVVG	341
Mycosphaerella graminicola	TVYYTTGCKRGKAYGVVLTSAKFSFIGRTATLVQGA-KDQGHFKAIMNSIGTALLVLVMF	361
Cochliobolus heterostrophus	VAYYTTGCKRGKAYGIVITTAKHSFVGRTATLVQGA-QDQGHFKAIMNSIGTALLVLVMF	324
Rhizoctonia solani	QCFSGSTCKQGEAEGVVIATGANTFFGRAASLVGQDDDTTGHLQKILAQIGSFCLVSIGL	310
Ustilago maydis	QCFSGSICKQGEAEGVVISTGPNTFFGRAATLIGSDDDTTGHLQQVLSRIGLFCMVTIGL	292
Puccinia graminis	QCFSGSTCKQGEAEGVVIATGANTFFGRAAALVGADDDSSGHLQKILAQIGTFCLVSIGI	314

Neurospora crassa	TLLI----VWVSSFYRSNP----------IVQILEFTLAITIIGVPVGLPAVVTTTMAVG	349
Sporothrix schenckii	TNLI----VWVSSFYRSNP----------IVTILEYTLAITIIGVPVGLPAVVTTTMAVG	357
Histoplasma capsulatum	TLLV----VWVSSFYRSNS----------IVTILEFTLAITIIGVPVGLPAVVTTTMAVG	345
Coccidioides immites	TLLV----VWVSSFYRSNG----------IVTILEFTLAITIIGVPVGLPAVVTTTMAVG	364
Blastomyces dermatitidis	TLLV----VWVSSFYRSND----------IVTILEFTLAITIIGVPVGLPAVVTTTMAVG	358
Acremonium chrysogenum	TLLV----VWIASFYRSNG----------IVHILEFTLGITIVGVPVGLPAVVTTTMAVG	356
Trichophyton rubrum	TLLV----AWVASFYRSNG----------IVTILEFTLAITIIGVPVGLPAVVTTTMAVG	370
Candida glabrata	TLLG----VWAACFYRTDN----------IVKILRFTLGITIIGVPVGLPAVVTTTMAVG	333
Candida auris	TLLV----VWVACFYRTVK----------IVPILRYTLAITIVGVPVGLPAVVTTTMAVG	329
Candida albicans	TLLV----VWVACFYRTVR----------IVPILRYTLAITIIGVPVGLPAVVTTTMAVG	326
Pneumocystis jirovecii	TLLV----VYISAFYRSTN----------TITILKFTLAITIIGVPVGLPAVVTTTMAVG	354
Aspergillus fumigatus	WILA----AWIGGFYRHLKIATPEHE---DNNLLHYTLILLIIGVPVGLPVVTTTTLAVG	338
Talaromyces marneffei	FILA----AWVGGFFRHLKLATPEWS---DNSLLRYTLILFIIGVPVGLPVVTTTTLAVG	403
Syncephalastrum racemosum	LVAV----LFAYDL-IELKIRTGTVTGDNVLETLKEMVVLTIAAIPVGLPTVMSVTMAVG	310
Rhizopus stolonifera	FLVVVIIVQYARFRYNY-------------RRGIDNILVLLIGGIPIAMPTVLSVTLAIG	318
Lichtheimia corymbifera	FVVLEIIIMYAAFHYSY-------------RRGIDNILVLLIGGIPIAMPTVLSVTLAIG	341
Cryptococcus gattii	FVLLEILIMYADFRFPY-------------RRGLNNILVLLIGGIPIAMPTVLSVTLAVG	365
Cryptococcus neoformans	FVLLEILILYADFRYPY-------------RRGLDNILVLLIGGIPIAMPTVLSVTLAVG	367
Claviceps purpurea	TLLV----VWISSFYRSNG----------IVEILTFTLAITIVGVPVGLPAVVTTTMAVG	353
Colletotrichum gloeosphorioides	TLLI----VWVSSFYRSNG----------IVDILRFTLAITIIGVPVGLPAVVTTTMAVG	349
Magnaporthe oryzae	TNLV----VWISSFYRNNS----------IVQILEFTLAITIIGVPVGLPAVVTTTMAVG	354
Fusarium oxysporum	TLLI----VWVSSFYRSNG----------IVDILRFTLAITIVGVPVGLPAVVTTTMAVG	352
Botrytis cinerea	TNLV----VWISSFYRSNP----------IVLILEYTLAITIIGVPVGLPAVVTTTMAVG	373
Fusarium graminearum	TLLI----VWVSSFYRSNG----------IVDILRFTLAITIVGVPVGLPAVVTTTMAVG	351
Aspergillus niger	TLIV----VWVSSFYRSNN----------TTTILEFTLAITMIGVPVGLPAVVTTTMAVG	343
Blumeria graminis	FILA----SWVGGFFHHLPIATPEGS---SINLLHYALILLIVGVPVGLPVVTTTTLAVG	384
Sclerotinia slerotiorum	WILI----AWIGGFFHHLQLATPEHS---SVNLLHYALILLIVGVPVGLPVVTTTTLAVG	394
Mycosphaerella graminicola	WILV----AWIGGFFRGINLATPEAS---SNTLLHYVLILFIIGVPVGLPVVTTTTLAVG	414
Cochliobolus heterostrophus	FILL----AWIGGFFRHIPIATAREGTDKSVTLLHYALIMFIVGVPVGLPVVTTTTLAVG	380
Rhizoctonia solani	FVLLEIVILYPRFHYSY-------------RRGLDNILVLLIGGIPIAMPTVLSVTLAVG	357
Ustilago maydis	FIVLEILILYAGFRYRY-------------RRGIDNILVLLIGGIPIAMPCVLSVTLAVG	339
Puccinia graminis	FIIIEILVMYAGFRYRY-------------RRGINNLLVLLIGGIPIAMPTVLSVTLAVG	361

Table S4: continued

Neurospora crassa	AAYLAKKKAIVQKLSAIESLAGVEILCSDKTGTLTKNKLSLHDPYTVA---GVDPEDLML	406
Sporothrix schenckii	AAYLARKKAIVQKLSAIESLAGVEILCSDKTGTLTKNKLSLSEPYTVA---GVEPEDLML	414
Histoplasma capsulatum	AAYLAKKKAIVQKLSAIESLAGVEILCSDKTGTLTKNKLSLAEPYCVS---GVDPEDLML	402
Coccidioides immites	AAYLAKKKAIVQKLSAIESLAGVEILCSDKTGTLTKNKLSLSEPYTVA---GVDPEDLML	421
Blastomyces dermatitidis	AAYLAKKKAIVQKLSAIESLAGVEILCSDKTGTLTKNKLSLADPYCVA---GVDPEDLML	415
Acremonium chrysogenum	AAYLAKKQAIVQKLSAIESLAGVEILCSDKTGTLTKNKLSLSEPFTVD---GVDPDDLML	413
Trichophyton rubrum	AAYLAKKKAIVQKLSAIESLAGVEILCSDKTGTLTKNKLSLAEPYCVS---GVDPEDLML	427
Candida glabrata	AAYLAKKQAIVQKLSAIESLAGVEILCSDKTGTLTKNKLSLHEPYTVE---GVSADDLML	390
Candida auris	AAYLAKKQAIVQKLSAIESLAGVEILCSDKTGTLTKNKLSLHEPYTVE---GVEADDLML	386
Candida albicans	AAYLAKKQAIVQKLSAIESLAGVEILCSDKTGTLTKNKLSLHEPYTVE---GVEPDDLML	383
Pneumocystis jirovecii	AAYLAKKKAIVQRLSAIESLAGVEILCSDKTGTLTKNNLSLAEPYTVD---GISCDELML	411
Aspergillus fumigatus	AAYLAEQKAIVQKLTAIESLAGVDILCSDKTGTLTANQLSIREPYVNE---GVDVNWMMA	395
Talaromyces marneffei	AAYLAQQKAIVQKLTAIESLAGVDVLCSDKTGTLTANQLSIREPYVAE---GVDVNWLFA	460
Syncephalastrum racemosum	AKQLAKRQVIIKRLTAVEELSSVSILCTDKTGTLTLNELTLDEPYLPS---HYTKDDILL	367
Rhizopus stolonifera	AKQLAEHMAIVTRITAIEEMAAVTILCSDKTGTLTLNKLIVDKPTIKTYSDQYDGDAVIQ	378
Lichtheimia corymbifera	AKQLAEHKAIVTRITAIEEMAAVTILCSDKTGTLTLNKLIVDKPTVKCYA-EFDADEIML	400
Cryptococcus gattii	AQQLAKHKAIVTRITAIEELAGVTILCSDKTGTLTTNKLTIDKENVKCYSKWD-VEGVCL	424
Cryptococcus neoformans	AQQLAKHKAIVTRITAIEELAGVTILCSDKTGTLTTNKLTIDKENVKCYSKWD-VEGVCL	426
Claviceps purpurea	AAYLAKKQAIVQKLSAIESLAGVEILCSDKTGTLTKNKLSLSEPYTVQ---GVDPDDLML	410
Colletotrichum gloeosphorioides	AAYLAKKKAIVQKLSAIESLAGVEILCSDKTGTLTKNKLSLAEPYTVA---GVDPEDLML	406
Magnaporthe oryzae	AAYLAKKKAIVQKLSAIESLAGVEILCSDKTGTLTKNKLSLSEPYTVA---GVEPEDLML	411
Fusarium oxysporum	AAYLAKKQAIVQKLSAIESLAGVEILCSDKTGTLTKNKLSLAEPFCVA---GVEPDDLML	409
Botrytis cinerea	AAYLAKKKAIVQKLSAIESLAGVEILCSDKTGTLTKNKLSLAEPFTVP---GVEPDDLML	430
Fusarium graminearum	AAYLAKKQAIVQKLSAIESLAGVEILCSDKTGTLTKNKLSLAEPFCVA---GVEPDDLML	408
Aspergillus niger	AAYLAKKRAIVQKLSAIESLAGVEILCSDKTGTLTKNKLSLTEPYTVA---GVNPEDLML	400
Blumeria graminis	AAYLAKEKAIVQKLTAIESLAGVDVLCSDKTGTLTANQLSIREPFVAD---GVDVNWMMA	441
Sclerotinia slerotiorum	AAYLAEEKAIVQKLTAIESLAGVDVLCSDKTGTLTANQLSIREPFVAE---GVDVNWMMA	451
Mycosphaerella graminicola	AAYLAKQQAIVQKLTAIESLAGVDVLCSDKTGTLTANQLSIREPYVAE---GEDVNWMMA	471
Cochliobolus heterostrophus	AAYLAEQKAIVQKLTAIESLAGVDVLCSDKTGTLTANQLSLREPYVAE---GQDVNWMMA	437
Rhizoctonia solani	AQQLAKHKAIVTRITAIEELAGVTILCSDKTGTLTTNKLTIDKELVKTYGPFA-PQDVIL	416
Ustilago maydis	AQQLAKHKAIVTRITAIEELAGVDILCSDKTGTLTTNKLTIDTELVKLYSDWANVEDVIR	399
Puccinia graminis	AQQLAKYKAIVTRITAIEELAGVTILCSDKTGTLTTNKLTIDKTTVKTYGEYD-VDAVCL	420

Neurospora crassa	TACLAASRKKKGIDAIDKAFLKS-LKYYPRAKSVLSK-YKVLQFHPFDPVSKKVVAVVE-	463
Sporothrix schenckii	TACLAASRKKKGIDAIDKAFLKS-LRYYPRAKSVLSK-YKVINFFPFDPVSKKVTAIVE-	471
Histoplasma capsulatum	TACLAASRKKKGIDAIDKAFLKS-LRYYPRAKSVLTQ-YKVLEFHPFDPVSKKVSAVVL-	459
Coccidioides immites	TACLAASRKKKGIDAIDKAFLKS-LKYYPRAKSVLTQ-YKVLEFHPFDPVSKKVSAIVE-	478
Blastomyces dermatitidis	TACLAASRKKKGIDAIDKAFLKS-LRFYPRAKSVLTQ-YKVLEFHPFDPVSKKVSAVVM-	472
Acremonium chrysogenum	TACLAASRKKKGIDAIDKAFLKA-LKYYPRAKGVLSK-YKVLEFHPFDPVSKKVQAVVQ-	470
Trichophyton rubrum	TACLAASRKKKGIDAIDKAFLKS-LRYYPRAKSVLTQ-YKVLQFHPFDPVSKKVSAVVE-	484
Candida glabrata	TACLAASRKKKGLDAIDKAFLKS-LINYPKAKDALTK-YKVIEFHPFDPVSKKVTAVVE-	447
Candida auris	TACLAASRKKKGLDAIDKAFLKS-LINYPRAKAALTK-YKVLEFQPFDPVSKKVTAIVE-	443
Candida albicans	TACLAASRKKKGLDAIDKAFLKS-LINYPRAKAALPK-YKVIEFQPFDPVSKKVTAIVE-	440
Pneumocystis jirovecii	TACLAASRKKKGLDAIDKAFLKA-LRNYPAARSALSK-YRVIKFYPFDPVSKKVTAIVE-	468
Aspergillus fumigatus	VAAIASNHNVKNLDPIDKVTILT-LRRYPKAREILSRNWVTEKYTPFDPVSKRITTVCT-	453
Talaromyces marneffei	VAAIASSHNVKNLDPIDKVTILT-LRRYPKAREILARNWVTEKYTPFDPVSKRITTVCT-	518
Syncephalastrum racemosum	YAHLACETT--AQDPIELAVRDAAEKYHPQITPQEVHGYTVQTFTPFNPTDKMARATVLE	425
Rhizopus stolonifera	LSAYAARTE--NQDAIDFCIVNS-LPEPSLA----RGGITELEFKPFNPVIKRTQITYRS	431
Lichtheimia corymbifera	ISSYASRTE--NQDAIDFCIVNS-LPDSKQA----REGIEELEFKPFNPVIKRTEITYKR	453
Cryptococcus gattii	LAAYASRTE--NQDAIDGCVVGT-LPDPSQA----RAGIKLLDFKPFNPVDKRTEITYRD	477
Cryptococcus neoformans	LAAYASRTE--NQDAIDGCVVGT-LPDPQQA----RAGIKLLDFKPFNPVDKRTEITYRD	479
Claviceps purpurea	TACLAASRKKKGIDAIDKAFLKA-LKFYPRAKSVLSK-YKVLDFHPFDPVSKKVQALVE-	467
Colletotrichum gloeosphorioides	TACLAASRKKKGMDAIDKAFLKS-LRYYPRAKSVLSK-YQVLEFFPFDPVSKKVTAIVQ-	463
Magnaporthe oryzae	TACLAASRKKKGMDAIDKAFLKS-LKYYPRAKAALSK-YKVLEFHPFDPVSKKVTAIVE-	468
Fusarium oxysporum	TACLAASRKKKGIDAIDKAFLKA-LKYYPRAKSVLSK-YKVLDFHPFDPVSKKVQAVVE-	466
Botrytis cinerea	TACLAASRKKKGIDAIDKAFLKS-LRYYPRAKSVLSK-YKVLEFHPFDPVSKKVQAVVE-	487
Fusarium graminearum	TACLAASRKKKGIDAIDKAFLKA-LKFYPRAKGVLSK-YKVLDFHPFDPVSKKVQAVVE-	465
Aspergillus niger	TACLAASRKKKGMDAIDRAFFKA-LNEYPDAKTMLPQ-FKVLDFSPFDPVSKKVTAVVQ-	457
Blumeria graminis	VAALASSHNVKSLDPIDKVTILT-LKRYPRAKEILSQGWRTEKFTPFDPVSKRITAIVI-	499
Sclerotinia slerotiorum	VAALASSHNVKSLDPIDKVTILT-LKRYPAARKILEQGWKTENFTPFDPVSKRITAIVT-	509
Mycosphaerella graminicola	CAALASSHNIKSLDPIDKVTILT-LKRYPKARDILKDDWKTEKFTPFDPVSKRITTICT-	529
Cochliobolus heterostrophus	VAALASSHNLKSLDPIDKVTILT-IRRYPKAREILNMGWRTEKFTPFDPVSKRITAVCH-	495
Rhizoctonia solani	LAAYASRTE--NQDAIDQCVVGT-LDDPARA----RAGIKLLDFKPFNPVDKRTEITYRE	469
Ustilago maydis	LAAYASRTE--NQDAIDATIVGT-LKDPAEA----RNGIKLLDFKPFNPVDKRTEITYLE	452
Puccinia graminis	LAAYASRTE--NQDAIDTCVVGT-VGA-GPA----RAGIKLLDFKPFNPVDKRTEITYLE	472

Table S4: continued

Neurospora crassa	SP-QGERITCVKGAPLFVLKTVEEDHPIPEEVDQAYKNKVAEFATRGFRSLGVARKR---	519
Sporothrix schenckii	SP-QGERITCVKGAPLFVLKTVEEDHPIPEEVDQAYKNKVAEFATRGFRSLGVARKR---	527
Histoplasma capsulatum	SP-QGERITCVKGAPLSVLKTVEEDHPIPDEVDSAYKNKVAEFATRGFRSLGVARKR---	515
Coccidioides immites	SP-QGERITCVKGAPLFVLRTVEEDHPIPEEIDAAYKNKVAEFATRGFRSLGVARKR---	534
Blastomyces dermatitidis	SP-QGERITCVKGAPLFVLKTVEEDHPIPEEVDNAYKNKVAEFATRGFRSLGVARKR---	528
Acremonium chrysogenum	SP-QGEIITCVKGAPLFVLKTVEEDHPIPEDIDKAYKNCVAEFATRGFRSLGVARKR---	526
Trichophyton rubrum	SP-QGERIICVKGAPLFVLKTVEEDHPIPEDIDAAYKNKVAEFATRGFRSLGVARKR---	540
Candida glabrata	SP-EGERIVCVKGAPLFVLKTVEEDHPIPEDVHENYENKVAELASRGFRALGVARKR---	503
Candida auris	SP-EGERIVCVKGAPLFVLKTVEDDHPIPEDVHENYQNTVAEFASRGFRSLGVARKR---	499
Candida albicans	SP-EGERIICVKGAPLFVLKTVEDDHPIPEDVHENYQNTVAEFASRGFRSLGVARKR---	496
Pneumocystis jirovecii	SP-SGEKIVCVKGAPLFVLRTVEDDHPVSEDIQNAYKDKVAEFASRGYRSLGIARRI---	524
Aspergillus fumigatus	-C-DGVRYVCAKGAPKAILNMSQ----CSEEEAAKFREKAAEFARRGFRSLGVAVQK---	504
Talaromyces marneffei	-C-DGVRYTCAKGAPKAILNLSE----CSEEEARLYRDKAAEFARRGFRSLGVAVQK---	569
Syncephalastrum racemosum	LG-TQKTFQAAKGAPQVIIGLCGGHA--------EAEHVVEDMASRGLRCLGVARTV---	473
Rhizopus stolonifera	EA-DGKTYRVTKGMSHTVLDLCTRDK--TDATIKALNDDVDEFARRGLRALAVAIDEIPS	488
Lichtheimia corymbifera	LS-DGKVMRATKGMSAFIMDLCTRNK--TDQQIKQLEEDVDEFARRGLRSLAVAVDEVPS	510
Cryptococcus gattii	DMDGGKLKRATKGMTGIIIELCSRGK--TNELEDQLEADVEEFARRGLRALAVAYEDVLG	535
Cryptococcus neoformans	EMDGGKLKRATKGMTGIIIEICTRNK--TNELEDQLEADVEEFARRGLRALAVAFEDVAG	537
Claviceps purpurea	SP-QGERITCVKGAPLFVLKTVEEDHPIPEAVDKAYKNCVAEFATRGFRSLGVARKR---	523
Colletotrichum gloeosphorioides	SP-AGEQITCVKGAPLFVLKTVEQDHEIPEEIDQAYKNKVAEFATRGFRSLGVARKR---	519
Magnaporthe oryzae	SP-QGEIITCVKGAPLFVLKTVEEDHEIPEAVDQDYKNKVAEFATRGFRSLGVARKR---	524
Fusarium oxysporum	SP-QGERIICVKGAPLFVLKTVEEDHPIPEEVDAAYKNCVAEFATRGFRSLGVARKR---	522
Botrytis cinerea	SP-QGEKIICVKGAPLFVLKTVEEDHPIPEEIDQAYKNKVAEFATRGFRSLGVARKR---	543
Fusarium graminearum	SP-QGERIICVKGAPLFVLKTVEEDHPIPEEVDSAYKNCVAEFATRGFRSLGVARKR---	521
Aspergillus niger	SP-QGQRITCVKGSPLFVLKTVQQDHQIQEDIEQAYKNKVAEFATRGFRSLGVARKC---	513
Blumeria graminis	-K-DGVTYTCAKGAPKAILNLSN----CSKEDAEMYKSKVTEFARRGFRSLGVAVKK---	550
Sclerotinia slerotiorum	-K-DGVTYTCAKGAPSAILRMSE----CSAEVAGMYKAKAGEFARRGFRSLGVAVKE---	560
Mycosphaerella graminicola	-L-RGDRFTCAKGAPKAVLNLTE----CSKETADMFKDKATEFARRGFRSLGVAYQK---	580
Cochliobolus heterostrophus	-M-GGDKYVCAKGAPKAIVNLAN----CDEVTATLYKEKAAEFARRGFRSLGVAYQK---	546
Rhizoctonia solani	ES-SGRLKRVTKGMTGIIIELCTRNK--TDEVENQLEADVTEFAGRGLRALAVAYEELDH	526
Ustilago maydis	EA-TGKMKRVTKGMTSIIIDLCKRNK--TEAQENALEADVEEFANRGLRGLAVAFEEVPS	509
Puccinia graminis	EA-TGKMKRVTKGMTGVIIELCSRNK--TEDVENQLEADVEEFARRGLRALAVAFEDVPS	529

Neurospora crassa	----GE-GSWEILGIMPCMDPPRHDTYKTVCEAKTLGLSIKMLTGDAVGIARETSRQLGL	574
Sporothrix schenckii	----GE-GAWEILGIMPCSDPPRHDTARTINEAKSLGLSIKMLTGDAVGIARETSRQLGL	582
Histoplasma capsulatum	----GE-GSWEILGIMPCSDPPRHDTAKTINEAKTLGLSIKMLTGDAVGIARETSRQLGL	570
Coccidioides immites	----GE-GSWEILGIMPCSDPPRHDTAKTINEAKTLGLSIKMLTGDAVGIARETSRQLGL	589
Blastomyces dermatitidis	----GE-GSWEILGIMPCSDPPRHDTAKTINEAKTLGLSIKMLTGDAVGIARETSRQLGL	583
Acremonium chrysogenum	----GE-GAWEILGIMPCSDPPRHDTARTINEAKKLGLSIKMLTGDAVGIARETSRQLGL	581
Trichophyton rubrum	----GE-GSWEILGIMPCSDPPRHDTAKTVNEAKTLGLSIKMLTGDAVGIARETSRQLGL	595
Candida glabrata	----GE-GHWEILGVMPCMDPPRDDTAETVNEARRLGLRVKMLTGDAVGIAKETCRQLGL	558
Candida auris	----GE-GHWEILGIMPCMDPPRDDTAQTVNEARRLGLSVKMLTGDAVGIAKETCRQLGL	554
Candida albicans	----GE-GHWEILGIMPCMDPPRDDTAATVNEARRLGLRVKMLTGDAVGIAKETCRQLGL	551
Pneumocystis jirovecii	----GN-SNWEILGIMPCSDPPRCDTARTISEAIRLGLRIKMLTGDAVGIAKETARQLGM	579
Aspergillus fumigatus	----EG-EPWQLLGMYPMFDPPREDTAHTIAEAQHLGLSVKMLTGDALAIAKETCKMLAL	559
Talaromyces marneffei	----EG-EPWQLLGMYPMFDPPRDDTAHTITEAQNLGLSVKMLTGDAIAIAKETCKMLAL	624
Syncephalastrum racemosum	----ND-NEWQLVGLLTFLDPPRPDSAATLNECANNGIAVKMITGDQAAIASEVAGRLGM	528
Rhizopus stolonifera	GDVEGDGIGFKLVGLLPIYDPPRSDTKETIDRAIALGVSVKMITGDQLAIAKETGRRLGM	548
Lichtheimia corymbifera	GDPEGEGLGFRLVGLLPIYDPPRSDTKETIDRAIALGVQVKMITGDQLAIAKETGRRLGM	570
Cryptococcus gattii	DDPSAEGNGFELVGLLSIFDPPRSDTKKTIDDAMALGVKVKMVTGDQLAIAKETGRRLGL	595
Cryptococcus neoformans	DDPSAEGNGFELVGLLSIFDPPRSDTKKTIDDAMALGVKVKMVTGDQLAIAKETGRRLGL	597
Claviceps purpurea	----GE-GAWEILGIMPCSDPPRHDTARTISEAKQLGLSIKMLTGDAVGIARETSRQLGL	578
Colletotrichum gloeosphorioides	----GEHGAWEILGIMPCSDPPRHDTARTVNEAKVLGLSIKMLTGDAVGIARETSRQLGL	575
Magnaporthe oryzae	----GE-GAWEILGIMPCSDPPRHDTARTINEAKNLGLSIKMLTGDAVGIARETSRQLGL	579
Fusarium oxysporum	----GE-GAWEILGIMPCSDPPRHDTARTINEAKRLGLSIKMLTGDAVGIARETSRQLGL	577
Botrytis cinerea	----GE-GQWEILGIMPCSDPPRHDTARTINEAKTLGLSIKMLTGDAVGIARETSRQLGL	598
Fusarium graminearum	----GE-GAWEILGIMPCSDPPRHDTARTINEAKRLGLSIKMLTGDAVGIARETSRQLGL	576
Aspergillus niger	----GD-GEWEILGIMPCSDPPRHDTAKTIKEAQTLGLSIKMLTGDAVGIARETSRQLGL	568
Blumeria graminis	----GD-GDWQLLGMLPMFDPPREDTASTIAEAQVLGLSVKMLTGDAIAIAKETCKMLAL	605
Sclerotinia slerotiorum	----GN-GPWQLLGMLPMFDPPREDTAATIAEAQVLGLSVKMLTGDAIAIAKETCKMLAL	615
Mycosphaerella graminicola	----NN-DPWVLLGMLSMFDPPREDTAQTIIEAQQLGVPVKMLTGDAIAIAKETCKMLAL	635
Cochliobolus heterostrophus	----ND-GDWILLGLMSMFDPPREDTAQTIVEAQQLGVPVKMLTGDAIAIAKETCKMLAL	601
Rhizoctonia solani	DNHEGEGNGFELIGLLAIFDPPRDDTKQTIDDAIALGVKVKMVTGDQLAIAKETGRRLGL	586
Ustilago maydis	GEVEAEGNGFELLGLLAIFDPPRHDTKETLDNAQALGVRVKMVTGDQLAIAKETGRRLGL	569
Puccinia graminis	NDKDAPGNGFELIGLLAIFDPPRDDTQQTIDDAMLLGVRVKMVTGDQLAIAKETGRRLGL	589

Table S4: continued

Neurospora crassa	GTNIYNAERLGLGGGGDMPGS----EVYDFVEAADGFAEVFPQHKYNVVEILQQRGYLVA	630
Sporothrix schenckii	GTNVYNAERLGLGGGGDMPGS----EVYDFVEAADGFAEVFPQHKYNVVEILQQRGYLVA	638
Histoplasma capsulatum	GTNVYNAERLGLGGGGTMPGS----EVYDFVEAADGFAEVFPQHKYNVVEILQQRGYLVA	626
Coccidioides immites	GTNVYNAERLGLGGGGTMPGS----DIYDFVEAADGFAEVFPQHKYNVVEILQQRGYLVA	645
Blastomyces dermatitidis	GTNVYNAERLGLGGGGTMPGS----EVYDFVEAADGFAEVFPQHKYNVVEILQQRGYLVA	639
Acremonium chrysogenum	GTNVYNAERLGLGGGGDMPGS----EVYDFVEAADGFAEVFPQHKYSVVEILQQRGYLVA	637
Trichophyton rubrum	GTNIYNAERLGLGGGGTMPGS----DIYDFVEAADGFAEVFPQHKYNVVEILQQRGYLVA	651
Candida glabrata	GTNIYNAERLGLGGGGDMPGS----ELADFVENADGFAEVFPQHKYKVVEILQNRGYLVA	614
Candida auris	GTNIYDAEKLGLSGGGDMAGS----EIADFVENADGFAEVFPQHKYNAVEILQARGYLVA	610
Candida albicans	GTNIYDADRLGLSGGGDMAGS----EIADFVENADGFAEGFPTNKYNAVEILQSRGYLVA	607
Pneumocystis jirovecii	GTNVYNAERLGLGGGGDMPGS----EVYDFVEAADGFAEVFPQHKYNVVEILQQRGYLVA	635
Aspergillus fumigatus	STKVYDSERLIHGG---LAGS----AQHDLVEKADGFAEVFPEHKYQVVEMLQQRGHLTA	612
Talaromyces marneffei	GTKVYNSERLIHGG---LTGA----RQHDLVERADGFAEVFPEHKYQVVEMLQQRGHLTA	677
Syncephalastrum racemosum	GQMILDADYIANPA---RSED----EVVEDCLRADGFARVIPEHKYRVVELLQSRGYFVA	581
Rhizopus stolonifera	GDNMFLSKTLKEGP---PAGS-GYTDVDQMVLHADGFAGVYPEHKYEIVERLQAMGYMVA	604
Lichtheimia corymbifera	GDDMYLSQSLKEGP---PPGS-GYTTVDDLVLKCDGFAGVYPEHKYEIVERLQAMGHMTA	626
Cryptococcus gattii	GDHMYPAKVLKEGP---EPGS-KHANLDEMIMDADGFAGVFPEHKFEIVKRIQNLGHLCA	651
Cryptococcus neoformans	GDHMYPAKVLKEGP---EAGS-KHANLDEMIMDADGFAGVFPEHKFEIVKRIQNLGHLCA	653
Claviceps purpurea	GTNVYNAERLGLGGGGDMPGS----EVYDFVEAADGFAEVFPQHKYSVVEILQQRGYLVA	634
Colletotrichum gloeosphorioides	GTNIYNAERLGLGGGGDMPGS----EVYDFVEAADGFAEVFPQHKYNVVEILQQRGYLVA	631
Magnaporthe oryzae	GTNVYNAERLGLGGGGDMPGS----EVYDFVEAADGFAEVFPQHKYNVVEILQQRGYLVA	635
Fusarium oxysporum	GTNVYNAERLGLGGGGDMPGS----EVYDFVEAADGFAEVFPQHKYNVVEILQQRGYLVA	633
Botrytis cinerea	GTNVYNAERLGLGGGGDMPGS----EVYDFVEAADGFAEVFPQHKYNVVEILQQRGYLVA	654
Fusarium graminearum	GTNVYNAERLGLGGGGDMPGS----EVYDFVEAADGFAEVFPQHKYNVVEILQQRGYLVA	632
Aspergillus niger	GTNVYNAERLGLGGGGTMSGS----EVYDFVEAADGFAEVFPEHKYNVVDILQQRGYLVA	624
Blumeria graminis	GTKVYNSERLIHGG---LSGT----TQHDLVEKADGFAEVFPEHKYQVVEMLQQRGHLTA	658
Sclerotinia slerotiorum	GTKVYNSDKLIHGG---LTGT----TQHDLVERADGFAEVFPEHKYQVVEMLQQRGHLTA	668
Mycosphaerella graminicola	GTKVYNSQKLIHGG---LSGT----TQHDLVERADGFAEVFPEHKYQVVEMLQQRGHLTA	688
Cochliobolus heterostrophus	GTKVYNSSKLIHGG---LTGT----TQHDLVERADGFAEVFPEHKYQVVEMLQQRGHLTA	654
Rhizoctonia solani	GDHMYPAKVLQDGP---PPGG-KHLSLDEMIMDADGFAGVFPEHKYEIVKRLQGLGHLCA	642
Ustilago maydis	GDRMFNSKVLVEGV---LPAGSPYKSLDEMILDVDGFAGVFPEHKYEIVKRLQGLGHLTA	626
Puccinia graminis	GDHMYPAKVLKDGP---EPGG-KHLTLDEMILDADGFAGVFPEHKYEIVKRLQGLGHLCA	645

Neurospora crassa	MTGDGVNDAPSLKKADTGIAVEGSSDAARSAADIVFLAPGLGAIIDALKTSRQIFHRMYA	690
Sporothrix schenckii	MTGDGVNDAPSLKKADTGIAVEGASDAARSAADIVFLAPGLGAIIDALKTSRQIFHRMYA	698
Histoplasma capsulatum	MTGDGVNDAPSLKKADTGIAVEGASDAARSAADIVFLAPGLSAIIDALKTSRQIFHRMYA	686
Coccidioides immites	MTGDGVNDAPSLKKADTGIAVEGASDAARSAADIVFLAPGLSAIIDALKTSRQIFHRMYA	705
Blastomyces dermatitidis	MTGDGVNDAPSLKKADTGIAVEGASDAARSAADIVFLAPGLSAIIDALKTSRQIFHRMYA	699
Acremonium chrysogenum	MTGDGVNDAPSLKKADTGIAVEGASDAARSASDIVFLAPGLGAIIDALKTSRQIFHRMYA	697
Trichophyton rubrum	MTGDGVNDAPSLKKADTGIAVEGASDAARSAADIVFLAPGLSAIIDALKTSRQIFHRMYA	711
Candida glabrata	MTGDGVNDAPSLKKADTGIAVEGASDAARSAADIVFLAPGLSAIIDALKTSRQIFHRMYS	674
Candida auris	MTGDGVNDAPSLKKADTGIAVEGATDAARSAADIVFLAPGLSAIIDALKTSRQIFHRMYA	670
Candida albicans	MTGDGVNDAPSLKKADTGIAVEGATDAARSAADIVFLAPGLSAIIDALKTSRQIFHRMYS	667
Pneumocystis jirovecii	MTGDGVNDAPSLKKADTGIAVQGASDAARSAADIVFLAPGLSAIIDALKTSRQIFHRMYA	695
Aspergillus fumigatus	MTGDGVNDAPSLKKADCGIAVEGSTEAAQAAADIVFLAPGLSTIVDAIKLARQIFQRMKA	672
Talaromyces marneffei	MTGDGVNDAPSLKKSDCGIAVEGATEAAQAAADIVFLAPGLSTIVDSIKVARQIFQRMKA	737
Syncephalastrum racemosum	MTGDGVNDAAALKKANVGIAVHGSTDAARSASDIVLLTPGLAPIIDGIKTSRVIFQRLRS	641
Rhizopus stolonifera	MTGDGVNDAPALSKANVGVAVDDASDAARSAADIVLTSPGLSVIVEAIIGSRQIFQRMRN	664
Lichtheimia corymbifera	MTGDGVNDAPALSKSNVGVAVADASDAARSAADIVLTEPGLSVIIEAFVHSRRIFQRMRN	686
Cryptococcus gattii	MTGDGANDAPALSRANVGIAVEGATDAARGAADIVLTEPGLSTIVHAIYGSRVIFQRMRN	711
Cryptococcus neoformans	MTGDGANDAPALSRANVGIAVEGATDAARGAADIVLTEPGLSTIVHAIYGSRVIFQRMRN	713
Claviceps purpurea	MTGDGVNDAPSLKKADTGIAVEGASDAARSAADIVFLAPGLGAIIDALKTSRQIFHRMYA	694
Colletotrichum gloeosphorioides	MTGDGVNDAPSLKKADTGIAVEGASDAARSAADIVFLAPGLGAIIDALKTSRQIFHRMYA	691
Magnaporthe oryzae	MTGDGVNDAPSLKKADTGIAVEGASDAARSAADIVFLAPGLGAIIDALKTSRQIFHRMYA	695
Fusarium oxysporum	MTGDGVNDAPSLKKADTGIAVEGASDAARSASDIVFLAPGLGAIIDALKTSRQIFHRMYA	693
Botrytis cinerea	MTGDGVNDAPSLKKADTGIAVEGASDAARSAADIVFLAPGLGAIIDALKTSRQIFHRMYA	714
Fusarium graminearum	MTGDGVNDAPSLKKADTGIAVEGASDAARSASDIVFLAPGLGAIIDALKTSRQIFHRMYA	692
Aspergillus niger	MTGDGVNDAPSLKKADTGIAVEGSSDAARSAADIVFLAPGLSAIIDALKTSRQIFHRMYA	684
Blumeria graminis	MTGDGVNDAPSLKKSDCGIAVEGATEAAQAASDIVFLAPGLSTIVSAIKIARQIFQRMKA	718
Sclerotinia slerotiorum	MTGDGVNDAPSLKKSDCGIAVEGSTEAAQAAADIVFLAPGLNTIVSAIKIARQIFQRMKA	728
Mycosphaerella graminicola	MTGDGVNDAPSLKKADCGIAVEGSSEAAQAAADIVFLAPGLSTIVFAIKTARQIFQRMKA	748
Cochliobolus heterostrophus	MTGDGVNDAPSLKKSDCGIAVEGSTEAAQAAADIVFLAPGLSTIVLAIKTARQIFQRMKA	714
Rhizoctonia solani	MTGDGANDAPALSRANVGIAVEGATDAARGAADIVLTEPGLSTIVHAIRGSRQIFQRMRN	702
Ustilago maydis	MTGDGANDAPALARANVGVAVEGATDAARGAADIVLTEPGLSTIVEAIRQSRIIFGRMKN	686
Puccinia graminis	MTGDGANDAPALSRANVGIAVEGATDAARGAADIVLTEPGLSTIVHAIRQSRIIFQRMRN	705

Table S4: continued

Neurospora crassa	YVVYRIALSIHLEIFLGLWIAILNRSLNIELVVFIAIFADVATLAIAYDNAPYSQTPVKW	750
Sporothrix schenckii	YVVYRIALSIHLEIYLGLWIAILNRSLNIELVVFIAIFADVATLAIAYDNAPFSKTPVKW	758
Histoplasma capsulatum	YVVYRIALSLHLEIFLGLWIAILNTSLNLQLVVFIAIFADIATLAIAYDNAPFSKTPVKW	746
Coccidioides immites	YVVYRIALSLHLEIFLGLWIAILNTSLNLELVVFIAIFADIATLAIAYDNAPFSKTPVKW	765
Blastomyces dermatitidis	YVVYRIALSLHLEIFLGLWIAILNTSLNLELVVFIAIFADIATLAIAYDNAPFSKTPVKW	759
Acremonium chrysogenum	YVVYRIALSLHMEIFLGLWIAILNRSLNLNLIVFIAIFADIATLAIAYDNAPYSQTPVKW	757
Trichophyton rubrum	YVVYRIALSLHLEIFLGLWIAILNTSLNLQLVVFIAIFADIATLAIAYDNAPFSKTPVKW	771
Candida glabrata	YVVYRIALSLHLELFLGLWIIILNHSLDIELIVFIAIFADVATLAIAYDNAPFSQTPVKW	734
Candida auris	YVVYRIALSLHLEIFLGLWIAILDRSLNIDLVVFIAIFADVATLAIAYDNAPYDPKPVKW	730
Candida albicans	YVVYRIALSLHLELFLGLWIAILNRSLDINLIVFIAIFADVATLAIAYDNAPYDPKPVKW	727
Pneumocystis jirovecii	YVVYRIALSLHLEIFLGLWIVIFNHLMILELVVFIAIFADIATLAIAYDNAPYSLFPTKW	755
Aspergillus fumigatus	YIQYRIALCLHLEIYLVTSMIIIDETLNSELVVFIALFADLATIAVAYDNAHYEMRPVEW	732
Talaromyces marneffei	YIQYRIALCLHLEIYLVSTMIIIDETIPSDMIVFIALFADLATIAVAYDNAHYEQRPVEW	797
Syncephalastrum racemosum	YALYRITSTIHFLLFFVVTLAEDWQMPPIFLILISVLNDAATLVMAVDNVKPSPSPDMW	701
Rhizopus stolonifera	YSIYTCSVTIRIVVGFSILIWAFQFDFPPFMVLIIAMLNDGTIMTISKDRVRPSPYPDAW	724
Lichtheimia corymbifera	YSIYTCSVTIRIVVGFAIMCFAFEFDFPPFLVLIIAVINDGTIMTISKDRVRPSPFPDSW	746
Cryptococcus gattii	YAIYACAVTIRIVLCFAIMVFAWQFDFPPFMVLIIAVLNDGTIMTLSLDRVLPSTTPDSW	771
Cryptococcus neoformans	YAIYACAVTIRIVLCFAIMAFAWRFDFPPFMVLIIAVLNDGTIMTLSLDRVLPSTTPDSW	773
Claviceps purpurea	YVVYRIALSLHMEIFLGLWIAILNRVLNLQLVVFIAIFADIATLAIAYDNAPFSQTPVKW	754
Colletotrichum gloeosphorioides	YVVYRIALSIHLEIYLGLWIAILNRSLNIELVVFIAIFADIATLAIAYDNAPFSKTPVKW	751
Magnaporthe oryzae	YVVYRIALSIHLEIYLGILIAALNQSLNINLVAFIAIFADIATLAIAYDNAPYSKSPVKW	755
Fusarium oxysporum	YVVYRIALSLHMEIFLGLWIAILNRSLNIELVVFIAIFADIATLAIAYDNAPFSQTPVKW	753
Botrytis cinerea	YVVYRIALSIHLEIFLGLWIAILNRSLNIELVVFIAIFADVATLAIAYDNAPFSKTPVKW	774
Fusarium graminearum	YVVYRIALSLHMEIFLGLWIAILNRSLNIELVVFIAIFADIATLAIAYDNAPFSQTPVKW	752
Aspergillus niger	YVVYRIALSLHLEIFLGFWIATRNESLNLQLVVFIAIFADIATLAIAYDTAPFSKTPVKW	744
Blumeria graminis	YIQYRIALCLHLEIYLVTSMIIINETVRVDLIVFLALFADLATIAVAYDNAHFEIRPVEW	778
Sclerotinia slerotiorum	YIQYRIALCLHLEIYLVTSMVIINETIRVELIVFLALFADLATIAVAYDNAHFEQRPVEW	788
Mycosphaerella graminicola	YIQYRIALCLHLEIYLVTSIIIIREVIASELIVFIALFADLATVAIAYDNAHSEQRPVEW	808
Cochliobolus heterostrophus	YIQYRIALCLHLEVYLVTSMIILNETIRAELIVFLALFADLATVAVAYDNAHSEQRPVEW	774
Rhizoctonia solani	YAIYACAVTIRIVVCFAILAFAYQFDFPPFMVLIIALLNDGTIMTLSVDRVLPSNTPDAW	762
Ustilago maydis	YAAYAAAITIRVVVGFALLAFIWKSDFPPFMVLIIAFLNDGSIMTLSLDTVKPALEPQHW	746
Puccinia graminis	YSIYACAVTIRIVVGFAVLVFAYKFDFPPFMVLIIALLNDGTIMTLSVDRVLPSMTPDHW	765

Neurospora crassa	NLPKLWGMSVLLGVVLAVGTWITVTTMYAQG---------------ENGGIVQNFGNMDE	795
Sporothrix schenckii	NLPKLWGMSVLLGVVLAVGTWITVTTMYAHG---------------PNGGIVQNFGNLDE	803
Histoplasma capsulatum	NLPKLWGMSVLLGIVLAVGTWITLTTMLVGS---------------ENGGIVQNFGRTHP	791
Coccidioides immites	NLPKLWGMSVLLGIVLAVGTWITLTTMLVGT---------------EDGGIVQNFGVRDE	810
Blastomyces dermatitidis	NLPKLWGMSVLLGIVLAVGTWITLTTMLVGS---------------ENGGIVQNFGVRDE	804
Acremonium chrysogenum	NLPKLWGMSVLLGVVLAVGTWICLTTLYVGG---------------ENGGITQNRGDIDE	802
Trichophyton rubrum	NLPKLWGMSVLLGVILAIGTWITLTTLLVGG---------------KDGGIVQNFGQIDP	816
Candida glabrata	NLPRLWGMSIILGIVLAIGTWICLTTMFL-----------------PRGGIIQNFGSIDG	777
Candida auris	NLPRLWGMSIILGIILAVGTWITLTTMFM-----------------SKGGIVQNFGSIDG	773
Candida albicans	NLPRLWGMSIVLGIILAIGTWITLTTMLL-----------------PKGGIIQNFGGLDG	770
Pneumocystis jirovecii	NLPKLWGLSLLLGVALAIGSWIAVTTIYVND---------------NAYGIVQKYGNIDS	800
Aspergillus fumigatus	QLPKIWVISIVLGVLLAGATWIMRASLFL-----------------NDGGLIQNFGSPQE	775
Talaromyces marneffei	QLPKIWVISIILGILLAAGTWIVRGAMFM-----------------ANGGIIENFGHPQG	840
Syncephalastrum racemosum	RLRLLIVLSCVLAAVLSL---ISFGHFYI-----------------LRDVIKVSAGQLNT	741
Rhizopus stolonifera	NLREIFSYAIVYGLYLTASTVAFVAVCLKTTFFNRAFGLQLFT--------DPNDYVLHS	776
Lichtheimia corymbifera	NLFEIFSYAIVYGLYLAASTIVFFAVLYKTSFFQTHFGVQNFT--------DPNDPVLHA	798
Cryptococcus gattii	DLAEVFSFGVAYGVYLSASTIALYATMENTTFFEDRFGVEPLK---------GNSYGGHM	822
Cryptococcus neoformans	DLAEVFSFGVAYGVYLSASTIALYATMENTTFFEDRFGVEPLK---------GNSYGGHM	824
Claviceps purpurea	NLPKLWGMSVLLGVVLAVGTWIALTTMFAGG---------------ENGGIVQNFGNMDP	799
Colletotrichum gloeosphorioides	NLPKLWGMSVLLGVVLAVGTWITVTTMYAHG---------------PDGGIVQNFGNMDE	796
Magnaporthe oryzae	NLPKLWGMSVLLGVVLAIGTFITITTMFVHG---------------EDGGIVQNNGQIDA	800
Fusarium oxysporum	NLPKLWGMSVLLGVVLAVGTWIALTTMYANS---------------EDGGIVQNFGKIDE	798
Botrytis cinerea	NLPKLWGMSVLLGFVLAVGTWITLTTMIARG---------------EDGGIVQNFGVLDE	819
Fusarium graminearum	NLPKLWGMSVLLGVVLAVGTWIALTTMLANS---------------EDGGIVQNFGKIDE	797
Aspergillus niger	NLPKLWGMSILLGIVLAVGTWITLTTILTAG---------------ENGGIMQDYGKRDE	789
Blumeria graminis	QLPKIWIISVVLGILLAIGTWILRGSLFL-----------------PNGGMIDNFGSIQG	821
Sclerotinia slerotiorum	QLPKIWIISVVLGILLALGTWVMRGALFL-----------------PNGGFIENFGSIQG	831
Mycosphaerella graminicola	QLPKIWIISVILGIELAIATWIARGTFFM-----------------PGGGLIQNYGNFEE	851
Cochliobolus heterostrophus	QLPKIWFISVVLGLLLALATWVVRGTLFI-----------------PSGGIIQNFGAIQP	817
Rhizoctonia solani	DLAEIFAFAVAYGLYLTLSTIILVIVILETNFFEDKFGVSLESELDANGRKDHNDRQLHM	822
Ustilago maydis	DLTELFFCGSLYGLYQVASTLALYAVIYETTFFEDKFGVTPLHG-------NPNDPRLHM	799
Puccinia graminis	DLAEIFIFATFYGLYLALSTIILVVVIRETTFFPDRFGVDTIG--------DINDFKLHM	817

Table S4: continued

Neurospora crassa	VLFLQISLTENWLIFITRANGPFWSS-IPSWQLSGAIFLVDILATCFTIWGWFEHSD---	851
Sporothrix schenckii	VVFLEVSLTENWLIFITRANGPFWSS-IPSWQLTGAILVVDLLATFFTLFGFFQHGQ---	859
Histoplasma capsulatum	VLFLEISLTENWLIFITRANGPFWSS-IPSWQLSGAILLVDIIATLFTIFGWFVGGQ---	847
Coccidioides immites	VLFLEISLTENWLIFITRANGPFWSS-IPSWQLAGAILVVDIVATFFTLFGWFVGGQ---	866
Blastomyces dermatitidis	VLFLQISLTENWLIFITRANGPFWSS-IPSWQLAGAILVVDIVATFFTLFGWFVGGQ---	860
Acremonium chrysogenum	IVFLQVSLTENWLIFITRANGPFWSS-IPSWQLAGAVLTVDIIATLFAVFGWFRGYE---	858
Trichophyton rubrum	VLFLEISLTENWLIFITRANGPFWSS-IPSWQLSGAILVVDIIATLFTIFGWFVGGQ---	872
Candida glabrata	VLFLQISLTENWLIFVTRAVGPFWSS-IPSWQLAGAVFAVDIIATMFTLFGWFSQN----	832
Candida auris	ILFLQISLTENWLIFITRAQGPFWSS-IPSWQLAGAVLIVDIVATCFTLFGWWSQN----	828
Candida albicans	ILFLQISLTENWLIFVTRAQGPFWSS-IPSWQLSGAVLIVDIIATCFTLFGWWSQN----	825
Pneumocystis jirovecii	VMFLEISLTENWLIFITRANGPFWSS-LPSWQLFGAVFLVDVIATLFCIFGWFTGTKEHG	859
Aspergillus fumigatus	MIFLEVALTENWLIFVTRGGKT-----WPSWQLVGAIFVVDVLATLFCVFGWLSGDYRQT	830
Talaromyces marneffei	IIFLEVALTENWLIFVTRGGKT-----YPSWQLVGAIFGVDVLATLFCVFGWLTQPEHQV	895
Syncephalastrum racemosum	LMYLHISSAPHFVIFSTRVETFCWQS-IPSWPFTLVVLGTQVVALVLSVYGMFGYQSNIE	800
Rhizopus stolonifera	VVYLQVSTISQGLIFITRSRGPFFSE-RPSILLVCSFIIAQLVATFIAVYANWGFTSIQG	835
Lichtheimia corymbifera	VIYLQVASISQGLIFVTRSHGFFFME-RPSTLLMCAFVVAQIVATFISVYAQWGFTDIQG	857
Cryptococcus gattii	VIYLQVAIISQALIFVTRSHGPSWTE-RPSVALMMAFCLAQLVSSIIAAYADWSFSEVHS	881
Cryptococcus neoformans	VIYLQVAIISQALIFVTRSHGPSWTE-RPSVALMLAFCLAQLVSSIIAAYADWSFSQVHS	883
Claviceps purpurea	VLFLEISLTENWLIFITRANGPFWSS-IPSWQLSGAILVVDIIATFFCLFGWFVHNT---	855
Colletotrichum gloeosphorioides	VLFLEISLTENWLIFVTRANGPFWSS-IPSWQLSGAILVVDILATLFCIFGWFQHNE---	852
Magnaporthe oryzae	VVFLEISLTENWLIFITRANGPFWSS-IPSWQLTGAIFVVDIIASCFAIWGWFVGNK---	856
Fusarium oxysporum	VLFLEISLTENWLIFITRANGPFWSS-IPSWQLSGAILIVDILATLFCIFGWFVGG----	853
Botrytis cinerea	VVFLEISLTENWLIFITRANGPFWSS-LPSWQLTGAILIVDIIATFFTLFGWFVGG----	874
Fusarium graminearum	VLFLEISLTENWLIFITRANGPFWSS-IPSWQLSGAILIVDILATLFCIFGWFVGG----	852
Aspergillus niger	VLFLEISLTENWLIFITRSDGAFWASKRPSWKLIGAIAAVDLVATCFCVFGWFAGGP---	846
Blumeria graminis	MLFLQISLTENWLIFVTRGDET-----YPAFALVAAIFGVDVLATLFCIFGWLTGGAGEQ	876
Sclerotinia slerotiorum	MLFLEVSLTENWLIFVTRGGNT-----WPSWQLVIAIFLVDVIATLFCVFGWLCGGAGEQ	886
Mycosphaerella graminicola	ILFLEIALTENWLIFITRGAQT-----LPSWQLVGAILGVDILATLFCIFGWLNSSIYQR	906
Cochliobolus heterostrophus	ILFLEVALTENWLIFVTRGGKT-----FPSFQLVIAILGVDALATIFTLFGWMSGAPYET	872
Rhizoctonia solani	IVYLQVAMISQALIFVTRSHGFFFME-RPSTALLGAFAIAQLVSSIIAAYADWGFTDIHS	881
Ustilago maydis	IMYLQVAILAQALIFVTRSHGFSWME-RPSFALMGAFCLAQLISSIIAAYGDWGFTNVHS	858
Puccinia graminis	IIYLQVAQISQALIFITRSHGFFFME-RPSFALMGAFCLAQLISSIIAAYGNWGFTQVEG	876

Neurospora crassa	----------------TSIVAVVRIWIFSFGIFCIMGGVYYILQDSVGFDNLMHGKSP-K	894
Sporothrix schenckii	---------------QTSIVAVVRIWIFSFGVFCIMGGVYYLLQDSVGFDNLMHGKSP-K	903
Histoplasma capsulatum	----------------TSIVAVVRIWVFSFGCFCVLGGLYYLLQGSAGFDNMMHGKSP-K	890
Coccidioides immites	----------------TSIVAVVRIWIFSFGVFCVMGGVYYILQGSTGFDNMMHGKSP-K	909
Blastomyces dermatitidis	----------------TSIVAVVRIWIFSFGCFCVLGGLYYLLQGSTGFDNMMHGKSP-K	903
Acremonium chrysogenum	-------------NRVNIVGVVRVWIFSFGVFAVMGGLYYFMQGSSGFDNLMHGKSP-K	903
Trichophyton rubrum	----------------TSIVAVVRIWVFSFGVFCVLGGIYYLLQGSTGFDNMMHGKSP-K	915
Candida glabrata	---------------WTDIVTVVKIYIWSIGVFCVLGGFYYIMSESVVFDRLMNGKPL-K	876
Candida auris	---------------WTDIVTVVRTWIWSFGVFCVMGGAYYLMSQSEAFDDFCNGRRN-K	872
Candida albicans	---------------WTDIVTVVRTWIWSFGVFCVMGGAYYLMSTSEAFDNFCNGRKP-Q	869
Pneumocystis jirovecii	-------------LEPTSVITVVRVWLFSFGVFCIMAGIYYLLSDSVAFDNIMHGKSV-K	905
Aspergillus fumigatus	--SPP-SHAEFSVNGDVDIVTVVVIWGYSIGVTIIIAVVYYILTIIPALDNLGRKTRS-K	886
Talaromyces marneffei	T-HPA-DPAQFSSDGRTSIVTVVIIWAYSIGVTIVVATVYAILTDIPWLDNLGRQTRS-K	952
Syncephalastrum racemosum	------------SIGWPLGLVVVA---ISLATFLLIDVIKV-VT-IRLWDRYTQHRRLAP	843
Rhizopus stolonifera	------------CGWHW-----AGVAWVWNFIWFAPLDLVKFAMQYFFEPKTSHQHNPE--	877
Lichtheimia corymbifera	------------SGWTL-----AGIAWVWNFVWFPPLDWLKFAMQRVFRPKTTAAAIQQ--	899
Cryptococcus gattii	-----------VSGGW-----IGIVWVWNIVWYFPLDAIKFIMKKTVIAALQRRKARKAG	925
Cryptococcus neoformans	-----------VSGGW-----IGIVWIWNIVWYFPLDGIKFIMKKTVIAALQRRKARKAG	927
Claviceps purpurea	---------------QTSIVAVVRIWIFSFGIFAVMGGLYYLMQGSAGFDNLMHGKSP-K	899
Colletotrichum gloeosphorioides	---------------QTSIVAVVRIWIFSFGVFCVCAGVYYMLEDSKGFDNLMHGKSP-K	896
Magnaporthe oryzae	---------------MTHIVAVVRIWVFSFGVFCIMGGLYYILQDSQGFDNLMHGKSP-K	900
Fusarium oxysporum	---------------QTSIVAVVRIWIFSFGVFCVMGGLYYFMQGSTGFDNLMHGKSP-K	897
Botrytis cinerea	---------------QTSIVAVVRIWVFSFGVFCIMGGVYYLLQDSSGFDNLMHGKSP-K	918
Fusarium graminearum	---------------QTSIVAVVRIWIFSFGVFCVMGGLYYFMQGSTGFDNLMHGKSP-K	896
Aspergillus niger	----------------TSWPTILRIYVFSFGVFCIMGGLYYLLQGSKSFDNIMHGKSP-V	889
Blumeria graminis	SDPAT-LNALLSTDGRTSIVTVIVVWCYSIAVTIVIAIVYHIMNKAAWLDNLGRFKRS-R	934
Sclerotinia slerotiorum	SDPAT-KNVLLSENGHTSIVTVVIVWGFSIGVTIITAIVYYLMNQWSWLDNLGRARRS-H	944
Mycosphaerella graminicola	PLPSPMSTFQQTANGHTDVVTVVVVWMFSIGVMIVIAITYYLLNKIPGLADLGRQNRS-L	965
Cochliobolus heterostrophus	N-PPT-INSRFRDDGWVDIVTVVVIWAYSIGVTIIIAIVYYMLNRIEWLDTLGRKDRS-R	929
Rhizoctonia solani	------------ISGGW-----IGIVWVWNIVWFFPLDWIKFAMKATVIKWFRTRRERKAA	925
Ustilago maydis	------------IEGGW-----IGIVWVWNIVWYFPMDLVKFAKFLLRNIRSKKTP----	898
Puccinia graminis	------------ISGGW-----IGIVWIWNIIWFLPMDFIKFSVKYGIRAWNAKRGVKPQS	920

Table S4: continued

Species	Sequence	Position
Neurospora crassa	GNQ-KQRSLEDFVVSLQRVST----------------Q----------------------	915
Sporothrix schenckii	GNQ-KQRSLEDFVVSLQRVST----------------Q----------------------	924
Histoplasma capsulatum	KNQ-KQRSLEDFVVSLQRVSTQHEKSS---------------------------------	916
Coccidioides immites	KSQ-KQRSLEDFVVSLQRVSTQHEKSA---------------------------------	935
Blastomyces dermatitidis	KNQ-KQRSLEDFVVSLQRVSTQHEKST---------------------------------	929
Acremonium chrysogenum	RSQ-KQRSLEDFVVSLQRVST----------------Q----------------------	924
Trichophyton rubrum	KSQ-KQRSLEDFGKPLPPPNILYTSIQLLMSSSSCLPPTCLYPA---------------R	959
Candida glabrata	-EEKSTRSVEDFLAAMQRVSTQHEKEN---------------------------------	902
Candida auris	KEQRDRRSLEDFMVSMARVSTQHEKSS---------------------------------	899
Candida albicans	-QHTDKRSLEDFLVSMQRVSTQHEKST---------------------------------	895
Pneumocystis jirovecii	KNT-KQRSLEDFVVALQRMSTKHEKGE---------------------------------	931
Aspergillus fumigatus	----ADTKIENMIAHLSKLAIEHETDN-NGKSYYTLGARAEVEE---------------D	926
Talaromyces marneffei	----ADTAIENMIGHLSKLAIEHEQDRATGNSRYVIAPRAPEEE---------------E	993
Syncephalastrum racemosum	AAIKKQQKMTRATLFQQK------QESRSG---YDRAQRRESVS----------------	878
Rhizopus stolonifera	-----EAKASASRRASAVSGTSSARYYANRTRSLRSFERPQNFGKKLLGMNKK--MDAKE	930
Lichtheimia corymbifera	-----GAGAPRSRRSSIVSTSASARYYANRTRSLRALERPRNFGRRLLGLNKKLSMDPTE	954
Cryptococcus gattii	PVVP--------EAGLQRAPSRHESLYSNRTNFLTRTANRLR-----GG--AKISMSQNE	970
Cryptococcus neoformans	PAVA--------DAALHRAPSRHESLYSNRTNFLTRAANRLR-----GG--AKISMSQNE	972
Claviceps purpurea	KDQ-KQRSLEDFVVSLQRVST----------------Q----------------------	920
Colletotrichum gloeosphorioides	GSQ-KQRSLEDFANCFTSRFP----------------AACLYPA---------------R	924
Magnaporthe oryzae	GNQ-KQRSLEDFVVSLQRVST----------------Q----------------------	921
Fusarium oxysporum	QNQ-KQRSLEDFVVSLQRVST----------------Q----------------------	918
Botrytis cinerea	KTQ-KQRSLEDFVVSLQRVST----------------Q----------------------	939
Fusarium graminearum	QNQ-KQRSLEDFVVSLQRVST----------------Q----------------------	917
Aspergillus niger	RTA-KQRSIEDFMVMMQRVSSQHVTSSLKGMESGQVTRGV--------------------	928
Blumeria graminis	----ADTQMENIIMHLSKIAIQHERDE-NGNSRFALVPKALEAE---------------D	974
Sclerotinia slerotiorum	----ADTQLENIIAHLSKVAIEHSQV--DGVHRYHIVQKQAEVE---------------D	983
Mycosphaerella graminicola	----HDTQMENIIGHLSKLALKHERDE-NGDARWTLATKATDDE---------------D	005
Cochliobolus heterostrophus	----KNPAIENMIAALSKLSLEHGTDK-HGTTRYVLAPRAVEEE---------------D	969
Rhizoctonia solani	EVARATGGEVPLTRTTSRAASIHESLYSNRVSFLKRAARRAG-----FG-DKRVRMSQGE	979
Ustilago maydis	---------AAAHESLSRTTSRADSMYSNRTSFLKRAQRKAG-----FGGEKKVHMSNTE	944
Puccinia graminis	LKLVDEATGIPLTR----TASRHESLYSNHATWIKRAQRRVG-----LK-KGT-TIDPTE	969

Species	Sequence	Position
Neurospora crassa	-----HEKSQ----------------------	920
Sporothrix schenckii	-----HEKSQ----------------------	929
Histoplasma capsulatum	--------------------------------	916
Coccidioides immites	--------------------------------	935
Blastomyces dermatitidis	--------------------------------	929
Acremonium chrysogenum	-----HEKSQ----------------------	929
Trichophyton rubrum	EERINEHNPYSP---KRAILIRRLYSLFSCLV	988
Candida glabrata	--------------------------------	902
Candida auris	--------------------------------	899
Candida albicans	--------------------------------	895
Pneumocystis jirovecii	--------------------------------	931
Aspergillus fumigatus	-----DE-------------------------	928
Talaromyces marneffei	-----DE-------------------------	995
Syncephalastrum racemosum	-----S---------------VVSF-------	883
Rhizopus stolonifera	MRRLSSVQASQASRVLSSGNN-----------	951
Lichtheimia corymbifera	LRRFSSIQTSQAAQVLNSGSNNRS--------	978
Cryptococcus gattii	LQRFSSIQAQQSGAALTRAHSRPAA-------	995
Cryptococcus neoformans	LQRFSSIQAQQSGAALTRAHSRPAA-------	997
Claviceps purpurea	-----HEKSA----------------------	925
Colletotrichum gloeosphorioides	EVSVKKDVSSQP---SSSALGALSWAA-----	948
Magnaporthe oryzae	-----HEKSQ----------------------	926
Fusarium oxysporum	-----HEKSQ----------------------	923
Botrytis cinerea	-----HEKSA----------------------	944
Fusarium graminearum	-----HEKSQ----------------------	922
Aspergillus niger	--------------------------------	928
Blumeria graminis	-----DD-------------------------	976
Sclerotinia slerotiorum	-----DD-------------------------	985
Mycosphaerella graminicola	-----DD-------------------------	007
Cochliobolus heterostrophus	-----DD-------------------------	971
Rhizoctonia solani	LQRFSSIQAAQTGATLARNPSRPH--------	003
Ustilago maydis	LQRLGSIQAQEASRRLSRTQ------------	964
Puccinia graminis	LHRVGSHQARATGAALRRLILGNQWRTFCVKF	001

Table S5: Alignment of *Neurospora crassa* Pma1 with plasma membrane proton pumps of selected plants.
Sequences are sorted in decending order according to their identity with Pma1. Accession codes: *N. crassa* (sp|P07038), *Coffea eugenioides* (XP_027176212.1), *Spinacia oleracea* (XP_021865157.1), *Cucumis sativus* (XP_004152192.1), *Hordeum vulgare* (KAE8805265.1:29-858), *Jatropha curcas* (XP_012068768.1:37-846), *Triticum aestivum* (P83970.1:29-858), *Ananas comosus* (XP_020090190.1), *Chenopodium quinoa* (XP_021755229.1:32-855), *Carica papaya* (XP_021899224.1), *Ricinus communis* (XP_015572514.1:34-875), *Punica granatum* (XP_031378860.1), *Nicotiana tabacum* (NP_001312285.1), *Brassica napus* (XP_022556197.1), *Gossypium australe* (KAA3489374.1:33-874), *Arabidopsis thaliana* (NP_194748.1), *Manihot esculenta* (XP_021598156.1:34-875), *Malus domestica* (XP_008372282.1:35-876), *Camellia sinensis* (XP_028098451.1:32-870), *Zea mays* (AQK46772.1:25-866), *Theobroma cacao* (EOY29625.1), *Sesamum indicum* (XP_011084025.1), *Hevea brasiliensis* (XP_021654241.1:37-846), *Glycine max* (XP_003549696.1:32-903)

Neurospora crassa	MADHSASGAPALSTNIESGKFDEKAAEAAAYQPKPKVEDDEDEDIDALIEDLESHDGHDA	60
Coffea eugenioides	--MASDLS	06
Spinacia oleracea	--MASKLE	06
Cucumis sativus	---MTDIS	05
Hordeum vulgare	--	00
Jatropha curcas	--	00
Triticum aestivum	--	00
Ananas comosus	--MGGDNALS	08
Chenopodium quinoa	--	00
Carica papaya	--MASDIS	06
Ricinus communis	--	00
Punica granatum	---MASSNTDLT	09
Nicotiana tabacum	--MASNLS	06
Brassica napus	--MADIEA	06
Gossypium australe	--	00
Arabidopsis thaliana	---MSS	03
Manihot esculenta	--	00
Malus domestica	--	00
Camellia sinensis	--	00
Zea mays	--	00
Theobroma cacao	--MASDGDTS	08
Sesamum indicum	--MASNIS	06
Hevea brasiliensis	--	00
Glycine max	--	00

Neurospora crassa	EEEEEEATPGGGRVVPEDM--LQTDTRVGLTSEEVVQRRRKYGLNQMKEEKENHFLKFLG	118
Coffea eugenioides	LEEIKNEQVDLENIPVEEVFKQLKCSREGLSEEEGAKRLQIFGPNKLEEKKESKLLKFLG	066
Spinacia oleracea	EACEAAEDLDLENVPIEEVFVSLQCTRNGLTSQEVEQRILKYGYNKLEEKKENKILKFLG	066
Cucumis sativus	LQEIKNENIDLERIPVEEVFEQLKCSREGLSSEEGRQRLQLFGPNKLEEKKESKFLKFLG	065
Hordeum vulgare	-------------------------TRQGLTSDEGAQRVEIFGLNKLEEKKESKVLKFLG	035
Jatropha curcas	-------------------------SREGLSSEDAEVRLKIFGPNKLEEKPENKFLKFLS	035
Triticum aestivum	-------------------------TRQGLTSDEGAQRVEIFGLNKLEEKKESKVLKFLG	035
Ananas comosus	LEEIKNENVDLERIPVDEVFQQLKCSREGLSTEEGNQRIQIFGPNKLEEKKESKFLKFLG	068
Chenopodium quinoa	-------------------------TRNGLSSDEVEQRIAKYGYNKLEEKKESKILKFLG	035
Carica papaya	LEEIKNENVDLERIPVDEVFEQLKCTREGLTSAEGEKRLQIFGPNKLEEKKENKVLKFLG	066
Ricinus communis	-------------------------SREGLSSEEGTNRLQVFGPNKLEEKKESKILKFLG	035
Punica granatum	LEEIKNENIDLERIPVDEVFEQLKCTREGLTTEEGQKRLQIFGQNKLEEKKESKLLKFLG	069
Nicotiana tabacum	LEDIKNEQVDLENIPVEEVFQQLKCSKEGLSSEEGKTRLQIFGPNKLEEKKENKFLKFLG	066
Brassica napus	LKAITTESIDLENVPVEEVFQHLKCTKEGLTSKEVQERLTLFGYNKLEEKKESKILKFLG	066
Gossypium australe	-------------------------TKEGLTTEEGLKRLQIFGPNKLEEKSESKVLKFLG	035
Arabidopsis thaliana	LEDIKNETVDLEKIPIEEVFQQLKCSREGLTTQEGEDRIQIFGPNKLEEKKESKLLKFLG	063
Manihot esculenta	-------------------------TREGLTSEEGANRLQVFGPNKLEEKKESKLLKFLG	035
Malus domestica	-------------------------SREGLSSEEGGQRLEIFGPNKLEEKKESKFLKFLG	035
Camellia sinensis	-------------------------TRDGLSSEEGENRLQIFGPNKLEEKKECKLLKFLG	035
Zea mays	-------------------------TREGLSSSEGQQRLEIFGPNRLEEKKESKILKFLG	035
Theobroma cacao	LEQIKNETVDLERIPVEEVFEQLKCTREGLTTAEGEKRLEIFGPNKLEEKKECKFLKFLG	068
Sesamum indicum	LEDIKNETVDLENIPIEEVFQELKCSRDGLTSDEGAKRLQIFGANKLEEKKESKFLKFLG	066
Hevea brasiliensis	-------------------------SRAGLSTEDAEVRLKIFGLNKLEEKPENKFLKFLS	035
Glycine max	-------------------------TREGLTSAEGEKRLQIFGPNKLEEKKDSKLLKFLG	035

Neurospora crassa	FFVGPIQFVMEGAAVLAAGL-------EDWVDFGVICGLLLLNAVVGFVQEFQAGSIVDE	171
Coffea eugenioides	FMWNPLSWVMESAAVMAIVLANGGGKPPDWQDFVGIVVLLILNSTVSFIEENNAGNAAAA	126
Spinacia oleracea	FMWNPLSWVMEAAAIMAIALAHGGGKGPDYPDFIGIVVLLLVNSTISYIEENNAGNAAAA	126
Cucumis sativus	FMWNPLSWVMEAAAIMAIVLANGGGQPPDWQDFVGITALLIINSTISFIEENNAGNAAAA	125
Hordeum vulgare	FMWNPLSWVMEMAAIMAIALANGGGKPPDWQDFVGIIVLLVINSTISFIEENNAGNAAAA	095
Jatropha curcas	FMWNPLSWVMEAAAVMAIVLANGGGEGPDWQDFVGIICLLLINSTISFIEENNAGNAAAA	095
Triticum aestivum	FMWNPLSWVMEMAAIMAIALANGGGKPPDWQDFVGIIVLLVINSTISFIEENNAGNAAAA	095
Ananas comosus	FMWNPLSWVMEMAAVMAIALANGGGQAPDWQDFVGIIVLLVINSTISFIEENNAGNAAAA	128
Chenopodium quinoa	FMWNPLSWVMEIAALMAIALAHGGGKGPDYPDFIGIVVLLLVNSTISYIEENNAGNAAAA	095
Carica papaya	FMWNPLSWVMESAAIMAIVLANGGGKPPDWQDFVGITVLLIINSTISFIEENNAGNAAAA	126
Ricinus communis	FMWNPLSWVMEAAAIMAIALANGDGRPPDWQDFVGIVALLLINSTISFIEENNAGNAAAA	095
Punica granatum	FMWNPLSWVMECAAIMAIALANGGGKPPDWQDFIGIVVLLIINSTISFIEENNAGNAAAA	129
Nicotiana tabacum	FMWNPLSWVMESAAIMAIALANGGGKAPDWPDFVGITVLLVINSTISFIEENNAGNAAAA	126
Brassica napus	FMWNPLSWVMEAAALMAIGLAHGGGKPPDYHDFVGIVVLLLINSTISFVEENNAGNAAAA	126
Gossypium australe	FMWNPLSWVMEIAAVMAIALANGGGKPPDWQDFVGITVLLIINSTISFIEENNAGNAAAA	095
Arabidopsis thaliana	FMWNPLSWVMEMAAIMAIALANGDGRPPDWQDFVGIICLLVINSTISFIEENNAGNAAAA	123
Manihot esculenta	FMWNPLSWVMEAAALMAIVLANGDGRPPDWQDFVGIIALLFINSTISFIEENNAGNAAAA	095
Malus domestica	FMWNPLSWVMEAAAIMAIALANGDGKPPDWQDFVGILCLLVINSSISFIEENNAGNAAAA	095
Camellia sinensis	FMWNPLSWVMECAAIMAIVLANGGGKPPDWQDFTGIVVLLIINSTISFIEENNAGNAAAA	095
Zea mays	FMWNPLSWVMEMAAVMAIALANGGGKPPDWEDFVGIIVLLVINSTISFIEENNAGNAAAA	095
Theobroma cacao	FMWNPLSWVMEAAAIMAIVLANGGGKPPDWQDFVGIVALLFINSTISFIEENNAGNAAAA	128
Sesamum indicum	FMWNPLSWVMELAAIMAIVLANGGGKPPDYPDFIGIVVLLVINSTISFIEENNAGNAAAA	126
Hevea brasiliensis	FMWNPLSWVMEAAAVMAIVLANGGGEGPDWQDFVGIICLLLINSTISFIEENNAGNAAAA	095
Glycine max	FMWNPLSWVMEVAAIMAIVMANGGGKPPDWQDFVGIVVLLIINSTISFIEENNAGNAAAA	095

Table S5: continued

Neurospora crassa	LKKTLALKAVVLRDGTLKEIEAPEVVPGDILQVEEGTIIPADGRIVTDDAFLQVDQSALT	231
Coffea eugenioides	LMAGLAPKTKVLRDGKWTEQDAAILVPGDLISVKLGDIIPADARLLEGDA-LKIDQSALT	185
Spinacia oleracea	LMARLAPKAKVQRDGSWNEEDAARLVPGDIISIKLGDIVPADARLLNGDP-LKIDQSALT	185
Cucumis sativus	LMAGLAPKTKVLRDGRWCEEEAAILVPGDVISVKLGDIIPADARLLEGDP-LKIDQSALT	184
Hordeum vulgare	LMANLAPKTKVLRDGRWGEQEASILVPGDIVSIKLGDIVPADARLLEGDP-LKIDQSGLT	154
Jatropha curcas	LMAHLAPKTKVLRDGRWKEEDAAILVPGDIISIKLGDIIPADARLLEGDP-LKIDQSALT	154
Triticum aestivum	LMANLAPKTKVLRDGRWGEQEASILVPGDIVSIKLGDIVPADARLLEGDP-LKIDQSGLT	154
Ananas comosus	LMANLAPKTKVLRDGQWSEQEAAILVPGDIISIKLGDIVPADARLLEGDP-LKVDQSALT	187
Chenopodium quinoa	LMARLAPKAKVLRNGEWNEDDAIRLVPGDIISIKLGDIVPADARLLNGDP-LKIDQSALT	154
Carica papaya	LMAGLAPKTKVLRDGKWSEQEAAILVPGDVISIKLGDIVPADARLLEGDP-LKIDQSALT	185
Ricinus communis	LMAGLAPKTKVLRDGRWTEQEAAILVPGDIISIKLGDIIPADARLLEGDP-LKVDQSALT	154
Punica granatum	LMAGLAPKTKVLRDGKWSEQEAAILVPGDVISVKLGDIIPADARLLEGDP-LKIDQSALT	188
Nicotiana tabacum	LMANLAPKTKILRDGKWSEEDASILVPGDLISIKLGDIVPADARLLEGDP-LKIDQAALT	185
Brassica napus	LMAQLAPKAKAIRDKKWNEIDASELVPGDIVSIKLGDIIPADARLLEGDP-LKIDQSALT	185
Gossypium australe	LMAGLAPKTKVLRDGKWCEQEAAILVPGDIISIKLGDIIPADARLLEGDA-LKVDQSALT	154
Arabidopsis thaliana	LMAGLAPKTKVLRDGKWSEQEAAILVPGDIVSIKLGDIIPADARLLEGDP-LKVDQSALT	182
Manihot esculenta	LMAGLAPKTKVLRDGRWTEQEAAILVPGDIISIKLGDIIPADARLLEGDP-LKVDQSALT	154
Malus domestica	LMAGLAPKTKVLRDGKWSEQDAAILVPGDIISIKLGDIVPADARLLEGDP-LKIDQSALT	154
Camellia sinensis	LMAGLAPKTKVLRDGKWSEQEASILVPGDLISVKLGDIIPADARLLEGDP-LKIDQSALT	154
Zea mays	LMANLAPKTKVLRDGRWGEQEAAILVPGDIVSIKLGDIVPADARLLEGDP-LKVDQSALT	154
Theobroma cacao	LMAGLAPKTKVLRDGKWGEQDAAILVPGDVISVKLGDIIPADARLLEGDA-LKIDQSALT	187
Sesamum indicum	LMAGLAPKTKVLRDGKWSEQDASILVPGDLISVKLGDIIPADARLLEGDP-LKIDQSALT	185
Hevea brasiliensis	LMAHLAPKTKVLRDGQWQEQDAAILVPGDIISIKLGDIIPADARLLKGDP-LKIDQSALT	154
Glycine max	LMAGLAPKTKVLRDGKWSEEEAALLVPGDLISIKLGDIVPADARLLEGDP-LKIDQSALT	154

Neurospora crassa	GESLAVDKHKGDQVFASSAVKRGEAFVVITATGDNTFVGRAAALVNAASGGSGHFTEVLN	291
Coffea eugenioides	GESLPVTKNPGDEVFSGSTCKQGEIEAVVIATGVHTFFGKAAHLVDST-NHVGHFQKVLT	244
Spinacia oleracea	GESLPVTKHPGSQVYSGSTCKQGEIEAVVIATGINTFFGKAAHLVENT-HHVGHFQKVLT	244
Cucumis sativus	GESLPVTRSSGDEVFSGSTVKQGEIEAVVIATGVHTFFGKAAHLVDST-NQVGHFQKVLT	243
Hordeum vulgare	GESLPVTKNPGDEVFSGSTCKQGEIEAVVIATGVHTFFGKAAHLVDST-NQVGHFQQVLT	213
Jatropha curcas	GESLAVTKRTGDEVFSGSTCKHGEIEAVVIATGVNTFFGKAAHLVDST-IVVGHFQQVLT	213
Triticum aestivum	GESLPVTKNPGDEVFSGSTCKQGEIEAVVIATGVHTFFGKAAHLVDST-NQVGHFQQVLT	213
Ananas comosus	GESLPVTKNPGDEVFSGSTCKQGEIEAVVIATGVHTFFGKAAHLVDST-NQVGHFQKVLT	246
Chenopodium quinoa	GESLPVTKHPGNQVYSGSTCKQGEIEAVVIATGINTFFGKAAHLVENT-NHVGHFQKVLT	213
Carica papaya	GESLPVTKEPGDEVFSGSTCKQGEIEAVVIATGVHTFFGKAAHLVDST-NNVGHFQKVLT	244
Ricinus communis	GESLPVTKNPSDEVFSGSTCKQGEIEAVVIATGVHTFFGKAAHLVDST-NQVGHFQKVLT	213
Punica granatum	GESLPVTKHPGDEVFSGSTCKQGEIEAVVIATGVHTFFGKAAHLVDST-NQVGHFQKVLT	247
Nicotiana tabacum	GESLPVTKFPGAEVFSGSTVKQGEIEAVVIATGVHTFFGKAAHLVDST-NQVGHFQKVLT	244
Brassica napus	GESLPVTKNPGSSVFSGSTCKQGEIEAVVIATGVHTFFGKAAHLVDST-THVGHFQKVLT	244
Gossypium australe	GESLPVNKNPGDGVYSGSTVKQGELEAVVIATGVHTFFGKAAHLVDST-NNVGHFQKVLT	213
Arabidopsis thaliana	GESLPVTKHPGQEVFSGSTCKQGEIEAVVIATGVHTFFGKAAHLVDST-NQVGHFQKVLT	241
Manihot esculenta	GESLPVTKNPSDEVFSGSTCKQGEIEAVVIATGVHTFFGKAAHLVDST-NQVGHFQKVLT	213
Malus domestica	GESLPVTKHPGDEVFSGSTCKQGEIEAVVIATGVHTFFGKAAHLVDST-NQVGHFQKVLT	213
Camellia sinensis	GESLPVTKHPGDGVFSGSTCKQGEIEAVVIATGVHTFFGKAAHLVDST-NQVGHFQKVLT	213
Zea mays	GESLPVTKGPGDEVFSGSTCKQGEIEAVVIATGVHTFFGKAAHLVDST-NQVGHFQQVLT	213
Theobroma cacao	GESLPVTKNPGDEVFSGSTCKQGEIEAVVIATGVHTFFGKAAHLVDST-NNVGHFQKVLT	246
Sesamum indicum	GESLAVTKHPGDEVFSGSTCKQGEIEAVVIATGVHTFFGKAAHLVDST-NNVGHFQKVLT	244
Hevea brasiliensis	GESLPVTKRTGDEVFSGSTCKHGEIEAVVIATGVNTFFGKAAHLVDST-EVVGHFQKVLT	213
Glycine max	GESLPVTKNPGSEVFSGSTCKQGEIEAIVIATGVHTFFGKAAHLVDST-NNVGHFQKVLT	213

Neurospora crassa	GIGTILLILVIFTLLIVWVSSFY-RSNPIVQILEFTLAITIIGVPVGLPAVVTTTMAVGA	350
Coffea eugenioides	AIGNFCICSIGLGMVIEVVVMYPVQHRKYREGIDNLLVLLIGGIPIAMPTVLSVTMAIGS	304
Spinacia oleracea	SIGNFCICSIAVGMLIEIIVIYALHQRPYRVGIDNLLVLLIGGIPIAMPTVLSVTMAIGS	304
Cucumis sativus	AIGNFCICSIAMGMVIEIVVMYPIQHRRYRDGINNLLVLLIGGIPIAMPTVLSVTMAIGS	303
Hordeum vulgare	AIGNFCIISIAVGIVIEIIVMFPIQRRKYRAGIENLLVLLIGGIPIAMPTVLSVTMAIGS	273
Jatropha curcas	SIGNFCICSIAVGMVLEIIVMFPIQHRSYRDGINNLLVLLIGGIPIAMPTVLSVTLAIGS	273
Triticum aestivum	AIGNFCIVSIAVGIVIEIIVMFPIQRRKYRAGIENLLVLLIGGIPIAMPTVLSVTMAIGS	273
Ananas comosus	AIGNFCICSIAIGIVVEIIVMFPIQHRAYRSGIDNLLVLLIGGIPIAMPTVLSVTMAIGS	306
Chenopodium quinoa	SIGNFCICSIAVGMLIEIIVIYALHQRPYRVGIDNLLVLLIGGIPIAMPTVLSVTMAIGS	273
Carica papaya	AIGNFCICSIGIGMLIEIVIMYPIQQRKYRDGIDNLLVLLIGGIPIAMPTVLSVTMAIGS	304
Ricinus communis	AIGNFCICSIAVGIIIELIVMYPIQHRKYRDGIDNLLVLLIGGIPIAMPTVLSVTMAIGS	273
Punica granatum	AIGNFCICSIAIGMLVEIIVMYPIQHRRYREGIDNLLVLLIGGIPIAMPTVLSVTMAIGS	307
Nicotiana tabacum	AIGNFCICSIAVGMVIEIVVMYPIQRRKYRDGIDNLLVLLIGGIPIAMPTVLSVTMAIGS	304
Brassica napus	SIGNFCICTIAVGMAIEIVVIYGLQKRAYRVGIDNLLVLLIGGIPIAMPTVLSVTMAIGA	304
Gossypium australe	AIGNFCICSIAVGMLVEIVVMYPIQRRKYRDGIDNLLVLLIGGIPIAMPTVLSVTMAIGS	273
Arabidopsis thaliana	AIGNFCICSIAIGMVIEIIVMYPIQRRKYRDGIDNLLVLLIGGIPIAMPTVLSVTMAIGS	301
Manihot esculenta	AIGNFCICSIAVGIVIEIIVMYPIQHRKYRQGIDNLLVLLIGGIPIAMPTVLSVTMAIGS	273
Malus domestica	AIGNFCICSIAIGMLIEIVVMYPIQHRKYRSGIDNLLVLLIGGIPIAMPTVLSVTIAIGS	273
Camellia sinensis	SIGNFCICSIGVGMVIEIIVMYPIQNRKYREGIDNLLVLLIGGIPIAMPTVLSVTMAIGS	273
Zea mays	AIGNFCICSIAVGIVVEIIVMFPIQHRRYRSGIENLLVLLIGGIPIAMPTVLSVTMAIGS	273
Theobroma cacao	AIGNFCICSIAVGMLIEIIVMYPIQHRRYRDGIDNLLVLLIGGIPIAMPTVLSVTMAIGS	306
Sesamum indicum	AIGNFCICSIALGMIIEIIVMYPIQHRKYRDGIDNLLVLLIGGIPIAMPTVLSVTMAIGS	304
Hevea brasiliensis	SIGNFCICSIAVGMILEIIVMFPIQHRSYRDGINNLLVLLIGGIPIAMPTVLSVTLAIGS	273
Glycine max	SIGNFCICSIAVGMLIEIIVMFPIQQRAYRDGIDNLLVLLIGGIPIAMPTVLSVTMAIGS	273

Table S5: continued

Neurospora crassa	AYLAKKKAIVQKLSAIESLAGVEILCSDKTGTLTKNKLSLHDPYT---VAGVDPEDLMLT	407
Coffea eugenioides	HRLSEQGAITKRMTAIEEMAGMDVLCSDKTGTLTLNKLTVDKQMIEVFPKNMDKDSVVLY	364
Spinacia oleracea	HRLAQQGAITKRMTAIEEMAGMDVLCSDKTGTLTLNKLTVDKNLIEVFSKGVDKDMVVLM	364
Cucumis sativus	HRLSQQGAITKRMTAIEEMAGMDVLCSDKTGTLTLNKLTVDKSMIEVFVRDVDKDNLVLL	363
Hordeum vulgare	HKLSQQGAITKRMTAIEELAGMDVLCSDKTGTLTLNKLSVDKNLVEVFAKGVDKEHVLLL	333
Jatropha curcas	HRLSQQGAITKRMTAIEEMAGMDVLCSDKTGTLTLNRLTVDRNLIEVFNKEMDKDMIVLL	333
Triticum aestivum	HKLSQQGAITKRMTAIEELAGMDVLCSDKTGTLTLNKLSVDKNLVEVFAKGVDKEHVLLL	333
Ananas comosus	HKLSQQGAITKRMTAIEEMAGMDVLCSDKTGTLTLNKLSVDRNLVEVFAKGVDKDHVLLL	366
Chenopodium quinoa	HRLAQQGAITKRMTAIEEMAGMDVLCSDKTGTLTLNKLTVDKNLIEVFAKGVDKDMVVLL	333
Carica papaya	HRLSEQGAITKRMTAIEEMAGMDVLCSDKTGTLTLNKLTVDKSLIEVFPKGIDKDTVVLI	364
Ricinus communis	HRLSQQGAITKRMTAIEEMAGMDVLCSDKTGTLTLNKLTVDRTLIEVFVKGVDKEYVILL	333
Punica granatum	HRLSEQGAITKRMTAIEEMAGMDVLCSDKTGTLTLNKLTVDKSLIEVFPKDMDKDDLMLL	367
Nicotiana tabacum	HRLAQQGAITKRMTAIEEMAGMDVLCSDKTGTLTLNKLTVDKNLIEVFPKDADKDTVMLL	364
Brassica napus	HRLSQQGAITKRMTAIEEMAGMDVLCSDKTGTLTLNKLSVDKNLIEIFKKGIDKDMAVLM	364
Gossypium australe	HRLSQQGAITKRMTAIEEMAGMDVLCSDKTGTLTLNKLTVDKSMVEVFTKDVDKEMLLLL	333
Arabidopsis thaliana	HRLSQQGAITKRMTAIEEMAGMDVLCSDKTGTLTLNKLSVDKNLVEVFCKGVEKDQVLLF	361
Manihot esculenta	HRLSQQGAITKRMTAIEEMAGMDVLCSDKTGTLTLNKLTVDRTLIEVFAKGVDKEYVILL	333
Malus domestica	HRLSQQGAITKRMTAIEELAGMDVLCSDKTGTLTLNKLSVDKNLIEVFAKGVENEHVILL	333
Camellia sinensis	HRLSQQGAITKRMTAIEEMAGMDVLCSDKTGTLTLNKLTVDRTLIEVFPKDVDKDIVVLL	333
Zea mays	HKLSQQGAITKRMTAIEEMAGMDVLCSDKTGTLTLNKLSVDKNLVEVFCKGVDKDHVLLL	333
Theobroma cacao	HRLSEQGAITKRMTAIEEMAGMDVLCSDKTGTLTLNKLTVDRSLIEVFASGIDKDTLMLL	366
Sesamum indicum	HRLSQQGAITKRMTAIEEMAGMDVLCSDKTGTLTLNKLTVDKNLIEVFPKNADKDTVVLL	364
Hevea brasiliensis	HRLSQQGAITKRMTAIEEMAGMDVLCSDKTGTLTLNRLTVDRNLIEVFNKDMDKEIIVLL	333
Glycine max	HRLSEQGAITKRMTAIEEMAGMDVLCSDKTGTLTLNKLTVDKSLIEVFPTGMDRDTLVLY	333
Neurospora crassa	ACLAASRKKKGIDAIDKAFLKSLKYYPRAKSVLSKYKVLQFHPFDPVSKKVVAVVESPQG	467
Coffea eugenioides	AARA--SRTENQDAIDASIVNMLS---DPKEARAGITELHFLPFNPVEKRTAITYIDSNG	419
Spinacia oleracea	SARA--SRIENQDAIDGAIVSMLS---DPKQARAGIKEVHFLPFNPTDKRTALTYTDEAG	419
Cucumis sativus	GARA--SRVENQDAIDACIVGMLG---DPKEAREGIKEVHFLPFNPVDKRTAITFIDNDG	418
Hordeum vulgare	AARA--SRVENQDAIDACMVGMLA---DPKEARAGIREVHFLPFNPTDKRTALTYIDAEG	388
Jatropha curcas	AARA--SRLENQDAIDAAIVNMLA---DPKEARANIKEVHFLPFNPVDKRTAITYIDSDG	388
Triticum aestivum	AARA--SRVENQDAIDACMVGMLA---DPKEARAGIREVHFLPFNPTDKRTALTYIDAEG	388
Ananas comosus	AARA--SRTENQDAIDAAMVGMLA---DPKEARAGIREIHFFPFNPVDKRTALTYIDADG	421
Chenopodium quinoa	AARA--SRTENQDAIDGAIVSMLP---DSKQAREGIKEVHFLPFNPTDKRTALTYIDEAG	388
Carica papaya	AARA--SRTENQDAIDASIVGMLS---DPKEARAGISEQHFLPFNPVDKRTAITYIDNKG	419
Ricinus communis	AARA--SRTENQDAIDAAIVGMLA---DPKEARAGIRELHFLPFNPVDKRTALTYIDSDG	388
Punica granatum	AARA--SRVENQDAIDASIVGMLN---DPKEARAGITEVHFLPFNPVDKRTAITYIDSNG	422
Nicotiana tabacum	AARA--SRVENQDAIDACIVNMLG---DPKEARAGIQEVHFLPFNPVEKRTAITYIDDKG	419
Brassica napus	AARA--ARLENQDAIDTAIVSMLS---DPKEARAGIKELHFLPFSPANRRTALTYLDGEG	419
Gossypium australe	AARA--SRVENQDAIDACIVGMLG---DPKEAREGITEVHFFPFNPVDKRTAMTYIESDG	388
Arabidopsis thaliana	AAMA--SRVENQDAIDAAMVGMLA---DPKEARAGIREVHFLPFNPVDKRTALTYIDGSG	416
Manihot esculenta	AARA--SRTENQDAIDAAIVGMLA---DPKEARAGIREVHFLPFNPVDKRTALTYIDSNG	388
Malus domestica	AARS--SRTENQDAIDAAMVGMLA---DPKEARAGIREVHFLPFNPVDKRTALTYIDNDG	388
Camellia sinensis	AARA--SRIENQDAIDASIVGMLG---DPKEARAGITEVHFLPFNPVEKRTAITYFDSND	388
Zea mays	AARA--SRTENQDAIDAAMVGMLA---DPKEARAGIREIHFLPFNPVDKRTALTYIDADG	388
Theobroma cacao	AARA--SRVENQDAIDASIVGMLG---DPKEARAGITELHFFPFNPVDKRTAITYIDSDG	421
Sesamum indicum	AARA--SRIENQDAIDASIVNMLG---DPKEARAGIIEVHFLPFNPVEKRTAITYYDSNG	419
Hevea brasiliensis	AARA--SRLENQDAIDAAIVNMLA---DPKEARANIKEVHFLPFNPVDKRTAITYIDSDG	388
Glycine max	AARA--SRIENQDAIDASIVGMLG---DPKEARAGITEVHFLPFNPVDKRTAITYIDGQG	388
Neurospora crassa	ERITCVKGAPLFVLKTVEEDHPIPEEVDQAYKNKVAEFATRGFRSLGVARKR--------	519
Coffea eugenioides	DWHRSSKGAPEQIIDLCELKG----EVRKKAHDIIDNFANRGLRSLGVARQAVPEKNKES	475
Spinacia oleracea	KMHRVSKGAPEEILALAHNKT----EIERRVHTVIDNFANRGLRSLGVARQEVPEGTQES	475
Cucumis sativus	SWHRVSKGAPEQIIELCDLRE----DIKKKAHSIIDKFADRGLRSLAVCRQTVPEKTKES	474
Hordeum vulgare	NWHRASKGAPEQIITLCNCKE----DVKRKVHSVIEKYAERGLRSLAVARQEVPEKSKDS	444
Jatropha curcas	NWYRASKGAPEQILHMCQEKD----KIAEKVHVIIDKFAERGLRSLGVAFQEVPEKRKDS	444
Triticum aestivum	NWHRASKGAPEQIITLCNCKE----DVKRKVHSVIEKYAERGLRSLAVARQEVPEKSKDS	444
Ananas comosus	NWHRVSKGAPEQIMNLCNCKE----DVKRRAHNVIDKFAERGLRSLGVARQEVPEKSKES	477
Chenopodium quinoa	KMHRVSKGAPEQILELAHNQS----AIERRVHTVIDNFANRGLRSLGVARQEVPEGTQES	444
Carica papaya	DWHRCSKGAPEQIIDLCELKG----ETRKKAHEVIDNYAQRGLRSLGVAQQTIPEKTKES	475
Ricinus communis	NWHRASKGAPEQILTLCNSKE----DVKKKVHAVIDKFAERGLRSLAVARQEVPERSKES	444
Punica granatum	DWHRCSKGAPEQIIELCGLKG----EACKKAHKIIDNFAERGLRSLGVARQTVLEKTKES	478
Nicotiana tabacum	NWHRCSKGAPEQIIELCELKG----DIRKKSLDIIDSYANRGLRSLAVARQTVPEKSKES	475
Brassica napus	KMHRVSKGAPEEILDMAHNML----EIKEKVHGKIDKFAERGLRSLGLAYQQVPDGDVKG	475
Gossypium australe	SWHRVSKGAPEQIISLCDLRD----DVKKKAHDIIDKFADRGLRSLGVARQTVPEKTKDS	444
Arabidopsis thaliana	NWHRVSKGAPEQILELAKASN----DLSKKVLSIIDKYAERGLRSLAVARQVVPEKTKES	472
Manihot esculenta	NWHRASKGAPEQILALCNSKE----DVRKKVHAVIDKFAERGLRSLAVARQEVPEKTKDS	444
Malus domestica	NWHRASKGAPEQILELCNCKE----DFKKKAFAIIDKYAERGLRSLAVARQEVPEKAKES	444
Camellia sinensis	NWYRTSKGAPEQIIDLCDLKG----DVRKKALEIIDNFANRGLRSLGVARQTVPEKTKES	444
Zea mays	HWHRVSKGAPEQILDLCHCKE----DLRRKVHGIIDKYAERGLRSLAVARQEVPERNKES	444
Theobroma cacao	EWHRCSKGAPEQIIDLCGLTG----ELRKKALGVIDNFADRGLRSLAVARQTIPEKTKES	477
Sesamum indicum	NWHRCSKGAPEQIIELCELKG----ETLKRAHEIIDNFANRGLRSLGVARQTIPEKNKES	475
Hevea brasiliensis	NWYRASKGAPEQILNMCQEKD----QIAGKVHTIIDKFAERGLRSLGVAFQEVPEKTKDS	444
Glycine max	NWHRSSKGAPEQIIELCELKG----EVLKKAHKVIDEYANRGLRSLGVSRQTVSEKNKES	444

Table S5: continued

Neurospora crassa	GEGSWEILGIMPCMDPPRHDTYKTVCEAKTLGLSIKMLTGDAVGIARETSRQLGLGTNIY	579
Coffea eugenioides	AGGPWEFIGLLPLFDPPRHDSAETIKKALDLGVNVKMITGDQLAIGKETGRRLGMGTNMY	535
Spinacia oleracea	LGGPWEFVALLPLFDPPRHDSAETIRRALDLGVSVKMITGDQLAIAKETGRRLGMGTNMY	535
Cucumis sativus	AGGPWQFVGLLPLFDPPRHDSAETIRRALNLGVNVKMITGDQLAIGKETGRRLGMGTNMY	534
Hordeum vulgare	AGGPWQFIGLLPLFDPPRHDSAETIRKALVLGVNVKMITGDQLAIGKETGRRLGMGTNMY	504
Jatropha curcas	PGGPWTFCGLLPLFDPPRHDSAETIRRALNLGVCVKMITGDQLAIAKETGRRLGMGTNMY	504
Triticum aestivum	PGGPWQFIGLLPLFDPPRHDSAETIRKALVLGVNVKMITGDQLAIGKETGRRLGMGTNMY	504
Ananas comosus	PGAPWQFVGLLPLFDPPRHDSAETIRRALNLGVNVKMITGDQLAIAKETGRRLGMGTNMY	537
Chenopodium quinoa	PGGPWEFVALLPLFDPPRHDSAETIRKALDLGVSVKMITGDQLAIGKETGRRLGMGTNMY	504
Carica papaya	SGSPWEFVGLLPLFDPPRHDSAETIKRALDLGVNVKMITGDQLAIGKETGRRLGMGTNMY	535
Ricinus communis	PGGPWQLVGLLPLFDPPRHDSAETIRRALNLGVNVKMITGDQLAIAKETGRRLGMGTNMY	504
Punica granatum	EGEPWEFVGLLPLFDPPRHDSAETIRRALDLGVNVKMITGDQLAIGKETGRRLGMGTNMY	538
Nicotiana tabacum	DGSPWEFVGLLPLFDPPRHDSAETIRKALDLGVNVKMITGDQLAIGKETARRLGMGTNMY	535
Brassica napus	DGGPWDFVALLPLFDPPRHDSAQTIERALHLGVSVKMITGDQLAIAKETGRRLGMGTNMY	535
Gossypium australe	PGSPWEFVGLLPLFDPPRHDSAETIRRALHLGVNVKMITGDQLAIGKETGRRLGMGTNMY	504
Arabidopsis thaliana	PGAPWEFVGLLPLFDPPRHDSAETIRRALNLGVNVKMITGDQLAIGKETGRRLGMGTNMY	532
Manihot esculenta	PGGPWQFVGLLPLFDPPRHDSAETIRRALNLGVNVKMITGDQLAIAKETGRRLGMGTNMY	504
Malus domestica	AGGPWQLVGLLPLFDPPRHDSAETIRQALNLGVNVKMITGDQLAIAKETGRRLGMGTNMY	504
Camellia sinensis	AGGPWEFVGLLPLFDPPRHDSAETIRRALDLGVSVKMITGDQLAIGKETGRRLGMGTNMY	504
Zea mays	PGGPWQFVGLLPLFDPPRHDSAETIRKALVLGVNVKMITGDQLAIGKETGRRLGMGTNMY	504
Theobroma cacao	AGGPWEFVGLLPLFDPPRHDSAETIRRALDLGVNVKMITGDQLAIGKETGRRLGMGTNMY	537
Sesamum indicum	AGGPWEFVGLLPLFDPPRHDSAETIKKALDLGVNVKMITGDQLAIGKETGRRLGMGTNMY	535
Hevea brasiliensis	PGGPWTFCGLLPLFDPPRHDSAETIRRALNLGVGVKMITGDQLAIAKETGRRLGMGTNMY	504
Glycine max	AGESWEFLGLLPLFDPPRHDSAETIRRALDLGVNVKMITGDQLAIGKETGRRLGMGTNMY	504

Neurospora crassa	NAERLGLGGG--GDMPGSEVYDFVEAADGFAEVFPQHKYNVVEILQQRGYLVAMTGDGVN	637
Coffea eugenioides	PSSSLLGQSKDES-IASIPIDELIEKADGFAGVFPEHKYEIVKKLQERKHICGMTGDGVN	594
Spinacia oleracea	PSSAILGDNKDNRNPDMPPVQELIESADGFAGVFPEHKYNIVKILQERKHIVGMTGDGVN	595
Cucumis sativus	PSSSLLGQSKDES-IASLPVDELIEKADGFAGVFPEHKYEIVRRLQERKHICGMTGDGVN	593
Hordeum vulgare	PSSALLGQSKDGS-LESLPVDELIEKADGFAGVFPEHKYEIVKRLQEKKHIVGMTGDGVN	563
Jatropha curcas	PSSSLLGRDKDEN--EVLPVDELIEMADGFAGVFPEHKYEIVKILQEKKHVVGMTGDGVN	562
Triticum aestivum	PSSALLGQSKDGS-LESLPVDELIEKADGFAGVFPEHKYEIVKRLQEKKHIVGMTGDGVN	563
Ananas comosus	PSSALLGQDKDAS-LDALPVDELIEKADGFAGVFPEHKYEIVKRLQEKKHICGMTGDGVN	596
Chenopodium quinoa	PSSTILGDNKD-RSPDMPPIEELIENADGFAGVFPEHKYNIVKILQERKHIVGMTGDGVN	563
Carica papaya	PSSSLLGESKDES-IAGLPVDELIEKADGFAGVFPEHKYEIVKRLQDRKHICGMTGDGVN	594
Ricinus communis	PSSSLLGQDKDAS-IATLPVDELIEKADGFAGVFPEHKYEIVKRLQERKHICGMTGDGVN	563
Punica granatum	PSSSLLGESKDSA-VASLPVDELIEQADGFAGVFPEHKYEIVKRLQERKHICGMTGDGVN	597
Nicotiana tabacum	PSSALLGEHKDAA-IASIPVDELIEKADGFAGVFPEHKYEIVKKLQERNHICGMTGDGVN	594
Brassica napus	PSSSLLSDNHTE----TISIDELIENADGFAGVFPEHKYEIVKRLQSRKHICGMTGDGVN	591
Gossypium australe	PSSALLGHNKDEK-VDTIDVDELIEKADGFAGVFPEHKYEIVKRLQERNHICGMTGDGVN	563
Arabidopsis thaliana	PSSALLGTHKDAN-LASIPVEELIEKADGFAGVFPEHKYEIVKKLQERKHIVGMTGDGVN	591
Manihot esculenta	PSSSLLGQDKDAS-IAALPVDELIEKADGFAGVFPEHKYEIVKRLQERKHICGMTGDGVN	563
Malus domestica	PSASLLGQGKDAS-IAALPIEELIERADGFAGVFPEHKYEIVKKLQEKKHICGMTGDGVN	563
Camellia sinensis	PSSSLLGQSKDQS-ISSIPIEELIEKADGFAGVFPEHKYEIVKKLQERKHICGMTGDGVN	563
Zea mays	PSSALLGQNKDAT-LEALPVDELIEKADGFAGVFPEHKYEIVKRLQEKKHIVGMTGDGVN	563
Theobroma cacao	PSSSLLGQSKDEA-IAAIPVDELIEKADGFAGVFPEHKYEIVKRLQDRKHICGMTGDGVN	596
Sesamum indicum	PSSSLLGQSKDES-IASMPVEELIEKADGFAGVFPEHKYEIVKKLQERKHICGMTGDGVN	594
Hevea brasiliensis	PSSSLLGRDKDEY--EALPVDELIEKADGFAGVFPEHKYEIVKILQEKKHVVGMTGDGVN	562
Glycine max	PSSSLLGDSKDPA-IASIPVDELIEKADGFAGVFPEHKYEIVKRLQEMKHICGMTGDGVN	563

Neurospora crassa	DAPSLKKADTGIAVEGSSDAARSAADIVFLAPGLGAIIDALKTSRQIFHRMYAYVVYRIA	697
Coffea eugenioides	DAPALKKADIGIAVADATDAARSASDIVLTEPGLSVIVSAVLTSRAIFQRMKNYTIYAVS	654
Spinacia oleracea	DAPALKRADIGIAVDDSTDAARSASDIVLTEPGLSVIISAVLTSRSIFQRMKNYTIYAVS	655
Cucumis sativus	DAPALKKADIGIAVADATDAARGASDIVLTEPGLSVIISAVLTSRAIFQRMKNYTIYAVS	653
Hordeum vulgare	DAPALKKADIGIAVDDATDAARSASDIVLTEPGLSVIISAVLTSRCIFQRMKNYTIYAVS	623
Jatropha curcas	DAPALKKADIGIAVADSTDAARNAADLVLTEPGLSVIVSAVLTSRAIFQRMKNYTIYAVS	622
Triticum aestivum	DAPALKKADIGIAVDDATDAARSASDIVLTEPGLSVIISAVLTSRCIFQRMKNYTIYAVS	623
Ananas comosus	DAPALKKADIGIAVADATDAARGASDIVLTEPGLSVIISAVLTSRCIFQRMKNYTIYAVS	656
Chenopodium quinoa	DAPALKRADIGIAVDDSTDAARSASDIVLTEPGLSVIISAVLTSRSIFQRMKNYTIYAVS	623
Carica papaya	DAPALKKADIGIAVADATDAARGASDIVLTEPGLSVIVSAVLTSRAIFQRMKNYTIYAVS	654
Ricinus communis	DAPALKKADIGIAVADATDAARGASDIVLTEPGLSVIISAVLTSRAIFQRMKNYTIYAVS	623
Punica granatum	DAPALKKADIGIAVADATDAARSASDIVLTEPGLSVIVSAVLTSRAIFQRMKNYTIYAVS	657
Nicotiana tabacum	DAPALKKADIGIAVDDATDAARSASDIVLTEPGLSVIVSAVLTSRAIFQRMKNYTIYAVS	654
Brassica napus	DAPALKKADIGIAVDDATDAARSASDIVLTEPGLSVIVSAVLTSRAIFQRMKNYTIYAVS	651
Gossypium australe	DAPALKKADIGIAVDDATDAARSASDIVLTEPGLSVIVSAVLTSRAIFQRMKNYTIYAVS	623
Arabidopsis thaliana	DAPALKKADIGIAVADATDAARGASDIVLTEPGLSVIISAVLTSRAIFQRMKNYTIYAVS	651
Manihot esculenta	DAPALKKADIGIAVADATDAARGASDIVLTEPGLSVIISAVLTSRAIFQRMKNYTIYAVS	623
Malus domestica	DAPALKKADIGIAVADATDAARSASDIVLTEPGLSVIISAVLTSRAIFQRMKNYTIYAVS	623
Camellia sinensis	DAPALKKADIGIAVADATDAARSASDIVLTEPGLSVIVSAVLTSRAIFQRMKNYTIYAVS	623
Zea mays	DAPALKKADIGIAVADATDAARSASDIVLTEPGLSVIISAVLTSRCIFQRMKNYTIYAVS	623
Theobroma cacao	DAPALKKADIGIAVADATDAARSASDIVLTEPGLSVIVSAVLTSRAIFQRMKNYTIYAVS	656
Sesamum indicum	DAPALKKADIGIAVADATDAARGASDIVLTEPGLSVIISAVLTSRAIFQRMKNYTIYAVS	654
Hevea brasiliensis	DAPALKKADIGIAVADSTDAARSAADLVLTEPGLSVIVSAVLTSRAIFQRMKNYTIYAVS	622
Glycine max	DAPALKKADIGIAVADATDAARSASDIVLTEPGLSVIVSAVLTSRAIFQRMKNYTIYAVS	623

Table S5: continued

Neurospora crassa	LSIHLEIFLGLWIAILNRSLNIELVVFIAIFADVATLAIAYDNAPYSQTPVKWNLPKLWG	757
Coffea eugenioides	ITIRIVLGFLLIALIWKFDFSPFMVLIIAILNDGTIMTISKDRVKPSPVPDSWKLKEIFA	714
Spinacia oleracea	ITIRIVLGFMLLCVVWKFDFPPFMILVIAVLNDGTIMTISKDRVKPSPSPDRWNLKEIFA	715
Cucumis sativus	ITIRIVLGFMLIALIWKFDFSPFMVLIIAILNDGTIMTISKDRVKPSPLPDSWKLREIFA	713
Hordeum vulgare	ITIRIVLGFLLIALIWKFDFAPFMVLIIAILNDGTIMTISKDRVKPSPLPDSWKLNEIFA	683
Jatropha curcas	ITIRIVLGFVLLALIWEYDFPPFMVLIIAILNDGTIMTISQDRVKPSPRPDSWKLPEIFA	682
Triticum aestivum	ITIRIVLGFMLIALIWKFDFAPFMVLIIAILNDGTIMTISKDRVKPSPLPDSWKLNEIFA	683
Ananas comosus	ITIRIVLGFLLIALIWKFDFSPFMVLIIAILNDGTIMTISKDRVKPSPLPDSWKLKEIFA	716
Chenopodium quinoa	ITIRIVLGFMMLCVIWKFDFPPTMILVIAILNDGTIMTISKDRVKPSPTPDRWNLKEIFT	683
Carica papaya	ITIRIVLGFMLVALIWKFDFAPFMVLIIAILNDGTIMTISKDRVKPSPVPDSWKLKEIFA	714
Ricinus communis	ITIRIVFGFLFIALIWKFDFSPFMVLIIAILNDGTIMTISKDRVKPSPQPDSWKLKEIFS	683
Punica granatum	ITIRIVLGFLLIALIWKFDFSPFMVLIIAILNDGTIMTISKDRVKPSPVPDSWKLNEIFA	717
Nicotiana tabacum	ITIRVVMGFMLIALIWKFDFSPFMVLIIAILNDGTIMTISKDRVKPSPLPDSWKLKEIFA	714
Brassica napus	ITIRIVMCFMLLCVFWEFDFPPFMVLVIAILNDGTIMTISKDRVKPSPTPDCWKLKEIFA	711
Gossypium australe	ITIRIVLGFMLLALIWKFDFSPFMVLIIAILNDGTIMTISKDRVKPSPMPDSWKLKEIFA	683
Arabidopsis thaliana	ITIRIVFGFMLIALIWEFDFSAFMVLIIAILNDGTIMTISKDRVKPSPTPDSWKLKEIFA	711
Manihot esculenta	ITIRIVFGFMFIALIWKYDFAPFMVLIIAILNDGTIMTISKDRVKPSPQPDSWKLKEIFS	683
Malus domestica	ITIRIVFGFMFIALIWKFDFSPFMVLIIAILNDGTIMTISKDRVKPSPLPDSWKLKEIFA	683
Camellia sinensis	ITIRIVMGFMLIALIWKFDFSPFMVLVIAILNDGTIMTISKDRVKPSPVPDSWKLKEIFA	683
Zea mays	ITIRIVLGFMLIALIWQYDFSPFMVLIIAILNDGTIMTISKDRVKPSPLPDSWKLKEIFA	683
Theobroma cacao	ITIRIVMGFMLVALIWKFDFSPFMVLIIAILNDGTIMTISKDRVKPSPMPDSWKLNEIFA	716
Sesamum indicum	ITIRIVMGFMLIALIWKFDFSPFMVLIIAILNDGTIMTISKDRVKPSPLPDSWKLKEIFA	714
Hevea brasiliensis	ITIRIVLGFVLLALIWEYDFPPFMVLIIAILNDGTIMTISQDRVKPSPRPDSWKLPEIFA	682
Glycine max	ITIRIVLGFMLVALIWRFDFSPFMVLIIAILNDGTIMTISKDRVKPSPLPDSWKLKEIFA	683

Neurospora crassa	MSVLLGVVLAVGT----WITVTTMYAQGENG--GI-------------VQNFGNMDEVLF	798
Coffea eugenioides	TGVVLGTYLAVMTVVFFYLAADTDFFSNIFKVRSIRGH-------------PDELTAALY	761
Spinacia oleracea	TGIALGSYLATMTVVFYWAAYDTSFFAQKFGVRDMNQYKHNLSAPGVEKDLKERMASAVY	775
Cucumis sativus	TGVVLGSYLAVMTVVFFWIANATDFFSDKFGVHSIRGN-------------DGELTAAVY	760
Hordeum vulgare	TGVVLGTYLALMTVVFFWIIHRTDFFTNKFGVRSIREN-------------ETEKMSALY	730
Jatropha curcas	TGIVIGTYLALITVLFYWVVIDTDFFENTFNVRSLSSN-------------TEEVSSAVY	729
Triticum aestivum	TGVVLGTYLALVTVVFFWLIHKTDFFTNKFGVESIRNT-------------EFKEMSALY	730
Ananas comosus	TGVILGGYLALMTVIFFWAMKETNFFPDKFSVRSLRDN-------------DHEMMAALY	763
Chenopodium quinoa	TGIVLGSYLATMTIVFYWAAYDTSFFAEKFGVRDLNQYKHNLSAPGVEKDLKERMASAIY	743
Carica papaya	TGVVLGTYMALVTVLFFWLAHDTDFFSEKFGVRSIRES-------------EEELMAALY	761
Ricinus communis	TGVVLGGYLALMTVIFFWAMEETDFFSDKFGVRSLHNS-------------EGEMMAALY	730
Punica granatum	TGVVIGTYLALMSVLFFWLAHDTDFFSNVFGVRSISHN-------------VDELTAALY	764
Nicotiana tabacum	TGVVLGTYQAIMTVVFFYLAADTDFFSENFHVRSIRNS-------------PTELTAALY	761
Brassica napus	TGVVLGAYLAIMTVVFFWAAYETNFFPNIFDVRNFNQHHFNMRDKAVAANLNEQMASAVY	771
Gossypium australe	TGIVLGTYLACMTVVFFWAANDSDFFSDKFGVKSIRYS-------------QDELTAAVY	730
Arabidopsis thaliana	TGVVLGGYQAIMTVIFFWAAHKTDFFSDTFGVRSIRDN-------------NHELMGAVY	758
Manihot esculenta	TGVVLGGYLALMTVIFFWAMNNTNFFSDKFGVRSLHGR-------------EHEMMAALY	730
Malus domestica	TGIVLGGYLALMTVIFFWLIKETDFFSDQFGVRSLRET-------------PEELMAALY	730
Camellia sinensis	TGIVLGTYLALITVIFFWLAADTDFFSKTFGVRSIRGH-------------TDELTAALY	730
Zea mays	TGIVLGSYLALMTVIFFWAMHKTDFFSDKFGVRSIRDS-------------EHEMMSALY	730
Theobroma cacao	TGIVLGAYMAIISVLFFWLAHDTDFFTEKFGVRPIHDN-------------IDELTSALY	763
Sesamum indicum	TGIVLGTYMAIMTVVFFYLASDTDFFSDTFKVRSIRNN-------------FEELTAALY	761
Hevea brasiliensis	TGIVIGTYLALVTVLFYWLLIGTNFFERTFHVRSLSSN-------------SEEVSSAVY	729
Glycine max	TGVVLGAYMAIITVVFFFLVHDTDFFTRVFGVEPIVDS-------------EEQLNSALY	730

Neurospora crassa	LQISLTENWLIFITRANGPFWSSIPSWQLSGAIFLVDILATCFT---IWGWFEHSDTSIV	855
Coffea eugenioides	LQVSIISQALIFVTRSRSWSFVERPGLWLVTAFFIAQLVATFFAVYANWSFARIQGIGWG	821
Spinacia oleracea	LQVSIISQALIFVTRARGWSFTERPGFLLVVAFLIAQLIASIISAEASWQIAGIRKIGWG	835
Cucumis sativus	LQVSTVSQALIFVTRSRSWSFVERPGLLLVFAFFVAQLVATLIAVYANWGFAEMKGIGWG	820
Hordeum vulgare	LQVSIVSQALIFVTRSRSWSFVERPGFLLVIAFLLAQLVATLIAVYANWGFARISGIGWG	790
Jatropha curcas	LQVSIISQALIFVTRSQSWSFMERPGTLLMCAFIVAQLVATLIAVYAHISFAYISGIGWG	789
Triticum aestivum	LQVSIVSQALIFVTRSRSWSFVERPGFLLVTAFLLAQLVATLIAVYANWDFARIKGIGWG	790
Ananas comosus	LQVSIVSQALIFVTRSRSWSFVERPGLLLVSAFMVAQLVATLIAVYANWGFARIKGIGWG	823
Chenopodium quinoa	LQVSIISQALIFVTRARSWSFTERPGFLLVVAFLIAQLIASIISAEANWDLGGIRKIGWG	803
Carica papaya	LQVSIISQALIFVTRSRSWSYVERPGFLLMIAFLIAQLLATIIAVYANWGFARIKGCGWG	821
Ricinus communis	LQVSIVSQALIFVTRSRSWSFVERPGLLLIGAFIAAQLIATVIAVYANWGFARIEGCGWG	790
Punica granatum	LQVSIISQALIFVTRSRSWSFVERPGLLLVGAFIAAQLVATVIAVYASWLFARIEGIGWG	824
Nicotiana tabacum	LQVSIISQALIFVTRSRSWSFVERPGLMLVGAFFAAQLVATVLAVYANWEFARIKGVGWG	821
Brassica napus	LQVSTISQALIFVTRSRSWSFVERPGFLLVIAFLIAQLVASVIAAMATWPFAGIRSIGWG	831
Gossypium australe	LQVSIVSQALIFVTRSRSWSFIERPGLLLVVAFILAQLVATVLAVYANWGFARIKGIGWP	790
Arabidopsis thaliana	LQVSIISQALIFVTRSRSWSFVERPGALLMIAFLIAQLIATLIAVYANWEFAKIRGIGWG	818
Manihot esculenta	LQVSIVSQALIFVTRSRSWSYVERPGLLLVSAFIVAQLVATLIAVYANWGFARIKGCGWG	790
Malus domestica	LQVSIVSQALIFVTRSRSFSFMERPGMLLLGAFLIAQLIATLIAVYANWSFARIHGCGWG	790
Camellia sinensis	LQVSIVSQALIFVTRSRSWSLIERPGLLLVGAFLAAQLVATAIAVYANWEFARIQGIGWG	790
Zea mays	LQVSIVSQALIFVTRSRSWSFVERPGLLLVTAFLLAQLVATFLAVYANWGFARIKGIGWG	790
Theobroma cacao	LQVSIISQALIFVTRSRSWSFVERPGLLLIGAFIAAQLVATLIAVYASWGFARIQGIGWG	823
Sesamum indicum	LQVSIISQALIFVTRSRSWSFIERPGLLLVGAFLIAQLLATIIAVYAHWEFARIRGIGWG	821
Hevea brasiliensis	LQVSIISQALIFVTRSQSWSFMERPGVLLMCAFVVAQLVATLIAVYAHISFAYIRGIGWG	789
Glycine max	LQVSIISQALIFVTRSRSWSYVERPGILLITAFFAAQLVATVIAVYAHWDFARINGVGWG	790

Table S5: continued

Species	Sequence	Position
Neurospora crassa	AVVRIWIFSFGIFCIMGGVY----YILQDSVGFDNLMHGKSPKGNQKQRSLED----FVV	907
Coffea eugenioides	WGGVIWIFSIVTYFPLDILKFIIRYAL-SGKAWDSMIQNRTAFTTKKDYGREEREAQWAL	880
Spinacia oleracea	WTGVIWLYNIVTYMFLDPLKFAVRYAL-SGRAWHHMFNEKTAFTDKKSFGKGEREAAWAA	894
Cucumis sativus	WAGVIWIYSVIFYIPLDVLKFATRYAL-SGKAWNNMIQNRTAFTSKKDYGIGEREAQWAA	879
Hordeum vulgare	WAGVIWLFSIVFYFPLDIFKFFIRFVL-SGRAWDNLLQNKT-------------------	830
Jatropha curcas	WAGVIWLYSLIFYIPLDIIKF---------------------------------------	810
Triticum aestivum	WAGVIWLFSIVFYFPLDIFKFFIRFVL-SGRAWDNLLQNKT-------------------	830
Ananas comosus	WAGVIWLYSIVFYIPLDWIKFAIRYIL-SGRAWDNLLQNKTAFTTKKDYGREEREAQWAL	882
Chenopodium quinoa	WTGVIWLYNIVTYMLLDPLKF---------------------------------------	824
Carica papaya	WAGVIWVFSVITYIPLDVLKFIIRYAL-SGKAWDNLLQNKTAFTTKKDYGKGEREAQWAL	880
Ricinus communis	WAGVIWLYSVVTYVPLDLLKFAIRYIL-SGKAWDNLLENKTAFTTKKDYGKEE--------	842
Punica granatum	WAGAIWVFSIVTYLPLDVLKFIIRYSL-SGKAWDNLFQTHTAFSTKKDYGKNEREAQWAQ	883
Nicotiana tabacum	WAAVIWIYTIITYIPLDILKFIIRFAL-SGRAWDSMIQNKTAFTTKKDYGRGEREAQWAL	880
Brassica napus	WTGVIWVFNIVTYMLLDPIKFLVRYALISGKSWNRIVEQRTALNGKNNFGKDERMAAWAT	891
Gossypium australe	WAGVIWLYSIVFYIPLDVLKFLIRYAL-SGKAWDNLLQNKTAFTTKKDYGREE--------	842
Arabidopsis thaliana	WAGVIWLYSIVTYFPLDVFKFAIRYIL-SGKAWLNLFENKTAFTMKKDYGKEEREAQWAL	877
Manihot esculenta	WAGVIWLYSLVTYVPLDLLKFAIRYVL-SGKAWDNLLENKTAFTTKKDYGKEE--------	842
Malus domestica	WAGVIWIYSIVFYFPLDLMKFAIRYIL-SGKAWLNLLENKTAFTTKKDYGKEE--------	842
Camellia sinensis	WAGAIWVFSVVTYFPLDVLKFIIRYAL-SGKAWDNMIEDKATFTTNKDYS----------	839
Zea mays	WAGVVWLYSIVFYFPLDLIKFFIRFVL-SGRAWDNLLENKTAFTTKKDYGREE--------	842
Theobroma cacao	WAGVIWLFSLITYIPLDILKFIIRYSL-SGKAWDNLLQNKTAFTTKKDYGKGEREAQWAA	882
Sesamum indicum	WGGVIWIYSIVTYIPLDVIKFGIRFAL-SGKAWDSMIQNRTAFTTKKDYGKGEREAQWAM	880
Hevea brasiliensis	WAGVIWLYSLIFYIPLDIIKF---------------------------------------	810
Glycine max	WAGAIWVFSIVTYIPLDILKFLIRMGL-SGKAWDNMLDNKTAFTTKKDYGRGEREAEWAV	849

Species	Sequence	Position
Neurospora crassa	SL---QRVS-------------TQHEKSQ-------------------------------	920
Coffea eugenioides	AQRTLHGLQTPES-AGLFND-KHYRELSEIAEQAKRRAEVARLRELHTLKGHVESVVKLK	938
Spinacia oleracea	DRWTERGGGNSEDMRGLFSDRHSFRELNSMAEEARRRADIARLREQQSGRSQDKPQPSN-	953
Cucumis sativus	AQRTLHGLQPPET-SELFNDKTNYRELSEIAEQAKKRAEVARLRELHTLKGHVESVVKLK	938
Hordeum vulgare	--	830
Jatropha curcas	--	810
Triticum aestivum	--	830
Ananas comosus	AQRTLHGLQPPET-STLFSDKSSYRELSEIAEQAKRRAEVARLRELNTLKGHVESVVKLK	941
Chenopodium quinoa	--	824
Carica papaya	AQRTLHGLQPPEA-ALFNDK-NSYRELSEIAEQAKRRAEVARLRELHTLKGHVESVVKLK	938
Ricinus communis	--	842
Punica granatum	AQRTLHGLKHA---DALFQD-KNYRELSELADQAKRRAEMAKMTTQA-------------	926
Nicotiana tabacum	AQRTLHGLQTPEN-TGLFND-KNYRELSEIAEQAKRRAEVARLRELHTLKGHVESVVKLK	938
Brassica napus	EMRTQHGLETGQK---PMYERNGATELSSLAD----------------------------	920
Gossypium australe	--	842
Arabidopsis thaliana	AQRTLHGLQPKEA-VNIFPEKGSYRELSEIAEQAKRRAEIARLRELHTLKGHVESVVKLK	936
Manihot esculenta	--	842
Malus domestica	--	842
Camellia sinensis	--	839
Zea mays	--	842
Theobroma cacao	AQRTLHGLSPPET-IL--ND-KSYHELSEIAEQAKKRAEVARLRELHTLKGHVESVVKLK	938
Sesamum indicum	AQRTLHGLSTTET-PG--NE-NSYKELSEIAEQAKRRAEVARLRELHTLKGHVESVVKLK	936
Hevea brasiliensis	--	810
Glycine max	AQRTLHGLQVGES-NKAKQ-----HEQSE-------------------------------	872

Species	Sequence	Position
Neurospora crassa	-------------	920
Coffea eugenioides	GLDIETIQQHYTV	951
Spinacia oleracea	-------------	953
Cucumis sativus	GLDIETIQQHYTV	951
Hordeum vulgare	-------------	830
Jatropha curcas	-------------	810
Triticum aestivum	-------------	830
Ananas comosus	GLDIDTIQQHYTV	954
Chenopodium quinoa	-------------	824
Carica papaya	GLDIETIQQHYTV	951
Ricinus communis	-------------	842
Punica granatum	-------------	926
Nicotiana tabacum	GLDIETIQQHYTV	951
Brassica napus	-------------	920
Gossypium australe	-------------	842
Arabidopsis thaliana	GLDIETP-SHYTV	948
Manihot esculenta	-------------	842
Malus domestica	-------------	842
Camellia sinensis	-------------	839
Zea mays	-------------	842
Theobroma cacao	GLDIDTIQQHYTV	951
Sesamum indicum	GLDIETIQQHYTV	949
Hevea brasiliensis	-------------	810
Glycine max	-------------	872

Table S6. Lipid composition used in the coarse-grained molecular dynamics simulations.

Lipid name		Head group	Tail	Net charge	Content in inner leaflet (%)	Content in outer leaflet (%)
PIPC		Phosphatidylcholine	C16:0/18:2	0	11	28
DIPC		Phosphatidylcholine	di-C16:2-C18:2	0	5	14
PIPE		Phosphatidylethanolamine	C16:0/18:2	0	8	13
DIPE		Phosphatidylethanolamine	di-C16:2-C18:2	0	4	7
PIPA		Phosphatidic acid	C16:0/18:2	-2	11	11
DIPA		Phosphatidic acid	di-C16:2-C18:2	-2	5	5
PIPS		Phosphatidylserine	C16:0/18:2	-1	17	0
DIPS		Phosphatidylserine	di-C16:2-C18:2	-1	9	0
PIPI		Phosphatidylinositol	C16:0/18:2	-1	8	0
XNSM		Sphingomyelin	C(d24:1/24:1)	0	2	2
PVSM		Sphingomyelin	C(d18:1/18:1)	0	4	4
DPCE		Ceramide	C(d18:1/18:0)	0	3	3
PNCE		Ceramide	C(d18:1/24:1)	0	8	8
ERGO		Ergosterol	–	0	5	5
